# Compartmentalized microbes and co-cultures in hydrogels for on-demand bioproduction and preservation

**DOI:** 10.1038/s41467-020-14371-4

**Published:** 2020-02-04

**Authors:** Trevor G. Johnston, Shuo-Fu Yuan, James M. Wagner, Xiunan Yi, Abhijit Saha, Patrick Smith, Alshakim Nelson, Hal S. Alper

**Affiliations:** 1 Department of Chemistry, University of Washington, Box 351700, Seattle, WA USA; 2 Institute for Cellular and Molecular Biology, The University of Texas at Austin, Austin, TX USA; 3 McKetta Department of Chemical Engineering, The University of Texas at Austin, Austin, TX USA

**Keywords:** Biocatalysis, Metabolic engineering, Synthetic biology

## Abstract

Most mono- and co-culture bioprocess applications rely on large-scale suspension fermentation technologies that are not easily portable, reusable, or suitable for on-demand production. Here, we describe a hydrogel system for harnessing the bioactivity of embedded microbes for on-demand small molecule and peptide production in microbial mono-culture and consortia. This platform bypasses the challenges of engineering a multi-organism consortia by utilizing a temperature-responsive, shear-thinning hydrogel to compartmentalize organisms into polymeric hydrogels that control the final consortium composition and dynamics without the need for synthetic control of mutualism. We demonstrate that these hydrogels provide protection from preservation techniques (including lyophilization) and can sustain metabolic function for over 1 year of repeated use. This approach was utilized for the production of four chemical compounds, a peptide antibiotic, and carbohydrate catabolism by using either mono-cultures or co-cultures. The printed microbe-laden hydrogel constructs’ efficiency in repeated production phases, both pre- and post-preservation, outperforms liquid culture.

## Abstract (web summary)

Large scale suspension fermentation technology is not easily portable or reusable. Here the authors describe a hydrogel system suitable for long-term and reusable production with both single and multi-organism consortia.

## Introduction

Microbial production of value-added products ranging from small molecules to complex proteins is becoming increasingly attractive and effective across industry and academia^1–4^. Recent advances in synthetic biology have further enabled microbial production to be modular and distributed across multiple organisms, thus creating synthetic consortia that can reduce metabolic loads and afford more robust cell populations^5–7^. Though commonly employed in bioreactor and cellular interaction studies, the limitations of liquid culture systems are especially poignant when attempting to control the dynamics of a multi-organism consortium. Specifically, repeated (and sometimes even single) batch liquid co-cultures typically fail over time without sophisticated genetic control systems or particular nutrient conditions that seek to minimize the competitive growth bias that often occurs when utilizing disparate microorganisms^8–11^.

Immobilized cell technologies, wherein microbes are encapsulated within a polymeric matrix, have been developed as an alternative to suspension cell culture^12–16^. These microbe-laden matrices have been used to investigate quorum sensing between microbial species^14^, and as 3D architected ‘living materials’^15–17^. Calcium alginate and other polysaccharides are the most common matrices used for immobilizing cells, despite the sensitivity of the ionic crosslinks to the presence of charge-bearing molecules and the pH of the medium^18^. Various other hydrogel materials, comprised of synthetic or modified naturally occurring polymers, have been explored but fail to produce a material that is simultaneously readily processable, mechanically robust, and inert to the chemicals and biochemicals present in the media^19–29^.

Herein, we demonstrate a platform that utilizes a hydrogel system to compartmentalize microbes to build spatially segregated microbial consortia. To this end, Nelson and co-workers previously reported shear-thinning hydrogels based on F127-dimethacrylate (F127-DMA) polymer^16^. The F127-bisurethane methacrylate (F127-BUM) hydrogels employed in this study also exhibit a temperature-dependent sol–gel transition (~17 °C), which was used to immobilize yeast cells. The temperature response of the material facilitated the facile incorporation of the cells homogeneously throughout the hydrogel, while the shear-thinning behavior facilitated the extrusion of the cell-laden hydrogel from a nozzle.

**Fig. 1:**
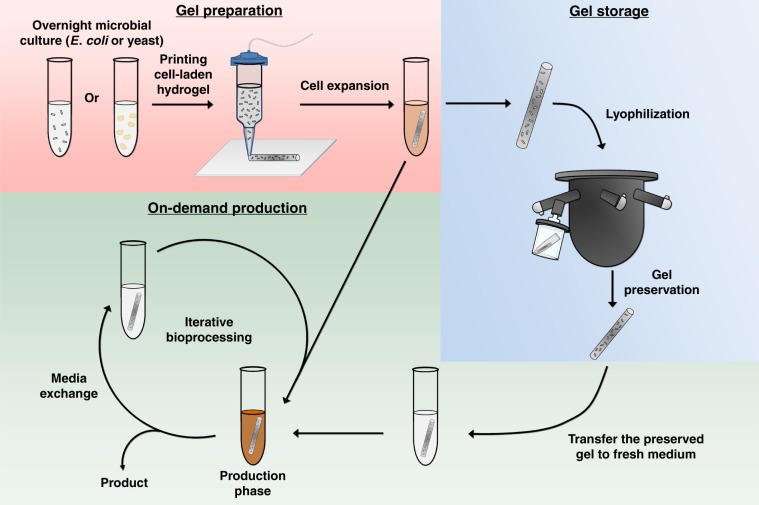
Overview of microbe-laden, extrusion-printed hydrogels for on-demand production. The hydrogel encapsulation and on-demand production process is divided into three parts. In the Gel Preparation stage, the printed and UV-cured microbial hydrogels are transferred to culture medium for cell outgrowth. While this initial outgrowth can also be used for production, the resulting cell-laden living materials can proceed to either the Gel Storage or On-Demand Production phase depending on user needs. In the Gel Storage stage, the microbial gels are treated with different types of preservation methods for storage and future use. The preserved gels are subsequently rehydrated and incubated in fresh medium to perform on-demand production, with iterative re-uses as desired.

In this work, we show that this F127-BUM hydrogel can be used to compartmentalize the various constituent organisms of an engineered microbial consortium that would otherwise be incompatible in a traditional liquid suspension culture. In doing so, we show that mono- and co-culture systems immobilized within hydrogels can be extrusion printed to form solid-state bioreactors capable of producing small molecules and antimicrobial peptides for multiple, repeated cycles of use. Interestingly, the microbe-laden hydrogels can also be preserved via lyophilization, stored in a dried state, and then rehydrated at a later time for on-demand chemical and pharmaceutical production (Fig. 1) in a manner that outperforms a traditional liquid-based culture format. Moreover, for co-culture systems, the spatial compartmentalization of the microbes enabled precise control of the consortium composition and dynamics without the need for genetically encoded mutualism.

## Results

### Encapsulation of microbes within F127-BUM Hydrogels

To test the repeated use and preservation capacity of these microbe-laden hydrogels, we conducted a series of demonstration tests using mono-cultures or co-cultures to produce a variety of important biochemical compounds and an antimicrobial agent. In each case, the microbe-laden hydrogel was based on 30 wt% F127-BUM, a polymer that forms a temperature responsive and shear-thinning hydrogel capable of encapsulating yeast (e.g., *Saccharomyces cerevisiae*) and/or bacteria (e.g., *Escherichia coli*) cells. While this material was not optimized based on the motility of encapsulated cells (such as cell leakage seen in Supplementary Fig. 8b), the polymer concentration 30 wt% of F127-BUM hydrogel was optimized for their stimuli-responsive behaviors that facilitated their processability^16^. The shear-thinning behavior of this hydrogel allowed the material to be cast into films or extruded as filaments from a nozzle. A photo-initiator was included in the hydrogel formulation to facilitate polymerization of the methacrylate functionalities of the polymer upon brief UV curing. The resulting microbe-laden hydrogels were mechanically robust and were suitable for multiple rounds of reuse without any visual signs of degradation. For these studies, mono- or co-culture hydrogels were evaluated for continuous production over multiple fermentation cycles, where we define the initial cell outgrowth stage as round 0 of gel use and the repeated, on-demand production phase (either immediately after outgrowth or post-preservation) as round 1 and onward (Fig. 1).

### On-demand productions using mono-culture laden hydrogels

**Fig. 2:**
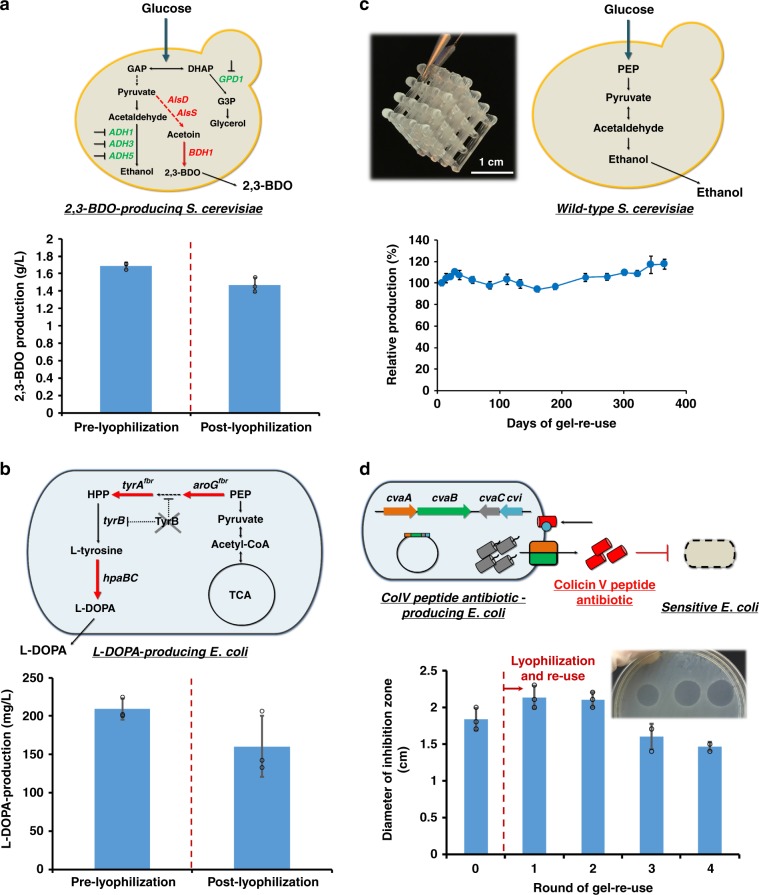
Re-use and preservation of mono-culture microbe-laden hydrogels. The fermentation performance of the printed hydrogel inks was tested for both bacterial (*E. coli*) and yeast (*S. cerevisiae*)-laden gels testing the production of 2,3-butanediol (**a**), l-DOPA (**b**), ethanol (**c**) and a peptide antibiotic (**d**). The production (pre- and post-preservation) is demonstrated in (**a**), (**b**), and (**d**). The production of ethanol from a year-long fermentation re-use process was evaluated using cell-laden hydrogel lattices (pictured top left in (**c**). Scale bar: 1 cm). Data are mean ± s.d.; *n* = 3 biological replicates for (**a**), (**b**) and (**d**); *n* = 4 biological replicates for (**c**). Source data are provided as a Source Data file.

As test cases, we selected the production of value-added biochemicals, 2,3-butanediol (2,3-BDO) and 3,4-dihydroxy-l-phenylalanine (l-DOPA) as examples, due to their market potential and their ability to evaluate two distinct metabolically engineered microbial hosts in these printed constructs^30,31^. First, a yeast-strain overproducing 2,3-BDO^32^ was encapsulated in the gel matrix and production (titer at 48 h in a test tube) was assessed both pre- and post-preservation (Fig. 2a). It should be noted that the lyophilization procedure also included a 10-min freezing step and vacuum drying without the use of any cryoprotectants. Nevertheless, after lyophilization, the preserved yeast-laden hydrogel retained nearly 90% of its BDO production capacity (1.5 ± 0.09 g L^−1^) compared to a paired sample prior to preservation (1.7 ± 0.04 g L^−1^). While these initial results were obtained for an *S. cerevisiae* BY4741-based strain, a similar phenomenon was also observed with an alternative strain constructed in the CEN.PK2 background (Supplementary Fig. 1 and Note).

Second, we evaluated the performance of the hydrogel using a metabolically engineered strain of *E. coli* designed to overproduce l-DOPA (Fig. 2b, Supplementary Fig. 2 and Note). In similar fashion to the 2,3-BDO yeast example, l-DOPA production from printed hydrogels did not differ significantly (*p* > 0.05, paired *t* tests) pre- and post-lyophilization (both producing >150 mg L^−1^ after 22 h). Collectively, these results demonstrate that yeast and bacteria-laden gels were both metabolically active, and that entrapment of cells in the F127-BUM hydrogel resulted in minimal loss of biocatalytic activity during lyophilization.

Third, we sought to evaluate the long-term stability and re-use potential of the cell-laden gels for continued, repeated production. To do so, we utilized a direct-write extrusion printer to create a yeast-laden hydrogel lattice (containing *S. cerevisiae* SO992) and evaluated its ethanol production capacity over the course of 1 year of repeated batch culturing (Fig. 2c). Through this 1-year period, the average titer achieved in these batches (8.74 ± 0.53 g L^−1^) exhibited no noticeable decrease in performance and, if anything, slightly increased throughout the process (Supplementary Fig. 3). It should be noted that all four replicate samples were still operational at the end of the year-long process, which demonstrated the robustness and potential longevity-enhancing capacity of these hydrogel materials.

Finally, we evaluated one additional mono-culture within our microbe-laden hydrogel constructs that demonstrated the ability of larger molecules (in this case, the peptide antibiotic, colicin V) to be produced and effectively diffuse out of the gel and into the surrounding culture media. To do so, we printed gels containing *E. coli* capable of secreting the pore-forming antibiotic colicin V (ColV)^33^ (Fig. 2d). For both the round 0 gel use and for four consecutive uses after lyophilization, the *E. coli*-laden hydrogel was able to produce colicin V at relatively constant levels as measured by a zone-of-inhibition assay. Furthermore, we sought to compare this gel-based performance to that of a traditional free-cell system culture process (Supplementary Fig. 4b). During this comparison, the level of ColV antimicrobial peptide produced by a free-cell system was inconsistent across iterative subculture cycles, unlike the microbe-laden hydrogel that retained more consistent production across repeated uses (Supplementary Note). Specifically, no zone-of-inhibition was observed for the free-cell system by the third round of re-use, and the bactericidal activity of the corresponding supernatant was dramatically reduced to 6.5% of its prior value. Collectively, these mono-culture results demonstrate that this hydrogel ink formulation can enable reusable and stable, on-demand production of both small molecules and peptide antibiotics using yeast or bacteria.

### Spatially compartmentalized microbial co-cultures

**Fig. 3:**
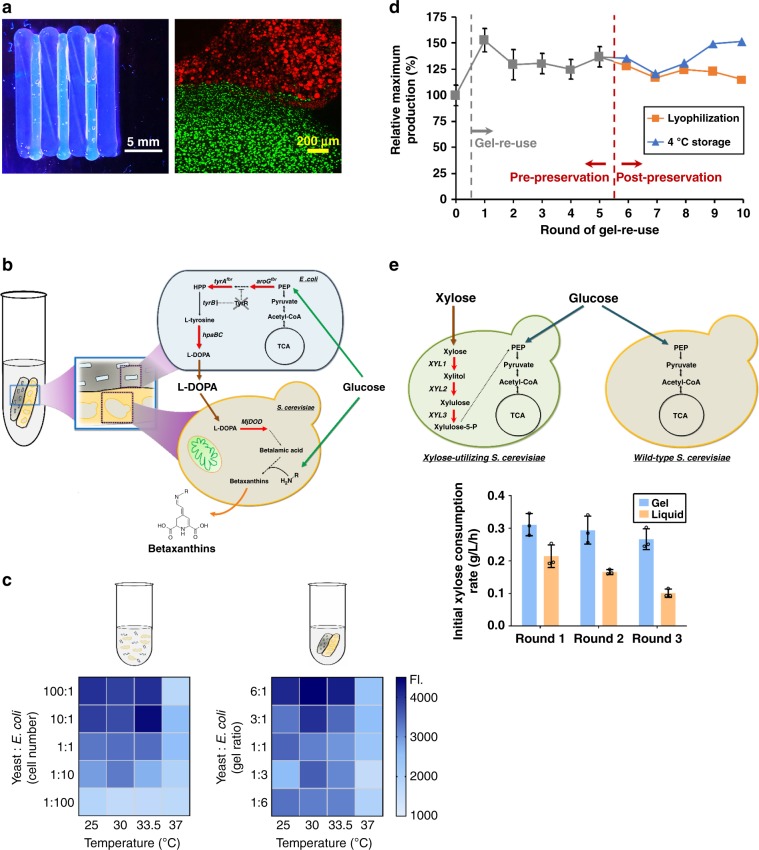
Spatially organized consortia in hydrogels outperforms tradition liquid co-culture systems. **a** Images of printed cell-containing hydrogel. Left: a UV-illuminated camera image of alternating stripes containing RFP yeast and GFP bacteria (scale bar: 5 mm). Right: confocal microscopy of 100 µm z-stack at the interface of these stripes. Little to no movement of cells out of their designed boundaries is observed at the interface (scale bar: 200 µm). **b** Schematic metabolic pathway of a commensal consortium *E. coli*–yeast for betaxanthins production. **c** Heat maps for betaxanthins consortia performance in the hydrogel and liquid-based culturing across altered cell number/gel ratios and fermentation temperatures. The color sale represents the intensity of betaxanthins fluorescence. **d** The reusability of 30 °C hydrogels with 6:1 (yeast:*E.coli*) gel ratio for betaxanthins production is compared pre- and post-lyophilization. **e** Glucose/xylose utilization via a parallel consortium with repeated use compared to liquid culture performance. Data are mean; *n* = 3 biological replicates for (**c**); data are mean ± s.d.; *n* = 3 biological replicates for (**e**); *n* = 2 biological replicates for (**d**) and gels after round 5 were split (*n* = 1) for examining the impact of lyophilization and refrigerated storage on betaxanthins production. Source data are provided as a Source Data file.

The experiments outlined thus far illustrate the use of mono-culture based microbe-laden hydrogels. However, compartmentalizing a consortium through simple spatial separation within printed hydrogel constructs may provide a facile manner to control dynamics, especially for the endpoint balance within a co-culture. Natural microbial consortia rely upon spatial organization, as demonstrated by insect hindguts and biofilms^34^. In this regard, a synthetic microbial consortium that mimics these natural systems should provide both spatial organization as well as a protective environment for the microbes to thrive non-competitively. Moreover, achieving stable liquid co-cultures is challenging due to different competitive growth rates exhibited by many organisms, especially at different temperatures. This behavior is evident in a simple *S. cerevisiae* / *E. coli* co-culture expressing RFP and GFP, respectively, where one organism quickly dominates the other in a temperature-dependent manner (Supplementary Fig. 5). As an alternative, the printing of microbe-laden hydrogels that spatially compartmentalize each organism can minimize or remove this competition. For example, when an alternating striped pattern of RFP-producing *S. cerevisiae* and GFP-producing *E. coli* laden hydrogels was printed, confocal imaging and fluorescent macroscopy (i.e. macroscopic fluorescence imaging of the gel) showed that cell colony expansion was localized to the respective yeast and bacterial regions (Fig. 3a and Supplementary Fig. 6). Moreover, these gels did not impede cell growth when co-cultured, as the yeast samples achieved a maximum confluence of 93.5% and the bacterial samples achieved a maximum confluence of 88.6% in their respective hydrogel compartments relative to similar mono-culture gels (Supplementary Fig. 6a). Importantly, the physical segregation and hydrogel-based immobilization of the distinct microbial species offers advantages in controlling consortium population dynamics when compared to a mixed liquid culture system. The microbe-laden hydrogels can be deposited via deposition from a nozzle, which allows the final consortium composition to be specified over a broad range by simply changing the amount of each respective gel extruded, thus enabling a plug-and-play approach to consortium bioprocessing. Furthermore, the volume of gel and its spatial pattern can be digitally controlled using a direct-write extrusion printer. To test the efficacy of our hydrogel inks to spatially organize stable consortia, we explored two different types of consortia: (i) a commensal consortium for betaxanthins production and (ii) a parallel consortium for more efficient glucose and xylose utilization in fermentation.

For a commensal consortium, we demonstrated the production of betaxanthins, a natural food colorant^35^, through simple combination of an engineered *E. coli* and *S. cerevisiae* strain together to form a gel-based consortium (Fig. 3b and Supplementary Note). To do so, a l-DOPA producing bacteria-laden hydrogel was printed alongside a yeast-laden hydrogel that contain engineered yeast capable of converting l-DOPA into betaxanthins. No further synthetic engineering was conducted on these strains to enable mutualism or matched growth rates. To provide a qualitative comparison with liquid culture, we analyzed the performance of this consortium by assessing production of betaxanthins across a range of temperatures and cell ratios (enabled by altered inoculum ratios for the liquid culture and respective gel masses for the hydrogel system). Even in the round 0 condition (when both species were grown to confluence), production by the hydrogel consortium surpassed the liquid suspension culture for most conditions and had a more consistent production response curve, especially across a range of temperatures (25.0–30 °C) (Fig. 3c and Supplementary Fig. 7). A difference in the optimal temperature is seen between the free-cell liquid suspension culture and hydrogel systems and is likely the result of cell growth competition that exists in the free-cell system—a constraint that is relieved when using this hydrogel system. Although the use of the hydrogel provides spatial segregation of the co-culture members, total nutrients fed to the system can ultimately be limiting. Nevertheless, one of the advantages of this system is the ability to specify the final desired consortia composition by altering the ratio of cell-laden gels. In this regard, the nutrient limitation issue can be solved by establishing a consortia ratio that favors either *E. coli* or *S. cerevisiae* for a given condition as demonstrated above. Therefore, the issues of imbalanced biosynthesis capacities can be addressed by changing this cell-laden gel volume.

**Fig. 4:**
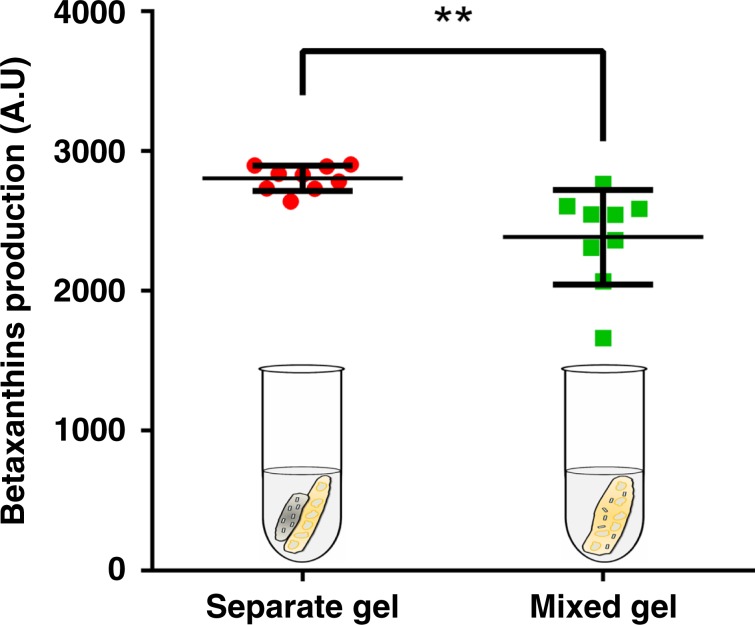
Spatial organization of microbial consortia improves on-demand production over a mixed gel. Betaxanthins production was compared between separate (printing *E. coli* and yeast gel separately) and mixed (mixing two species in the same gel) hydrogels. Both separate and mixed gels encapsulated the same initial number of consortia cells. Data are mean ± s.d.; *n* = 9 biological replicates. **p-value (0.0054) for the means of two groups was calculated using unpaired *t* test with Welch’s correction. *p*-value (0.024) for the standard deviations of two groups was calculated using the Levene’s test. Source data are provided as a Source Data file.

To further demonstrate the importance of spatial segregation of the consortium members for a stable co-culture system, we evaluated the metabolic activity of a spatially separated consortia hydrogel as described above with that of a mixed gel (i.e. both organisms were mixed and combined together into a single hydrogel) (Fig. 4). The spatial organization of consortia members within separate hydrogels significantly improved the betaxanthins production compared with mixed gels (*p* = 0.0054, unpaired *t* test with Welch’s correction) and had a substantially lower variance between samples (*p* = 0.024, Levene’s test), suggestive of the fact that competitive cell growth still occurs even within the structure of a hydrogel and this can only be bypassed with spatial separation afforded by distinct gels for each organism, as explored here.

Next, this consortium-based gel approach was also evaluated on the basis of reusability and on-demand production (Fig. 3d). Whereas the liquid culture quickly lost its ability to produce betaxanthins due to unstable co-culturing (Supplementary Fig. 8), the metabolic activity of the immobilized consortium retained over 100% of the production compared to the round 0 hydrogel across five continuous re-use cycles at 33.5 °C (Supplementary Fig. 9b). In fact, higher production was seen after round 0, possibly because the consortium was already mature and established, and thus the gels could more readily achieve a faster biocatalytic rate and economize sugar conversion (Supplementary Fig. 8e and 9b). These promising results for reusability prompted us to further investigate preservation of microbial consortium-laden hydrogels for on-demand production of betaxanthins. In this case, lyophilization and re-use (similar to the scheme previously describe for mono-cultures) resulted in essentially the same production level over five subsequent fermentations (Fig. 3d), demonstrating that microbe-laden hydrogels containing co-cultures could function for a culturing period of at least 2 months. As with the ethanol mono-culture example above, all hydrogel samples were still operational at the end of this time period.

To complement these preservation results, we additionally evaluated the impact of both refrigerated storage and liquid nitrogen freezing on these consortium-containing gels (Fig. 3d and Supplementary Fig. 9b). The refrigerated storage treatment exhibited no loss in productivity for an additional five rounds of re-use, whereas cryopreservation samples showed a mild reduction in metabolic activity, to 85% of the original productivity (Supplementary Fig. 9b, and Supplementary Note). In contrast, the liquid culture provided an inconsistent production across all preservation methods used in this study (Supplementary Fig. 9c, d).

Next, we sought to evaluate the impact of storage on production and gel integrity. Specifically, we observed that the lyophilized consortia hydrogel maintained 100% efficiency of betaxanthins upon rehydration even after long-term storage of the lyophilized at room temperature for 3 months (Supplementary Fig. 9e). Furthermore, the lyophilization process had minimal impact on the mechanical integrity of the samples. The Young’s moduli before and after preservation were identical for the material (Supplementary Fig. 9f top), and SEM images indicated that there was little change in the microstructure (Supplementary Fig. 9f bottom). The cell distribution within the material after rehydration was also unchanged (Supplementary Fig. 9g). We observed that the overall cell leakage rate from the gel actually decreased after lyophilization and rehydration compared to the pre-lyophilized condition (Supplementary Fig. 9h). In sum, this F127-BUM hydrogel system provides for a strong preservation capacity of consortia.

As a final benchmarking, we sought to provide a comparison of our F127-BUM hydrogel with standard calcium alginate encapsulation using the same consortium. Calcium alginate hydrogels require constant replenishing of calcium ions in the reactor media in order to retain the charged crosslinks between the individual polymer strands. Additionally, the charged nature of the polymer itself may lead to unwanted interactions between the hydrogel and fermentation products, possibly interfering with diffusion out of the hydrogel^18^. In this comparison, we found a greater than 2.5-fold improvement in betaxanthins production for microbe-laden F127-BUM-based hydrogel when compared to microbe-laden calcium alginate (Supplementary Fig. 10). Moreover, we observed that the microbe-laden calcium alginate gels softened and degraded slightly over a 72-h incubation period, whereas the microbe-laden F127-BUM hydrogels retained structural integrity. These results demonstrate the re-use and stability of this platform compared with commonly used calcium alginate gels.

As a final demonstration, we assembled a parallel yeast–yeast strain consortium to enable glucose and xylose fermentation. In this case, we established a parallel consortium hydrogel comprised of a glucose-consuming wild-type *S. cerevisiae* S288C and a xylose-utilizing yeast YSX3^36^ and compared net sugar utilization to that of a free-cell, liquid culture consortia (Fig. 3e and Supplementary Fig. 11). As in the betaxanthins example above, there is an implicit selection pressure in this system wherein the faster growing S288C wild-type strain will be enriched in glucose-rich conditions. The free-cell system at this condition exhibited a lower average xylose consumption rate (around 0.07 g L^−1^ h^−1^) (Supplementary Fig. 11 c; left) compared with the liquid culture conducted in YPDX for round 0 (about 0.16 g L^−1^ h^−1^) (Supplementary Fig. 11 c; right), suggesting that suspension yeast cultures grown in YPD after round 0 probably were already self-selected to possess a higher proportion of wild-type, non-xylose consuming yeast (as this strain exhibits faster, more robustly growth). In contrast, the gel-based consortium mitigated this selection pressure and maintained a consistent xylose consumption rate of nearly 3.5-fold over the liquid culture condition when cells were initially cultured on glucose (Supplementary Fig. 11a). Even when initial growth was conducted in a more xylose-strain permissive condition (such as YPDX in Fig. 3e), the gel-based consortium still outperforms the liquid culture format and remains stable, whereas the liquid consortium fails rather rapidly after only three subculture cycles (Supplementary Note).

While all experiments were conducted at laboratory scale, we envision its utilization in a perfusion style reactor in which media is constantly flowed through the immobilized lattice, allowing constant flow and production of target molecules without the need for reactor downtime. Alternatively, these materials can also be employed on a smaller scale, in a modular production scheme. This approach would allow for easy, mobile deployment of the hydrogel constructs for production of smaller volumes of the target compounds. When aligned with the ability to preserve and rehydrate the materials for continued bioprocessing, we believe that these hydrogels could one day allow for the implementation of immobilized bioreactors in more extreme or remote settings, where access to bioreactor technology and bulk resources is otherwise difficult or impossible.

## Discussion

We have developed a microbe-laden hydrogel platform that can compartmentalize and spatially organize individual microbial populations and consortium members into hydrogel constructs for the production of both small molecules and active peptides. The approach enables repeated re-use and preservation through refrigeration or lyophilization, thus enabling on-demand production of these molecules in a manner that is unmatched by traditional liquid-based culturing. The ability to enable long-term stability of cells and consortia (up to 1 year in the continuous fermentation of yeast to produce ethanol and at least 3 months in the case lyophilized gel storage and subsequent use for betaxanthins) provides a niche for preserving catalytic function in industrial bioprocesses. Moreover, the ability to control consortium dynamics simply by changing the amount of hydrogel ink printed provides a newfound capacity for plug-and-play synthetic consortia. Looking forward, this strategy enables a portable, reusable, and on-demand capacity for small molecule and pharmaceutical production from a variety of microorganisms.

## Methods

### Strains, media, and plasmid construction

All strains, plasmids and primers used in this study are listed in Supplementary Table 1 and Table 2. NEB10β was used for gene cloning or propagation of all expression vectors. It was cultivated in LB medium supplemented with appropriate antibiotics (100 μg mL^−1^ ampicillin, 50 μg mL^−1^ kanamycin or 50 μg mL^−1^ spectinomycin (Sigma)) with 225 rpm orbital shaking at 37 °C. Starter cultures of yeast strains were routinely grown in yeast synthetic complete (YSC) media with the appropriate dropouts for auxotrophic selection. YPD (10 g L^−1^ yeast extract, 20 g L^−1^ peptone and 20 g L^−1^ glucose) and YPDX (10 g L^−1^ yeast extract, 20 g L^−1^ peptone, 15 g L^−1^ glucose and 15 g L^−1^ xylose) media were used in glucose/xylose utilization studies. LBYSD (1X LB, 1X YSC + CSM-URA, 10 mM vitamin C, 50 µg mL^−1^ kanamycin and 50 µg mL^−1^ spectinomycin) and M9YSD (1X M9, 2 mM MgSO_4_, 0.1 mM CaCl_2_, 1X YSC + CSM-URA, 10 mM vitamin C, 50 µg mL^−1^ kanamycin and 50 µg mL^−1^ spectinomycin) were used for medium optimization studies.

Oligonucleotide primers used for PCR amplification were purchased from Integrated DNA Technologies (Coralville, IA). All ligated or Gibson-assembled DNA^37^ were electroporated (2 mm Electroporation Cuvettes, Bioexpress) into *E. coli* competent cells with a BioRad Genepulser Xcell at 2.5 kV. The Frozen EZ Yeast Transformation II Kit (Zymo Research) was used to transform an integrative cassette into the yeast. For tyrosine production, Gibson assembly method was employed to combine an amplicon containing two T7 promoters amplified from pRSFDuet-1 (Addgene) with primers 11 as well as 12 and the PCR product amplified from pET28b empty vector (Addgene) with primers 13 and 14 to construct pET28b-Duet-1. To construct pCDF-pT7-*tyrA*^*(fbr)*^-pT7-*aroG*^*(fbr)*^, the FRT-flanked kanamycin resistance gene on the plasmid pCDF-Kan^FRT^-*tyrA*^*(fbr)*^-*aroG*^*(fbr)*^ was removed using primers P7-P10 via Gibson assembly. To construct pCDFDuet-1, two T7 promoters amplified from pRSFDuet-1 with primers 11 and 12 was Gibson assembled with the PCR product amplified from pCDF-pT7-*tyrA*^*(fbr)*^-pT7-*aroG*^*(fbr)*^ with primers 13 and 14. To construct pET28-pT7-*aroG*^*(fbr)*^-tT7, the DNA fragment PCR-amplified from pCDF-pT7-*tyrA*^*(fbr)*^-pT7-*aroG*^*(fbr)*^ with primers P25 and P26 was cloned into the amplicon amplified from pET28b empty vector with primers P27 and P28. For pET28-pYIBN-*aroG*^(fbr)^ construction, native promoter *yibN* and DNA fragment containing *aroG*^(fbr)^ gene was PCR-amplified from *E. coli* MG1655 genomic DNA with primers P33 as well as P34 and from pET28-pT7-*aroG*^*(fbr)*^-tT7 with primers P35 as well as 36, respectively. The resulting two amplicons were then cloned into the PCR product amplified from pET28b-Duet-1 with primers P37 and 38 using Gibson assembly. To construct pET28-pYIBN-*aroG*^(fbr)^-B30rbs-*tyrA*^(fbr)^-tRRNC, DNA fragment containing *tyrA*^*(fbr)*^ PCR-amplified from pCDF-pT7-*tyrA*^*(fbr)*^-pT7-*aroG*^*(fbr)*^with primers P41 as well as P42 was combined with primer 43 and cloned into the amplicon obtained from pET28-pYIBN-*aroG*^(fbr)^ with primers P39 as well as P40. For l-DOPA production, primer set P69 and P70 were used for amplifying *HpaB-HpaC* from BL21 (DE3) genomic DNA, and the resulting amplicon as well as primer 67 were Gibson assembled with the PCR product amplified by using primers P72 and P73 from the template pCDFDuet-1 to construct pCDF-pLPP-B30rbs-*hpaB*-*hpaC*-T7t. For the construction of pCDF-pLPP-B32rbs-*hpaB*-*hpaC*-T7t, primer set P70 and P71 were used for amplifying *HpaB-HpaC* from BL21 (DE3) genomic DNA, and the resulting amplicon as well as primer 68 were Gibson assembled with the PCR product amplified by using primers P72 and P73 from the empty vector pCDFDuet-1. For betaxanthins production, primer set P74 and P75 were first used for amplifying *MjDOD* gene from pCMC0759. The resulting amplicon was then linearized with restriction enzymes SpeI as well EcoRI and ligated with SpeI/EcoRI digested mumberg plasmid p416-pGPD-tPRM9 to yield p416-pGPD-*MjDOD*-tPRM9. To generate an URA3 integrative cassette, primers P78 and P79 were used for amplifying a linear DNA fragment with 65 bp-long *Leu2* homology arms from p416-pGPD-*MjDOD*-tPRM9.

### Polymer synthesis/functionalization

Glassware was oven-dried at 125 °C for at least 16 h. F127 (60 g, 4.8 mmol) was dried under vacuum (~2 Pa) for at least 16 h at room temperature in a round-bottom flask. Anhydrous CH_2_Cl_2_ (550 mL) was charged to the flask under an N_2_ atmosphere. The mixture was stirred at 30 °C, and following complete dissolution of the F127, dibutyltin dilaurate (12 drops) was added using a glass Pasteur pipette. The 2-isocyanatoethyl methacrylate (3.5 mL, 24.8 mmol) was diluted in anhydrous CH_2_Cl_2_ (50 mL) and was added to the reaction mixture at a rate of approximately 1 drop/s. The reaction was allowed to proceed while stirring under dry N_2_ at 30 °C. After 2 days, the reaction was quenched by the addition of MeOH (60 mL), and the mixture was concentrated at 30 °C using a rotary evaporator. The F127-BUM was precipitated in Et_2_O (2000 mL). During the precipitation, Et_2_O was stirred in a large conical flask, and the concentrate was poured in slowly. The precipitate mixture was stirred for an additional 15 min before separation via centrifugation. Eight 50 mL centrifuge tubes were filled with precipitate mixture and centrifuged (3000*g*) for approximately 10 min. The transparent supernatant in each tube was decanted from the F127-BUM pellet, and more of the precipitate mixture was added on top of each pellet. This process was repeated until all of the precipitate had been collected in the eight centrifuge tubes, and all of the solvent had been discarded. The F127-BUM precipitate was then washed twice. Each wash was performed by adding Et_2_O (approximately 30 mL) to each pellet and vortex-mixing until redispersion of the precipitate was observed; redispersion was followed by centrifugation (3000*g*) for re-separation of the F127-BUM precipitate, and supernatants were again discarded. After washing, the F127-BUM from each tube was pooled and transferred to a large beaker. Excess ether was allowed to evaporate while agitating the F127-BUM with a spatula under an N_2_ atmosphere. The resultant F127-BUM powder was dried fully overnight at room temperature under vacuum (~2 Pa) and stored in the dark at 4 °C until further use.

### Materials generation and processing

F127-BUM (3 g) was dissolved in sterile, deionized water (7 g) and the resulting mixture was cooled overnight at 5 °C to facilitate complete dissolution of the polymer. Then, the solution was warmed to room temperature (21 °C) to induce the sol-to-gel transition. The hydrogels used for extrusion printing were mixed with the photo-radical initiator 2-hydroxy-2-methylpropiophenone (10 μL for every 10 g of hydrogel solution). For the preparation of microbe-laden hydrogels, the aforementioned 30 wt% hydrogel was cooled to 5 °C in a refrigerator. At this temperature, the hydrogel underwent a gel-to-sol transition, affording a free-flowing liquid. Yeast or bacteria cells were added from liquid culture, at the desired concentration, as determined by OD600 measurements. The resulting solution was mixed thoroughly and allowed to equilibrate at 5 °C until all of the bubbles present in the solution were removed. Finally, the hydrogel was warmed to 21 °C to undergo a sol-to-gel transition, to afford a shear-responsive gel. These cell-laden materials were then extruded through the end of a syringe in cylindrical samples of the appropriate microbial hydrogel. The hydrogels could then be photocured with an exposure to 365 nm light (at 3.4 mW cm^−2^) for 90 s.

### Extrusion of microbial hydrogels

For hand-extruded hydrogel samples, the microbial hydrogels were transferred while cold (~5 °C) into 5 mL syringes. Once the hydrogel was inside the syringe, the samples were warmed to 21 °C to afford a shear-responsive hydrogel. These hydrogels could then be extruded through the end of the syringe by applying back pressure to the hydrogel sample with the syringe plunger. Samples were deposited in increments of 250 μL. For co-culture gels, we deposited the samples side-by-side prior to UV curing. Due to the self-healing nature of the pre-cure hydrogels, this approach allows the final construct (after UV curing) to be comprised of a single continuous hydrogel, but with spatially distinct regions, each containing a particular microbial strain. For CAD model extrusion printing, a modified pneumatic direct-write extrusion printer was assembled based on a Tronxy P802E 3D Printer kit, from Shenzen Tronxy Technology Co. The printer was controlled with an Arduino using the Marlin firmware. All CAD models were designed in OpenSCAD. G-code commands for the printer were generated using Slic3r. The resulting G-code was modified using Python to introduce required commands for the dispensing of the hydrogel via pneumatic pressure. All printing was performed using a 30 wt % hydrogel ink with an extrusion air pressure of 20 psi, a print speed of 15 mm s^−1^, and a 0.21 mm inner diameter CML Supply conical nozzle attachment. Upon completion of the extrusion printing, the structures were irradiated under 365 nm light (at 3.4 mW cm^−2^) for 90 s to cure and chemically fix the structures.

### Imaging and co-culture flow cytometry

For optical imaging, images of the extrusion printed hydrogels were captured using an iPhone X. For confocal imaging, the microscopy images were taken using a Leica TCS SP5 II laser scanning confocal microscope. Confocal microscopy output images were processed using ImageJ Java software.

Cell segregation studies were conducted by printing strands of yeast (*S. cerevisiae* yJS001)- and bacteria (*E. coli* CD02)-laden hydrogel in contact with one another, by utilizing the two-material printing capabilities of the pneumatic-driven extrusion printer. Bacteria and yeast were both seeded at concentrations of 10^7^ cells/g of hydrogel. Strands of the two hydrogel samples were printed in contact with one another and cured. The resulting structures were submerged incubated at 30 °C in 1:1 SC:LB media and imaged under confocal microscopy at 1, 3, and 7 days. The interface between the strands was imaged across 100 µm, with images taken at steps of approximately 3 µm. The resulting z-stacks were merged into one image to ensure the integrity of the interface through the thickness of the printed structure. E. coli used in this study are shown in the green channel, and yeast are shown in the red channel. All images were taken with a dry ×10 objective. RFP fluorescence was excited with a 561 nm laser at 5% laser power, and emission was scanned from 569 to 700 nm. GFP fluorescence was excited with a 488 nm laser at 5% laser power, and emission was scanned from 500 to 550 nm.

For confluence imaging, standard samples of bacteria (*E. coli* CD02)-laden and yeast (*S. cerevisiae* yJS001)-laden hydrogels were prepared in the same manner as previously discussed. Individual samples of each type of hydrogel were printed and cured. The yeast samples were incubated in SC media at 30 °C, while the bacteria samples were incubated in LB media at 37 °C. These samples were incubated in their respective shakers for 7 days. At the end of the incubation, the samples were imaged under confocal microscopy, and the imaging settings were standardized and saved. For the co-culture samples, bacteria- and yeast-laden hydrogels were printed in the same fashion as the cell segregation study. These samples were then incubated in 1:1 LB:SC media, and placed into shakers at the five temperatures explored in our fermentation studies (25, 30, 33.5, 37, 40 °C). These samples were extracted and imaged at 1, 3, and 7 days, using the same settings that were established in the standard sample imaging. Lastly, the percent area of fluorescence in all the images was quantified using ImageJ, and the percent confluence of the various conditions of co-culture were calculated against the standard samples. All images were taken with a dry ×10 objective. RFP fluorescence was excited with a 561 nm laser at 5% laser power (850 gain), and emission was scanned from 569 to 700 nm. GFP fluorescence was excited with a 488 nm laser at 10% laser power (850 gain), and emission was scanned from 500 to 550 nm.

*E. coli* CD02 and *S. cerevisiae* JMW001 were used for co-culture experiments analyzed using flow cytometry. Flask cultures containing GFP-expressing *E. coli* and RFP-expressing *S. cerevisiae* (initially seeded at 1.5E6 cells/mL each) were grown for 20 h at various temperatures in identical shaking incubators with agitation set to 225 RPM. Culture samples were then diluted in DPBS and held on ice to minimize further growth during analysis. Flow cytometry was performed using a BD LSRII Fortessa instrument and BD FACSDiva software with GFP and mCherry (RFP) acquisition settings. Gates were initially drawn based on FSC and SSC signals, and the percentages of each cell type in the resulting scatter-based gates were confirmed based on fluorescence (i.e. GFP for *E. coli*, RFP for *S. cerevisiae*). This analysis was completed for triplicate cultures started from the same frozen vial of *E. coli* and *S. cerevisiae*.

### Ethanol production

*S. cerevisiae* SO992 was used for ethanol production. To monitor the long-term fermentation of glucose to ethanol, we utilized extrusion printed hydrogel cubes with a yeast cell concentration of 10^8^ cells/g of hydrogel. The printed cubes were washed twice using SC media, and stored individually in tubes containing 10 mL of synthetic complete media for each gram of living material. These tubes were degassed for 6 min by bubbling argon through the solution to ensure anaerobic conditions for the fermentation process. Finally, the tubes were placed into a 30 °C shaker for the fermentation incubation. The media was replaced every 3−4 days and stored for ethanol quantification measurements by gas chromatography. This study was performed over the course of 365 days.

### Tyrosine production

A lambda-red recombination-based method was employed to delete chromosomal transcriptional regulator *tyrR* in *E. coli* BL21 (DE3) strain^38^. Primers P1 and P2 were used to PCR-amplify an FRT-flanked kanamycin resistance gene (FRT-KanR) from the plasmid pKD13. The resulting PCR product was then be used as a template and primers P3 as well as P4 were utilized for PCR amplification. The amplified FRT-KanR cassette carried 100 bp of homology with the ends of chromosomal *tyrR* to facilitate knockout efficiency. Following transformation of the knockout cassette into *E. coli*, colonies were verified by colony PCR and sequencing with primers P5 and P6. Excision of FRT-KanR from the resulting strain *E. coli* Δ*tyrR* was mediated by transformation with pCP20 expressing FLP recombinase as described in the literature^38^. Colonies were verified by colony PCR and elimination of temperature-sensitive pCP20 was carried out via nonselective incubation at 43 °C. To ensure FRT-KanR excision and knockout of *tyrR*, the gene fragment PCR-amplified from *E. coli* Δ*tyrR* genomic DNA prepared using the Wizard Genomic DNA Purification Kit (Promega, Madison, WI) was then sequence verified via Sanger sequencing. The resulting *E. coli* BL21 (DE3) Δ*tyrR* strain was designated as eBL01. The tyrosine producing plasmid pET28-pYIBN-*aroG*^(fbr)^-B30rbs-*tyrA*^(fbr)^-tRRNC was transformed into eBL01 leading to an eBL04 strain. For tyrosine production, starter culture of *E. coli* was grown in 2 mL LB medium containing 50 μg mL^−1^ kanamycin with 225 rpm orbital shaking at 37 °C overnight. Then seed culture was inoculated into 2 mL M9 medium supplemented with 2% glucose as well as 50 μg mL^−1^ kanamycin with an initial OD_600_ of 0.05 and incubated at 37 °C. After 18 h fermentation, suspension culture was centrifuged at 16,000*g* for 2 min and supernatant fraction was collected for measurement of tyrosine production using HPLC. The cell growth was measured by Ultrospec 2100 Pro UV/Visible Spectrophotometer observing optical density at 600 nm. The recombinant strain produced 0.44 mM of tyrosine. The results represented the mean ± S.D. of three biological replicates.

### l-DOPA production

The l-DOPA producing plasmids listed in Supplementary Table 1 were individually transformed into tyrosine producer *E. coli* eBL04. The eBL04 transformed with empty vectors pET28b-Duet-1 and pCDF-Duet-1 resulting in a control strain was used in this experiment. For l-DOPA production, cells were precultured in 2 mL LB medium containing 50 μg mL^−1^ kanamycin as well as 50 μg mL^−1^ spectinomycin with 225 rpm orbital shaking at 37 °C overnight. Seeding cultures were then transferred to 3 mL LB medium supplemented with antibiotics with an initial OD_600_ of 0.05 and incubated at 30 °C for 15 h. After fermentation, cultures were pelleted at 16,000 g for 2 min and media supernatant were collected for measurement of l-DOPA production using HPLC. For l-DOPA production in *E. coli* with lyophilization and re-use, the starter culture of highest producer *E. coli* eBL0430D was inoculated from glycerol stocks into 3 mL LB medium containing 50 μg mL^−1^ kanamycin and 50 μg mL^−1^ spectinomycin and incubated at 37 °C overnight. The details of preparation of hand-extruded hydrogel samples were described above. Briefly, 4.5 × 10^7^ overnight cells were encapsulated in 0.3 g of polymer. The printed and cured cell-laden gels were then incubated in 3 mL LB medium containing 50 μg mL^−1^ kanamycin and 50 μg mL^−1^ spectinomycin with 225 rpm orbital shaking at 37 °C for 22 h. Subsequently, the gel samples were treated with lyophilization process as described below. The preserved gels were next transferred to 3 mL LB medium with appropriate antibiotics and incubated at 37 °C for 22 h. Cultures were collected at the end of each batch and supernatant fractions from pelleted samples were filtered and analyzed by HPLC for l-DOPA quantification. The results represented the mean ± S.D. of three biological replicates.

### 2,3-Butanediol production

For 2,3-BDO production in yeast with lyophilization and re-use, the starter cultures of 2,3 BDO-producing BY4741 and CEN.PK2-a yeasts were inoculated from glycerol stocks^32^ into 3 mL YSC dropout media (CSM-URA-LEU) and incubated at 30 °C for 60 h. 4.5 × 10^7^ overnight yeast cells were afterwards encapsulated in 0.3 g of polymer. The details of preparation of hand-extruded hydrogel samples were described above. The printed and cured cell-laden gels were then incubated in 3 mL YSC + CSM-URA-LEU at 30 °C for 72 h. Subsequently, the gel samples were treated with lyophilization process as described below. The lyophilized gels were then transferred to 3 mL YSC + CSM-URA-LEU and incubated at 30 °C for 72 h. In each batch, 200 uL culture samples were taken for every 24 h and the concentrations of 2,3-BDO and acetoin in supernatant part from pelleted cells were analyzed by HPLC. The results represented the mean ± S.D. of three biological replicates.

### Antimicrobial peptide production

*E. coli* MC4100_pHK11^33^ and the sensitive strain DH5α_pET28b were used in this study. For antimicrobial peptide colicin V production, toxin-producing cells were precultured in 3 mL LB medium supplemented with 100 μg mL^−1^ ampicillin at 37 °C for 15 h with 225 rpm orbital shaking. The control strain was routinely cultivated in 3 mL LB medium with 50 μg mL^−1^ kanamycin at 37 °C for 15 h to maintain cell growth. 4.5 × 10^7^ toxin-producing cells were afterwards immobilized in 0.3 g of hydrogel. The printed and cured cell-laden living materials were then incubated in 3 mL LB medium containing 100 μg mL^−1^ ampicillin at 37 °C. Subsequently, the gel samples were proceeded to lyophilization treatment as described in the preservation section below. The preserved gels were next transferred to 3 mL LB medium with 100 μg mL^−1^ ampicillin and incubated at 37 °C. Four consecutive uses after lyophilization were performed. For round 0 in free-cell system, 4.5 × 10^6^ toxin-producing cells were transferred from overnight culture to 3 mL LB medium containing 100 μg mL^−1^ ampicillin and incubated at 37 °C. Four repetitive uses were subsequently carried out through transferring 50 μL of bacterial suspension from previous batch to the next batch. In each batch, samples at the 21-h timepoint were taken for zone of inhibition or broth test to determine the bactericidal activity of Col V. For zone of inhibition test, 3 × 10^8^ sensitive *E. coli* cells were first spread onto the LB agar plate supplemented with 50 μg mL^−1^ kanamycin. After centrifuging Col V-producing cultures at 16,000*g* for 25 min, 10 μL of supernatants containing antimicrobial peptides were spotted on the same plate and incubated the plate at 37 °C for 16 h. The diameter of each inhibition zone was then measured to assess bactericidal activity of Col V. For broth test, 50 μL of supernatant from the 21-h timepoint pelleted culture (centrifuged at 16,000*g* for 25 min) was added to the tube containing 2.25 × 10^6^ sensitive *E. coli* cells as well as 1.5 mL LB medium supplemented with 50 μg mL^−1^ kanamycin and then the tube was incubated at 37 °C for 16 h. The value of OD_600_ for each tube was measured after incubation to evaluate antimicrobial efficiency in each batch. The results represented the mean ± S.D. of three biological replicates.

### Betaxanthins production

The yeast integration cassette listed in Supplementary Table 1 was transformed into wild-type *S. cerevisiae* BY4741 resulting in a DOD enzyme-overexpressing sBY08 strain. To investigate the effect of medium composition and fermentation temperature on betaxanthins production with the use of a synthetic consortium, l-DOPA producer *E. coli* eBL0430D and DOD-expressing *S. cerevisiae* sBY08 were used in this study. The starter cultures of *E. coli* eBL0430D and *S. cerevisiae* sBY08 were cultivated into LB medium supplemented with 50 μg mL^−1^ kanamycin as well as 50 μg mL^−1^ spectinomycin and YSC + CSM-URA, respectively. The seeding *E. coli* and yeast cultures were then transferred to LBYSD or M9YSD medium (with initial OD_600_ of 0.05 for yeast and 0.0015 for *E. coli*) and incubated at various temperatures (25, 30, 33.5, 37, and 40 °C). For each timepoint, 100 μL of supernatant parts taken from 250 μL of pellet samples (with centrifugation at 2750*g* for 10 min) were transferred to a microtiter plate. Betaxanthins fluorescence was measured by the plate reader (BioTek) with reading gain 100, excitation at 482 nm and emission at 510 nm. The results represented the mean ± S.D. of three biological replicates. To evaluate if the reactor scale will affect the betaxanthin production, fermentations were conducted in 3 mL culture tube or 25 mL shaking flask containing M9YSD medium and incubated at 30 °C with initial OD_600_ of 0.05 for yeast and 0.015 for *E. coli*. 200 mg/L DOPA was also added to the medium to act as a positive control. Betaxanthins fluorescence was measured by the plate reader with reading gain 75, excitation at 482 nm and emission at 510 nm. To investigate the effects of aeration in culture tube and initial yeast cell number on betaxanthins production for the gel system, conditions including medium working volume 3 mL or 5 mL with 4.5 × 10^7^ or 4.5 × 10^6^ seeding yeast cells embedded in 0.3 g of hydrogels were examined in this study. 100 or 200 mg L^−1^ l-DOPA was added into the M9YSD medium for production testing. Samples were taken at 24-h intervals. Fluorescence was measured by the plate reader with reading gain 75, excitation at 482 nm and emission at 510 nm. To study the impact of fermentation temperature and initial cell density on betaxanthins production with the use of gel or free-cell system, various temperatures (25, 30, 33.5, 37, and 40 °C) as well as yeast: *E. coli* cell/gel ratios (100:1, 10:1, 1:1, 1:10, and 1:100 for liquid cultures system; 6:1, 3:1, 1:1, 1:3, and 1:6 for hydrogels) were adopted for production testing. For hydrogel system, each printed gel carried the same initial yeast or *E. coli* cell concentration 1.5 × 10^8^ cells/g of polymer. For free-cell system, all conditions included the same initial net yeast and *E. coli* cells concentration (3 × 10^6^ cells mL^−1^). An addition condition (1:1, 10 × ) mimicking the same number of initial cells used in the 1:1 gel ratio condition was employed in liquid culture system. 100 μL of supernatant taken from 120 μL of samples after centrifugation at 2750*g* for 10 min was transferred to a microtiter plate. Fluorescence was measured by the plate reader with reading gain 60, excitation at 482 nm and emission at 510 nm. To investigate the reusability of cell-laden living materials, five consecutive uses after round 0 were performed. The gels (6:1) and liquid culture samples (100:1 and 1:1 (10×)) with the highest betaxanthins titer at 30 and 33.5 °C were selected from the round 0 experiment and transferred to 3 mL M9YSD media for production testing. For liquid culture, repetitive uses were carried out through transferring 50 μL of consortia suspension from previous batch to the next batch. Samples were taken at 24-h intervals and each batch of reuse was performed for 3 days. Fluorescence was recorded by the plate reader with reading gain 60, excitation at 482 nm and emission at 510 nm. To evaluate the impact of preservation process on consortia activity in gel system, three different preservation methods including lyophilization, 4 °C storage and liquid nitrogen freezing were applied to preservation of consortia-laden polymers as described below. Additional five consecutive uses after preservation process were performed. Samples were taken at 24-h intervals and each batch of reuse was performed for 3 days. Betaxanthins fluorescence was recorded by the plate reader with gain setting of 60, excitation at 482 nm and emission at 510 nm. To evaluate the impact of three preservation processes on betaxanthins production between liquid culture and hydrogel system, 100 or 500 μL of pre-preserved consortia suspension was taken and individually proceeded to lyophilization, 4 °C storage and liquid nitrogen freezing. The spatially separated consortia hydrogels with 6:1 (yeast:*E.coli*) gel ratio were used in this study. The details of inoculum ratio of yeast to *E. coli* were described above. The preserved samples were then transferred to fresh media for the production and supernatants were taken at day 3. Fluorescence was recorded by the plate reader with reading gain 60, excitation at 482 nm and emission at 510 nm. For the long-term storage measurement, betaxanthins production was measured after 96 h of initial incubation to establish baseline production. After the hydrogels were then lyophilized. After 1 month of storage at room temperature, the hydrogels were resuspended in medium, and betaxanthin production was again measured after 96 h of incubation. To investigate the importance of spatial organization of consortia member in betaxanthins production, supernatants from 72-h fermentation cultures of separate hydrogel (l-DOPA producing bacteria-laden hydrogel was printed alongside a yeast-laden hydrogel) and mixed gel (two species were embedded in the same gel) were collected for betaxanthins measurement. Both separate and mixed gels (*n* = 9) carried the same initially inoculated cell numbers with 6:1 yeast-*E. coli* ratio. Fluorescence was recorded by the plate reader with reading gain 60, excitation at 482 nm and emission at 510 nm. To compare the F127-BUM hydrogel to caclium alginate hydrogels, a solution of 8 weight percent sodium alginate was prepared in water. This solution was then inoculated with 4.5 × 10^7^ cells for every 300 μL of alginate solution. This solution was then extruded into a bath of 3 wt% calcium chloride to induce physical crosslinking and to produce self-supporting calcium alginate hydrogels. 300 μL of bacteria and yeast calcium alginate hydrogels were then incubated in the fermentation media for 72 h at 30 °C. The resulting betaxanthins production was then compared to the 1:1 ratio of F127-BUM hydrogel.

### Determination of the percentage of leaked cells

To determine the degree of leaked *E. coli* and yeast cells in the hydrogel system, serial dilutions of the cultures incubated for 0.5 h from immobilized betaxanthins-producing consortia samples (with 6:1 yeast-*E.coli* gel ratio) were plated on YPD agar plate supplemented with 100 μg mL^−1^ of ampicillin to measure colony forming units (CFUs) of *S. cerevisiae* and solid LB supplemented with 10 μg mL^−1^ of nystatin (VWR Catalog No.: AAJ62486-06), an antifungal antibiotic, to measure CFUs of *E. coli*. To investigate the impact of washing process for consortia-laden hydrogels on the percentage of leaked cells between pre- and post-preservation, the CFUs of each specie for preserved gels with 0, 1, or 2 wash were normalized to that of pre-preserved samples with 0 wash. Gel samples for each treatment (*n* = 1) were incubated at 30 °C.

### Glucose/xylose utilization with repeated uses

To investigate the consortia activity on glucose/xylose utilization with repeated uses for gel and liquid culture systems, a parallel consortium *S. cerevisiae* wild-type S288C and xylose-utilizing YSX3 strains^36^ were used in this study. The yeast strains were precultured in YPD medium at 30 °C for 37 h. For hydrogel system, 4.5 × 10^7^ seeding cells for each yeast strain were individually immobilized in 0.3 g of hydrogel. For liquid culture system, 4.5 × 10^6^ seeding yeast cells for each strain were mixed and transferred to 3 mL medium. For round 0, two separate trials were carried out in parallel: one condition consisted of growing the yeast-yeast consortia in YPD media and the other grew the yeasts in YPDX media at 30 °C for 5 days to outgrow the population. Subsequently, three consecutive rounds of use were performed with all samples cultivated in YPDX media to test glucose and xylose consumption. Each re-use batch was carried out with 30 °C incubation for 96 h. For liquid cultures, repetitive uses were carried out through transferring 50 μL of consortia suspension from previous batch to the next batch. Samples were taken at 24-h intervals and the concentrations of residual glucose and xylose were analyzed by HPLC. The initial xylose consumption rate was determined at 24-h timepoint. The results represented the mean ± S.D. of three biological replicates.

### GC and HPLC analysis

For ethanol fermentation, samples from batch reactions were filtered through 0.2 μm nylon filters and subjected to gas chromatography (GC; Agilent 7890 A GC System) equipped with a Restek Rtx-BAC1 column (30 m, ID 0.53 mm, 3.0 μm) and oven program was 40 °C for 5 min. A 1 μL sample was injected at 253.07 mL min^−1^ of H2 flow. A peak for ethanol appeared between 1.85 and 1.95 min. Standard samples were prepared by adding known volume of pure ethanol into SC media (e.g., 50 μL of ethanol in 950 μL of SC media corresponds to 50,000 ppm v/v of ethanol) to obtain a calibration curve of concentration versus ethanol peak area in GC. For the rest of experiments, samples were filtered with 0.2-μm nylon syringe filters (Wheaton Science) prior to running HPLC. HPLC confirmation of tyrosine or l-DOPA production was performed using a Dionex UltiMate 3000 (Thermo Fisher Scientific) equipped with an Agilent Eclipse Plus C18 column (3.0 × 150 mm, 3.5 μm) with detection wavelength set to 280 nm. Column oven was held at 25 °C with 1% acetic acid in water or acetonitrile as the mobile phase over the course of the 20-min sequence under the following conditions: 5–15% organic (vol/vol) for 5 min, 15 to 100% organic (vol/vol) for 8 min, 100% organic (vol/vol) for 2 min, 100 to 5% organic for 2 min followed by 5% organic for 3 min. The constant flow rate was set at 0.8 mL min^−1^. A standard curve was prepared using 98.0% purity l-DOPA (3,4-dihydroxy-l-phenylalanine) from Sigma or 99% purity tyrosine (Acros Organics). For quantification of 2,3-BDO, an Aminex HPX-87H ion exclusion column (BioRad) was used. The flow rate using an isocratic mobile phase of 5 mM H_2_SO_4_ was set at 0.6 mL min^−1^ over 25 min. The column temperature was held at 60 °C and a refractive index detector (RID) (Kawaguchi) was used at 25 °C. A standard curve was prepared from 98% purity 2,3-butanediol (Sigma). For quantification of glucose and xylose concentration, an Aminex HPX-87P Column (BioRad) was used to measure samples. Filtered and degassed H2O was used as mobile phase with a flow rate of 0.6 mL min^−1^ over 38 min. The column was kept at 85 °C and a refractive index detector (RID) (Kawaguchi) was used at 25 °C. A standard curve was prepared using 99% purity glucose (MP Biomedicals) and xylose (Sigma). Data was analyzed using the Chromeleon 7.2 Chromatography Data System (Dionex).

### Preparation of tensile specimens

The 30 wt% F127-BUM hydrogels were brought below their *T*_gel_ and poured into ASTM D638 type V specimen molds. Each specimen was then brought back to 21 °C to induce gelation. Upon complete gelation, the specimens were cured for 30 min in a custom-fabricated curing chamber with sunlite 365 nm A19 UV Lamps. After photo curing, half of the samples were lyophilized. Prior to testing, all samples were stored in an excess of DI water for at least 24 h to reach equilibrium swelling.

### Mechanical tests

Tensile mechanical measurements were performed on as-prepared tensile specimens using an Instron 5585H load frame with a 50 N load cell and flat pneumatic grips. All tests were conducted at room temperature (22 °C) using a crosshead rate of 10 mm/min, until specimen failure. The dimensions of each specimen were measured with calipers prior to testing to ensure accurate calculation of stress and strain. At least five specimens of each formulation were tested. The Young’s moduli were calculated from the linear region of stress vs strain curve.

### SEM analysis of material structure

Prior to imaging, the hydrogel samples were dipped into liquid nitrogen and lyophilized overnight. One sample was imaged before on-demand production of betaxanthins, while the second sample was subjected to two consecutive, on-demand fermentation cycles before imaging. All SEM images were captured using a ThermoScientific Apreo-S scanning electron microscope.

### General scheme for preservation and re-use cycles

Prior to preservation process, all gel samples were taken from the gel cultures at the end of fermentation and washed 1–2 times with 400–800 μL of fresh medium. The cell-laden materials were then transferred to a new sterile culture tube. For the lyophilization process, the culture tubes carrying hydrogels were first placed into a liquid nitrogen tank for 10–15 min. After the freezing step, the tubes containing frozen gels were placed into a dryer chamber Supplementary Fig. 9a; right). During the drying process, the moisture was withdrawn from the polymers through sublimation under vacuum. Vacuum was applied to the dryer chamber overnight to ensure the water was completely removed from the gels. For refrigerated storage treatment, the culture tube carrying *E. coli*-yeast gels was stored in the refrigerator (set to 4 °C) for a week. For liquid nitrogen freezing process, the culture tubes containing hydrogels were first placed into a liquid nitrogen tank for 10-15 min. Subsequently, the frozen tubes were stored at −80 °C for 16 h. After preservation process, the preserved gels were then transferred to fresh medium to proceed with continuous re-use.

### Statistical analysis

For comparing pre- and post-lyophilized samples for 2,3-BDO and l-DOPA production, statistical analysis was performed using Wilcoxon matched-pairs signed-rank test in GraphPad Prism 6 software. Data were considered statistically significant when *p* < 0.05 by paired *t* tests. For comparing betaxanthins production between separate and mixed hydrogel samples, statistical analysis for the means of two groups was performed using unpaired *t* test with Welch’s correction in GraphPad Prism 6. Statistical analysis for the standard deviations of two groups was performed using the Levene’s test in Excel. Data are presented as the mean ± S.D. from at least three biological replicates, unless stated otherwise in Figure legends and experimental methods.

### Reporting summary

Further information on research design is available in the Nature Research Reporting Summary linked to this article.

## Supplementary information

Supplementary Information

Reporting Summary

## Data Availability

Data that support the findings of this study and the plasmids and strains used are available from the corresponding authors upon request. Source data are provided in the Source Data file.

## Source data

Source Data
