# Supplementary material for: Compartmentalized microbes and co-cultures in hydrogels for on-demand bioproduction and preservation
Source: Nat Commun. 2020 Feb 4;11:563. doi: 10.1038/s41467-020-14371-4 (PMC7000784; doi:10.1038/s41467-020-14371-4)
Supplement: Supplementary file 1 — Supplementary Information [file 41467_2020_14371_MOESM1_ESM.pdf]

Supplementary Information for

Compartmentalized microbes and co-cultures in hydrogels for on-demand bioproduction and preservation

Johnston *et al*.

Supplementary Note

2,3-butanediol production in yeast with lyophilization and re-use

2,3-Butanediol is a building block biochemical for a variety of downstream chemicals including methyl ethyl ketone, acetoin and 1,3-butadiene with applications in synthesizing plastics, food additives or industrial solvents [7]. 2,3-BDO has a global market demand of around 32 million tons per year with a value of $43 billion [8]. As a result, microbial 2,3-butanediol production has been extensively studied in the past few decades [7]. Previously, we have demonstrated that a multiplexed dCas9-based regulation system to simultaneously knock down the NADH sinks including *ADH1*/*3*/*5* and *GPD1* along with overexpression of endogenous NADH-dependent BDH1 enzyme can increase the production of 2,3-BDO by nearly 2-fold in a 2,3-BDO-producing *S. cerevisiae* CEN.PK2-a strain (harboring a heterologous pathway with overproducing NoxE, alsD and alsS enzymes) [4] (Fig. 2a).

To evaluate the efficacy of the microbe-laden hydrogel system, two 2,3-BDO-producing yeast strains (BY4741 and CEN.PK2-a) were each encapsulated in the gel matrix and performance was compared pre- and post-preservation (Supplementary Fig. 1). The 2,3-BDO productivity between the two engineered strains of BY4741 (0.035 g/L/h) and CEN.PK2-a (0.034 g/L/h) were comparable at 48 hours before lyophilization. After lyophilization, the 2,3-BDO productivity in preserved BY4741 gel (0.03 g/L/h) retained nearly 90% efficiency compared to the untreated sample, while the biological productivity in lyophilized CEN.PK2-a hydrogels decreased to 0.025 g/L/h (about 70% efficiency). Part of the decrease in efficiency seen with 2,3-BDO in the CEN.PK2-a strain was a result of an increased acetoin byproduct titer seen in this condition that increased from 1.1 g/L (pre-lyophilization) to 1.8 g/L (post-preservation) at the 48-hour timepoint (Supplementary Fig. 1 B). Acetoin is formed from the bioconversion of 2,3-BDO into acetoin via

reversible yeast butanediol dehydrogenase (BDH1) enzymatic reactions [9]. Regardless, the net metabolic activity (acetoin + 2,3-BDO) evaluated for both pre- and post-lyophylization is indicative of sustained metabolic activity by the yeast-laden gels.

L-DOPA production in *E. coli*

L-DOPA can be biosynthesized from tyrosine and is a precursor of the neurotransmitter dopamine in human [10] and has also been used as a drug for treating Parkinson's disease [11]. As a result, microbial production of L-DOPA [12, 13] from low-cost lignocellulosic feedstocks [14, 15] is an attractive process compared to direct extraction from plants restricted by the low yield and purity issues [16].

Previous studies have shown that introduction of feedback-inhibition-resistant 3-deoxy-d-arabinoheptulosonate-7-phosphate (DAHP) synthase (encoded by $aroG^{fbr}$) and chorismate mutase/prephenate dehydrogenase (encoded by $tyrA^{fbr}$) in a *tyrR* (encodes a transcriptional regulator of aromatic amino-acid biosynthesis) knockout *E. coli* can sufficiently overproduce tyrosine [17, 18]. In order to redirect metabolic flow from the intracellular tyrosine pool to the L-DOPA biosynthesis pathway, 4-Hydroxyphenylacetate 3-hydroxylase (HpaBC) [13] was overexpressed in the tyrosine-producing strain eBL04 (Fig. 2b). As expected, expression of *hpaBC* under the control of a constitutive promoter with a strong synthetic ribosomal binding site resulted in *E. coli* eBL0430D exhibiting a higher titer of L-DOPA (around 213 mg/L) than that of the eBL0432D strain that has a weaker overall expression (about 160 mg/L) (Supplementary Fig. 2). Additionally, a change in coloration of culture broth for both engineered strains was observed, indicative of the oxidation of L-DOPA to form brown melanin-like pigments [13, 19, 20].

Peptide antibiotic production in *E. coli* with lyophilization and re-use

To expand beyond small molecules, we demonstrate that microbe-laden hydrogels are capable of producing small peptides—in this case, biosynthesis of peptide antibiotic colicin V (colV). ColV is secreted by *E. coli* and other members of *Enterobateriaceae* and previous studies have identified that four genes *cvaA*, *cvaB*, *cvaC* and *cvi* of the colicin V gene cluster are required for ColV synthesis, export, and native immunity [5, 21, 22] (Supplementary Fig. 4 A). The colicin V-producing strain expressing its cognate immunity protein Cvi alone is sufficient to protect itself from the killing activity of ColV [5]. The extracellular secretion of ColV, encoded by the *cvaC* gene, is mediated by an efflux system containing cytoplasmic proteins CvaA and CvaB as well as the host outer membrane protein TolC [21], then the CvaA-CvaB-TolC exporter recognizes the N-terminal 15-amino-acid leader sequence of primary translation polypeptide ColV and proteolytically processes it to render a mature 88-amino-acid ColV peptide. The secreted ColV is only active against sensitive cells when inserted into the inner membrane of a target cell resulting in membrane depolarization [5, 22].

In this study, ColV antibiotic-producing *E. coli* that was expressing *cvaA*, *cvaB*, *cvaC* and *cvi* genes was encapsulated in the F127-BUM hydrogel matrix. Using the printed gels, two bactericidal assays were conducted using the broth supernatant of the culture. These assays included a zone of inhibition test and bacterial suspension broth test to evaluate the antimicrobial activity of ColV. As shown in Figure 2d, the inhibition halo yielded by the gel system was consistently observed over four consecutive repeated uses after lyophilization, indicating the cell functionality in the gels was still retained and the diffusion of the peptide from the gel matrix was not inhibited. The bactericidal activity was not affected by the preservation process for the first two repetitive uses and the efficiency was reduced to only around 80% for the last batch of re-use

compared to the original (round 0). The average antimicrobial activity for four subsequent rounds of re-use after lyophilization was nearly 100% of the original gel based on the measurement of diameter of clear zones appeared on agar plates. In addition, the production of the secretory peptide antibiotic in gels was observed in the broth test (Supplementary Fig. 4 C) despite the slight reduction in antimicrobial functionality based on the inhibition zone test for the last two rounds of re-use (Supplementary Fig. 4 B). Thus, the overall production was maintained across the rounds of re-use using a mixture of bacteriastatic and bactericidal assays.

For benchmarking, the bactericidal activity of the liquid culture system was also compared to that in the hydrogel system (Supplementary Fig. 4 B and C). The zone of inhibition test (Supplementary Fig. 4 B) and broth test (Supplementary Fig. 4 C) clearly demonstrated that the antimicrobial peptide activity made in a free-cell system was inconsistent for iterative cell-re-use compared to the hydrogel system. In the 3rd re-culturing round, the inhibition zone in free-cell system was actually not observed and its bactericidal activity dramatically was reduced to 6.5%. Likewise, the larger error bars on the liquid culture demonstrates the extreme variability of samples from biological replicates indicative of the instability experienced in repeated liquid subculturings.

Overall, these results showcase the capability of the described hydrogel system in maintaining high cell density and supporting protection to lyophilization, thus resulting in a continuous operation of peptide antibiotic production. Moreover, it is expected that the printed gel can carry a higher next cell density/concentration than a microbial suspension system [23, 24], thus leading to a stronger and more consistent production level.

Betaxanthins production via a synthetic commensal consortium

Betaxanthins are water-soluble natural yellow pigments that have been proposed as a substitute for artificial yellow dyes [25]. Previous studies have established a *de novo* plant betaxanthins biosynthesis pathway in *S. cerevisiae* for high-throughput screening of tyrosine hydroxylase mutant library and for the investigation of spectral and physical properties of various amine-betaxanthins [26, 27]. Given these potential uses and interest, we decided to choose the compound as an example molecule that can be produced via a microbial consortium. In this study, we created a synthetic *E. coli*-yeast consortium (Fig. 3b) whereby the L-DOPA secreted by engineered *E. coli* is consumed by the DOPA-4,5-dioxygenase (DOD)-overproducing yeast that converts L-DOPA into betalamic acid. The free betalamic acids then condense with primary or secondary amines to yield the fluorescent yellow betaxanthin pigments.

To evaluate the impact of medium components on fermentative performance, we evaluated free cell, suspension cultures using *E. coli* eBL0430D-*S. cerevisiae* sBY08 were tested at different temperatures (Supplementary Fig. 7 A). Betaxanthins titers gradually increased over time at 25 °C for both LBYSD and M9YSD conditions. Although the maximum titer of betaxanthins for each condition was nearly 2-fold higher when using LBYSD medium compared with using M9YSD, the production significantly declined over time for higher temperatures 33.5 to 40 °C, suggesting using M9YSD medium could provide a more stable condition for the product. Thus, M9YSD medium was chosen for the following studies. We found a consistent correspondence of betaxanthins titer between fermentation conducted in flask scale and tube scale (Supplementary Fig. 7 B); therefore, we chose to conduct experiments at the tube scale for operational convenience and condition multiplexing. Previous studies have demonstrated DOD reaction requires oxygen [26]. To verify the effect of aeration for test tube culture on betaxanthins production in the gel system,

the DOD-yeast-laden hydrogel was printed and cultured in a working volume of 3 mL or 5 mL medium supplemented with exogenous L-DOPA (Supplementary Fig. 7 C). Increasing the concentration of spiked L-DOPA or encapsulated cell biomass in gels enhanced betaxanthins production. Fermentation performed with 3 mL working volume led to a 1.2-fold increase in production compared to that with 5 mL medium, potentially due to the increased oxygenation; therefore, a 3 mL working volume was adopted for the subsequent experiments.

To further explore the impact of fermentation temperature and initial cells density on betaxanthins production, we evaluated varying temperatures and cell ratios for both liquid culture and gel systems (Fig. 3c and Supplementary Fig. 7 D-L). To control gel ratios, various amount of gel was printed and cured. To control liquid culture ratios, different amounts of starting culture was re-suspended. In this study, each printed gel carried the same initial yeast or *E. coli* cell concentration of 1.5 x 10^8 cells per gram of polymer. In liquid culture, we altered the initial cell ratio ranging from 100:1 to 1:100 while keeping the initial net yeast and *E. coli* cell concentration constant at 3 x 10^6 cells per mL of culture. As shown in Figure 3c and Supplementary Fig. 7 D-L, the production using gel system surpassed the microbial suspension culture system for most conditions. The consortia productivity in gel system with a gel ratio of 1:6 outcompeted the liquid culture with cell number ratio 1:100 by nearly 2-fold, especially across a range of temperatures (25-33.5°C). For 1:1 ratio at 25 °C, the gel system produced more betaxanthins compared to liquid system. At this condition, despite the starting point containing equal amount of *E. coli* and yeast cells, the consortia composition in suspension culture could be biased towards having more yeast than bacteria since 25 °C is closest to the yeast optimal growth temperature. In contrast, the gel based system can better control the end-dynamics of the consortium which is important for improving production [23].

Next, we evaluated the reusability of the consortium-laden gels (Fig. 3d and Supplementary Fig. 8 A-E). The activity throughout five consecutive rounds of re-use was determined by comparing the performance of gels with 6:1 ratio (as measured by the comparing the highest betaxanthins titer measured at 30 and 33.5 °C to that of the round 0 experiment) (Supplementary Fig. 7 L; top) to that using the liquids with 100:1 and 1:1 (10X) (the condition mimicking the same amount of initial cell number used in the 1:1 gel ratio condition) ratios (Supplementary Fig. 7 L; bottom). In this study, the gels retained their active metabolism and continuously produced betaxanthins and the titer substantially out-competed that of the free-cell fermentation system for both the 30 and 33.5 °C conditions (Supplementary Fig. 8 C-E). Importantly, the metabolic activity of the immobilized consortium was retained and exceeded 100% activity when compared with the original gel (round 0) for five continuous re-use at 30 °C (Supplementary Fig. 8 D). The consortia activity of gel at 33.5 °C was 100% and 95% of the original activity for the first three rounds and last two rounds of re-use, respectively (Supplementary Fig. 8 E). Although betaxanthins production for the round 0 took longer than that of a free-cell system (Supplementary Fig. 7 D-G), once the steady state of both embedded species was established, the gels rapidly produced their maximum biocatalytic rate, namely, a higher productivity was observed, for the next re-use rounds (Supplementary Fig. 8 A-C).

In contrast, the free-cell system at 30 °C performed a in a fluctuating manner and did not yield consistent betaxanthins titer for the five consecutive re-culturings (Supplementary Fig. 8 D). In fact, two of the biological triplicates could not make the betaxanthins after subculturing, suggesting that liquid culture system is unstable and subject to random consortia dynamics. Additionally, the free-cell system at 33.5 °C rapidly lost all ability to make betaxanthins (Supplementary Fig. 8 E). Moreover, the cell pellets of the 100:1 liquid samples at 96 hour fermentation timepoint at 33.5 °C

for the first round of cell-re-use displayed brown pigments (Supplementary Fig. 8 B), likely due to the overabundance of *E. coli* and the formation of melanin-like by-product yielded from oxidation of L-DOPA [13]. In addition, the pelleted cells showed no color change for 1:1 (10X) samples at the same condition. These results suggest the end consortia composition for continuous cell use experiment was varying through liquid culture. It should be noted that the titer obtained from the gel system may also be underestimated, due to some yellow pigmentation observed as being retained in the gel after fermentation.

Maintaining cell viability is paramount to industrial-scale bioprocesses [24]. Three different treatments for preservation of this consortia were conducted including lyophilization, refrigerated storage and liquid nitrogen freezing (Fig. 3d and Supplementary Fig. 9 A and B). The lyophilization process resulted in no loss of consortia productivity over five subsequent repeated fermentations after the fifth round of initial re-use (Fig. 3d). The biocatalytic efficiency at 30 °C was still retained at nearly 120% compared to the round 0 experiment. In addition, the gel after refrigerated storage treatment for one week still exhibited average consortia productivity at around 140% of the original for the last five rounds of re-use carried out at 30 °C (Fig. 3d). The control of cooling rate as well as the use of cryoprotectants have been demonstrated to improve cell viability and long-term stability [24]. However, we demonstrate here that this hydrogel ink is capable of providing protection from these conditions. To further demonstrate this, we simply employed liquid nitrogen to freeze the gels that were selected from the round 5 experiment at 33.5 °C, and proceeded to store the frozen gels at -80 °C overnight. The consecutive re-use study even on these gels showed that the cell viability was not severely affected by these freezing conditions, a condition that usually causes mechanical damage of cell membranes due to the formation of large ice crystals or irreversible protein denaturation [24]. Specifically, the average metabolic activity of

entrapped consortia reduced to only nearly 85% of original for the last five repeated batches (Supplementary Fig. 9 B).

Xylose/Glucose utilization via a yeast-yeast consortium with repeated gel-re-use

Xylose is the most abundant components in hemicellulose of lignocellulosic feedstock [28], however, conventional wild-type yeast *S. cerevisiae* is not capable of assimilating xylose [29, 30]. Previous studies have demonstrated a number of methods to import this metabolism including heterologous overexpression of *Scheffersomyces stipitis* xylose reductase (XYL1), xylitol dehydrogenase (XYL2) and D-xylulokinase (XYL3) in *S. cerevisiae* resulting in a xylose-utilizing strain YSX3 improved cell growth and ethanol production from xylose [6].

We sought to use our hydrogel system to enable a yeast-yeast parallel consortium. Wild-type *S. cerevisiae* S288C along with an engineered xylose-utilizing yeast, YSX3, were each individually encapsulated in hydrogels and the glucose/xylose consumption efficiency was compared with the liquid culture system (Fig. 3e and Supplementary Fig. 11). The sugars utilization rate was not determined for the round 0 since the purpose of this round was to outgrow and establish the consortia population. Two separate experiments were performed in parallel for the round 0: one with the yeast-yeast co-culture in YPD medium containing only 20 g/L of glucose (Supplementary Fig. 11 A), and the other in YPDX medium comprising 15 g/L of xylose and 15 g/L of glucose (Fig. 3e). In all cases of the re-use (for both gels and suspension systems), glucose was fully used within 24 hours and the systems exhibited a diauxic shift [30]. As shown in Supplementary Fig. 11 A, the gels initially maintained in YPD medium for round 0 displayed nearly 3.5-fold improvement on the average of initial xylose consumption rate over the liquid cultures during three subsequent rounds of re-use. Additionally, a lag phase in xylose consumption

rate for the first round of re-use in both systems was observed (Supplementary Fig. 11 C; left), due to the shift in metabolism [30].Furthermore, the performance of liquid culture progressively declined over each round of the re-use (Fig. 3e) and the xylose consumption rate for the round 3 (around 0.1 g/L/h) approached that seen in Supplementary Fig. 11A (about 0.07 g/L/h), indicating the free-cell system could not properly maintain the dynamics of the consortium. In contrast, our consortia-laden hydrogels exhibited a higher xylose consumption rate (0.3 g/L/h on average) compared to the liquid culture system over each round of repetitive use with the increasing improvement on xylose utilization (1.5-fold increase in round 1, 1.8-fold increase in round 2, and 2.7-fold improvement in round 3) (Fig. 3e).

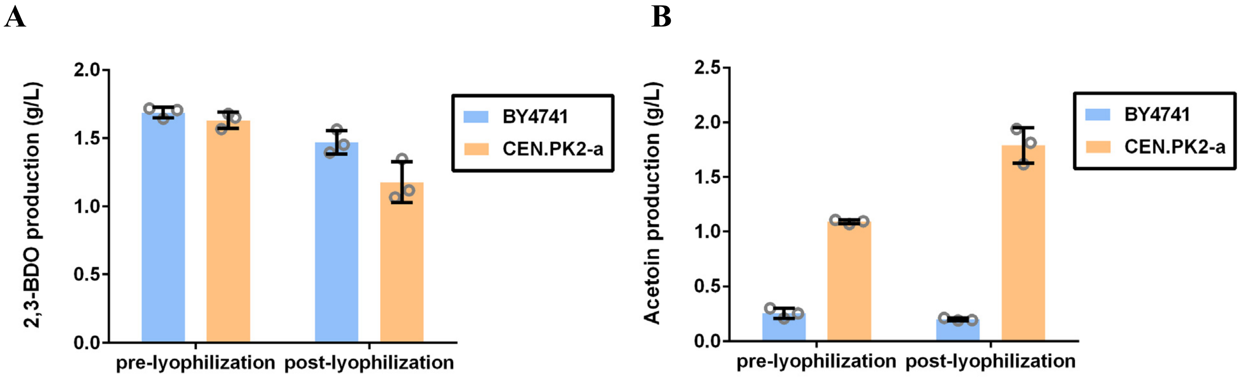

Supplementary Fig. 1. Gel-re-run for 2,3-butanediol (BDO) production. A, The comparison of 2,3-BDO production between *S. cerevisiae* BY4741 and CEN.PK2-a strains before and after lyophilization. **B,** The comparison of by-product acetoin formation between BY4741 and CEN.PK2-a strains before and after lyophilization. Each data point and error bar represent means and standard deviations from biological triplicates, respectively. Source data are provided as a Source Data file.

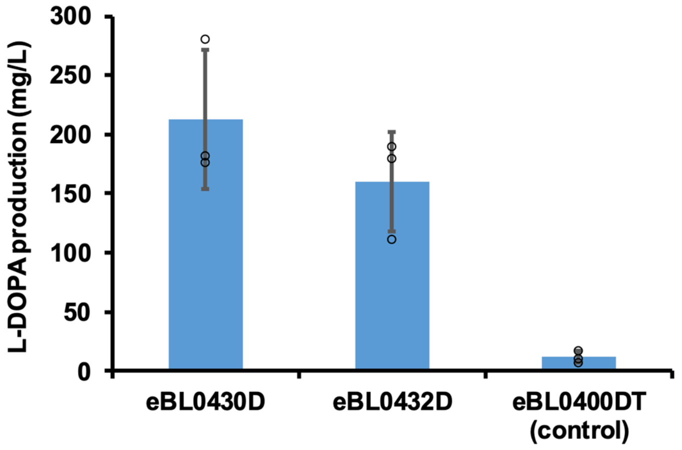

Supplementary Fig. 2. L-DOPA production in *E. coli*. The comparison of L-DOPA production between engineered *E. coli* strains and control strain eBL0400DT. The highest L-DOPA producer eBL0430D strain was used for DOPA and betaxanthins experiments. Each data point and error bar represent means and standard deviations from biological triplicates, respectively. Source data are provided as a Source Data file.

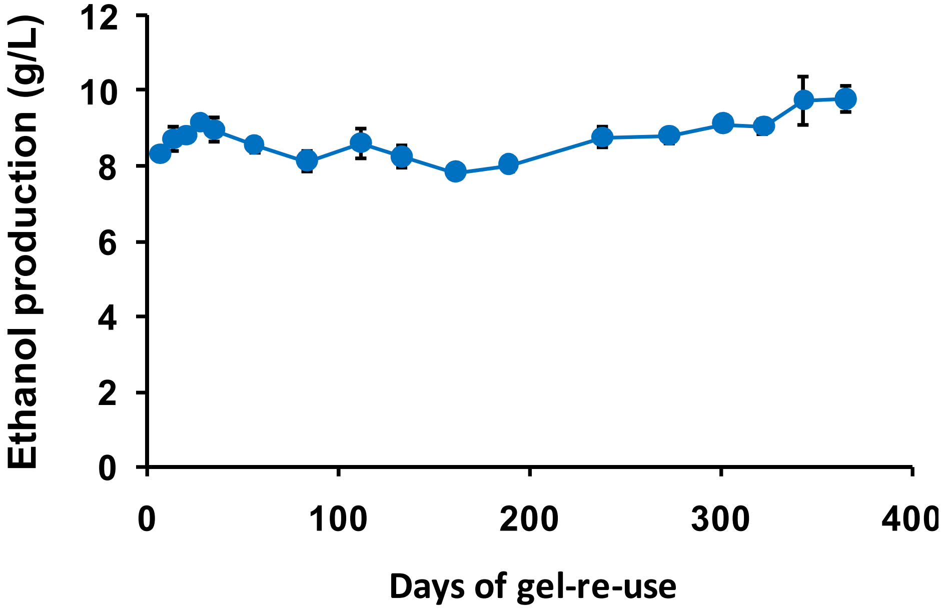

Supplementary Fig. 3. The reusability of yeast-laden hydrogels for a year-long ethanol fermentation. Ethanol titer (g/L) in yeast-embedded polymers are measured for each round of reuse. Each data point and error bar represent means and standard deviations from four biological replicates. Source data are provided as a Source Data file.

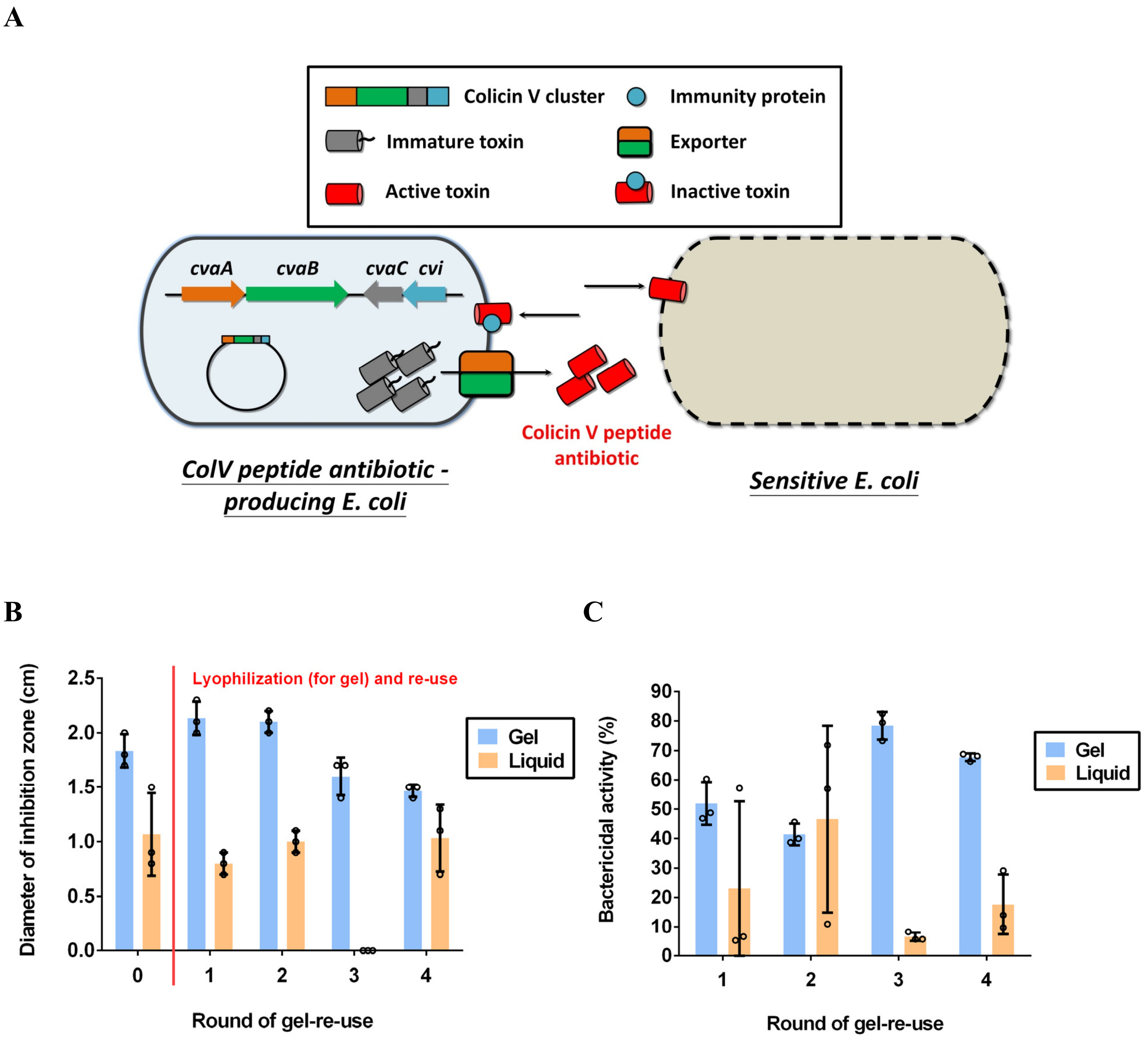

Supplementary Fig. 4. Gel-re-run for colicin V (ColV) peptide antibiotic production. A, The bactericidal mechanism of ColV. Zone of inhibition test (**B**) and broth test (**C**) are used for evaluating antimicrobial activity of ColV in gel system and liquid culture before and after lyophilization. The inconsistency in toxin production by liquid culture suggested that liquid culture might be unstable and subject to random dynamics. Each data point and error bar represent means and standard deviations from biological triplicates, respectively. Source data are provided as a Source Data file.

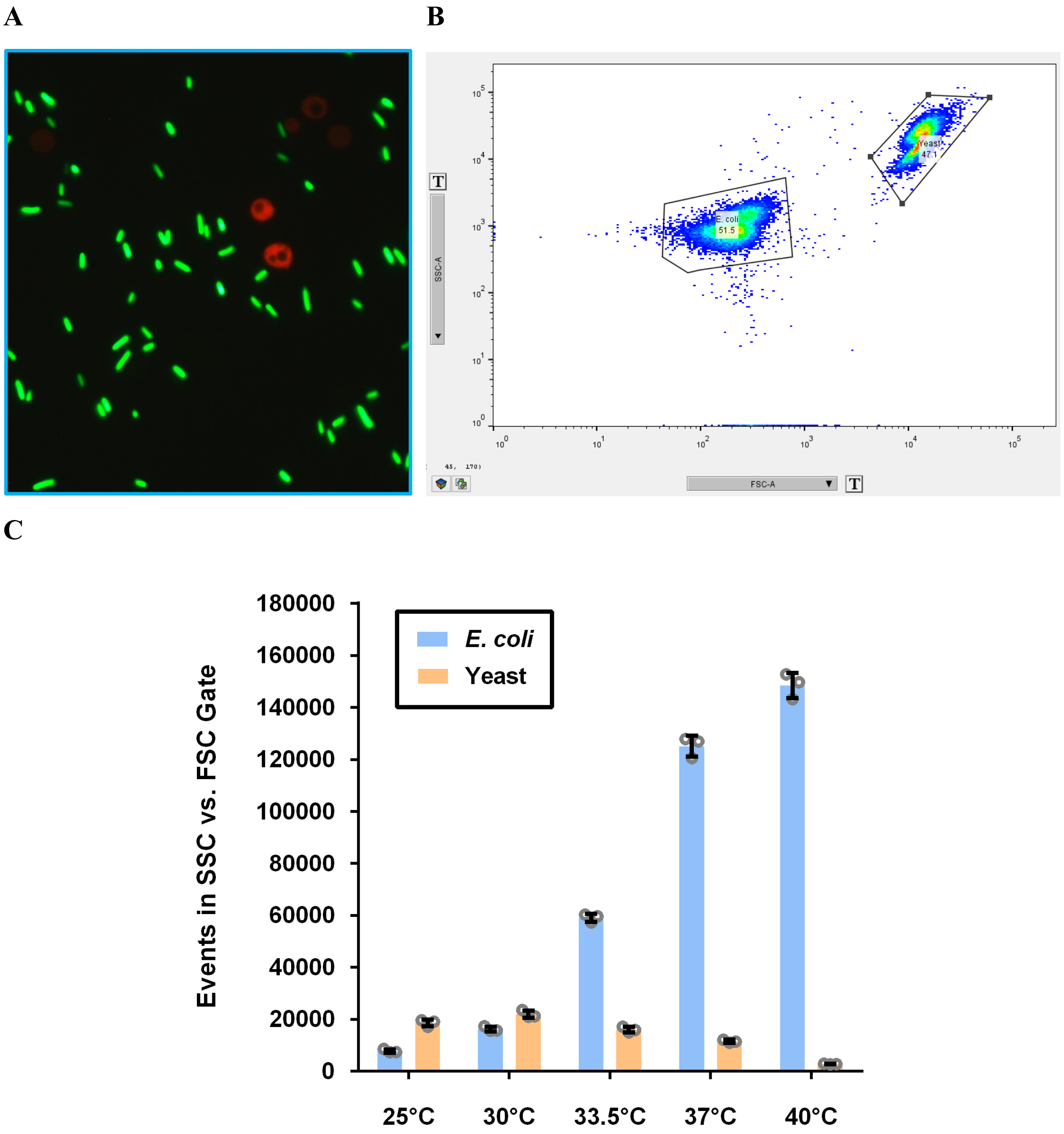

Supplementary Fig. 5. Co-culture flow cytometry. A, Visual confirmation of fluorescence and mixed culture under fluorescence microscope. **B,** SSC versus FSC plot for distinguishing and counting *E. coli* and yeast using BD Fortessa flow cytometry. **C,** Impact on consortia dynamics for bulk culture with different temperatures. Each data point and error bar represent means and

standard deviations from biological triplicates, respectively. Source data are provided as a Source Data file.

A

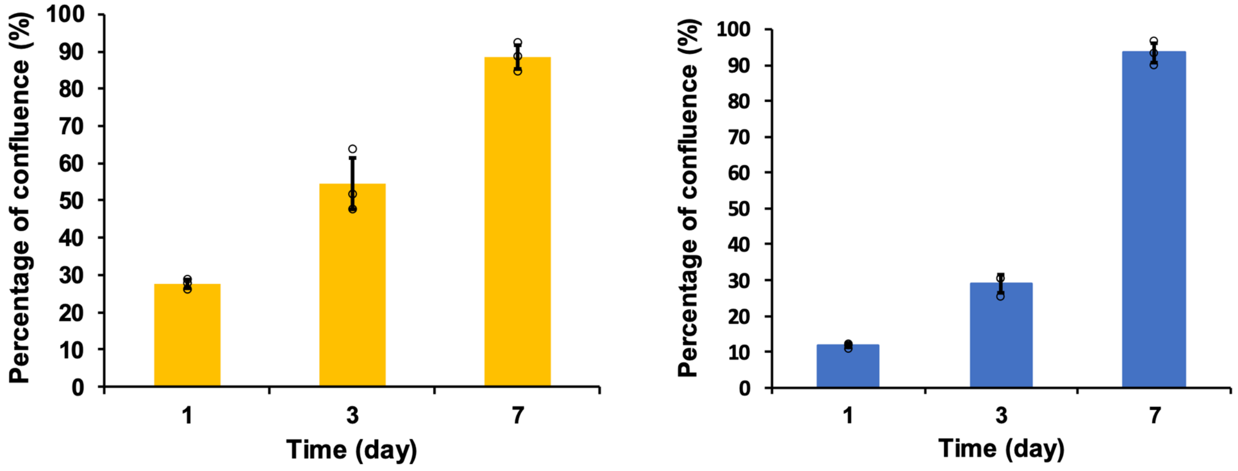

B

Day 7 Standards

30 °C – Yeast in SC

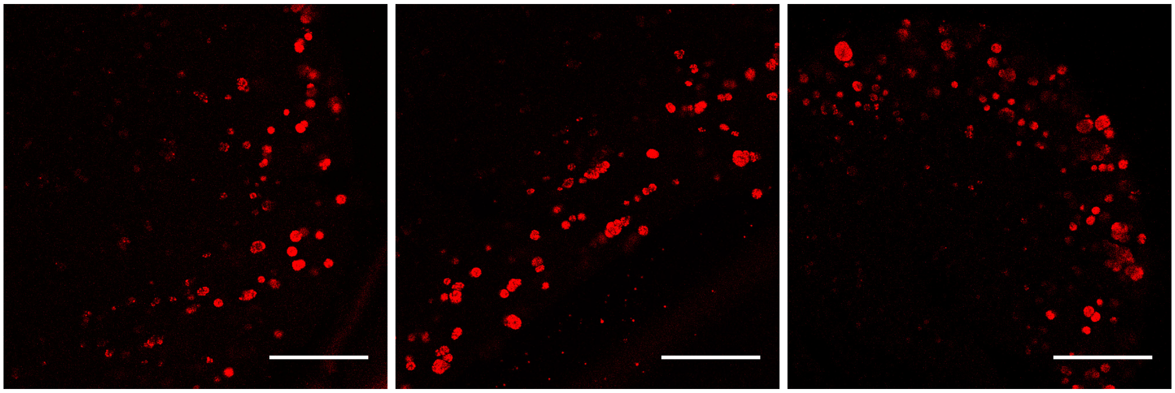

37 °C – *E. coli* in LB

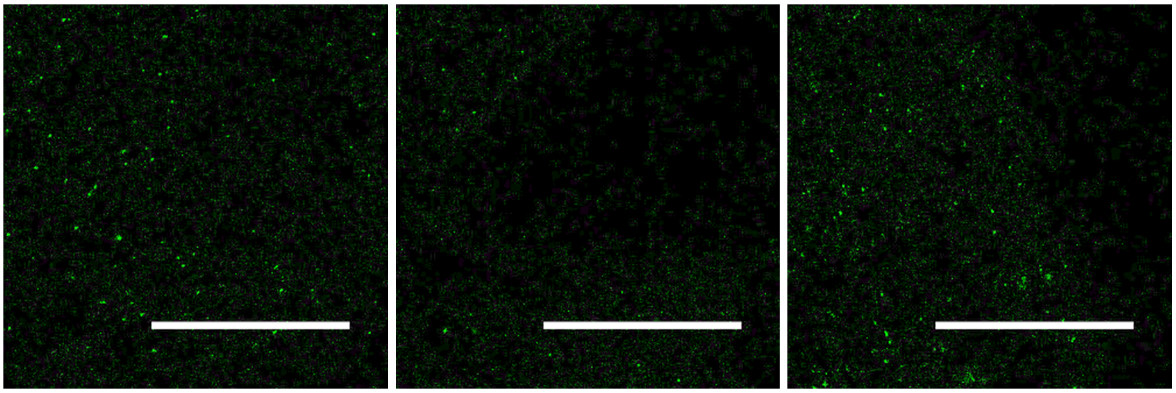

C

Day 1

25 °C – Yeast

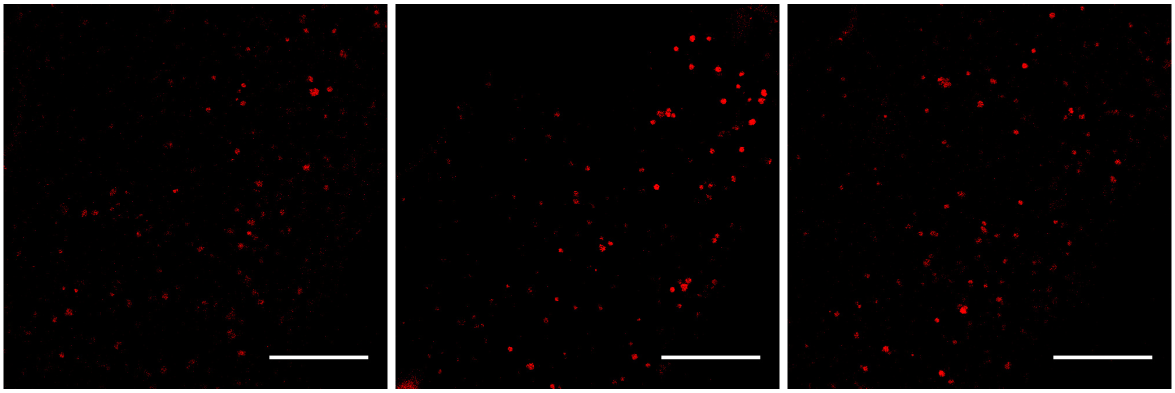

25 °C – *E. coli*

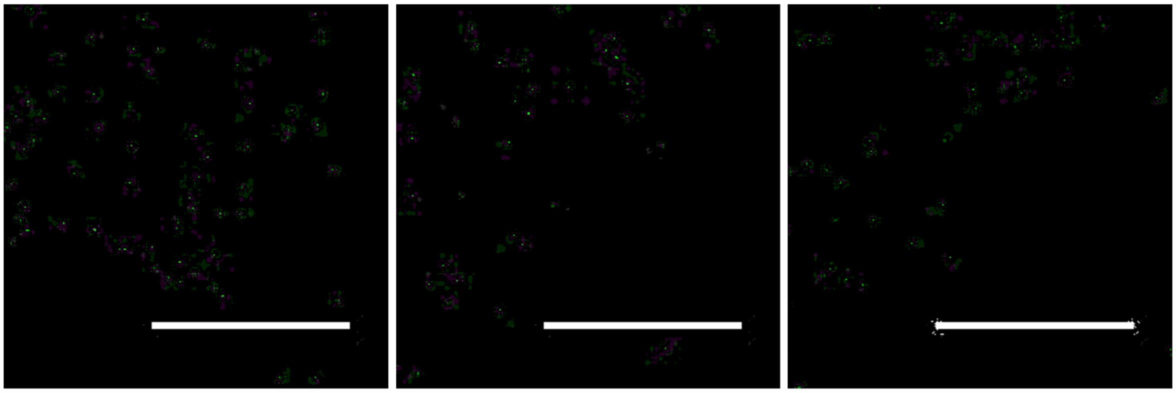

Day 3

25 °C – Yeast

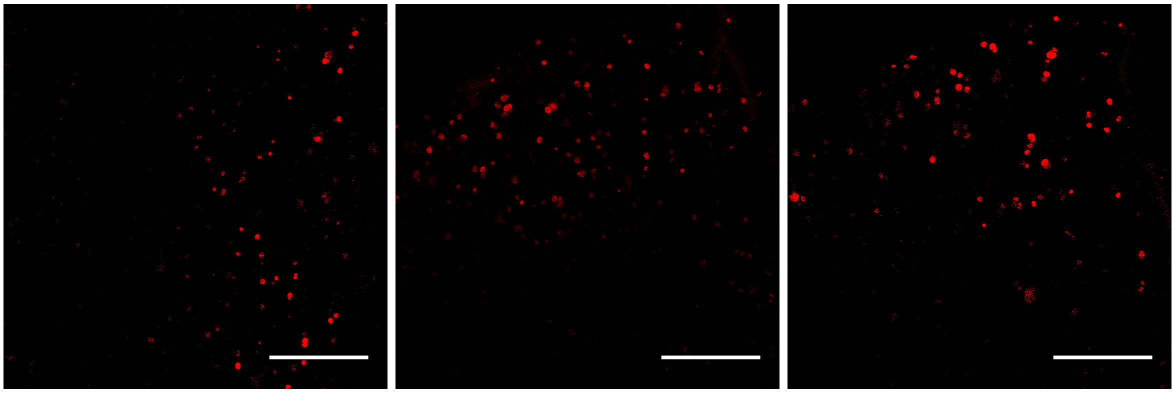

25 °C – *E. coli*

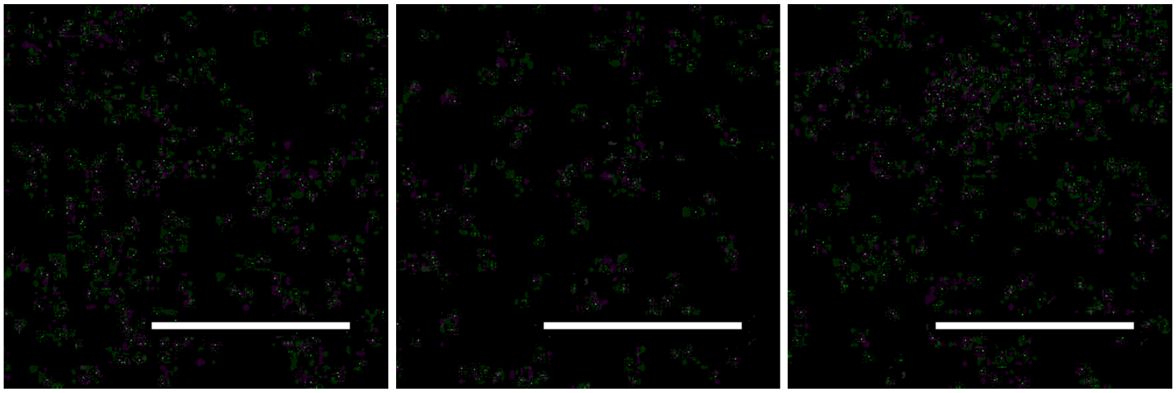

Day 7

25 °C – Yeast

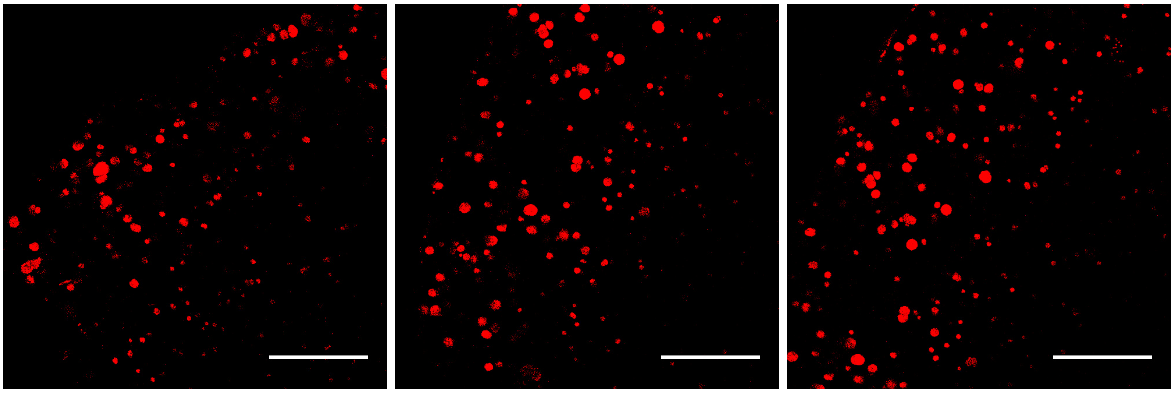

25 °C – *E. coli*

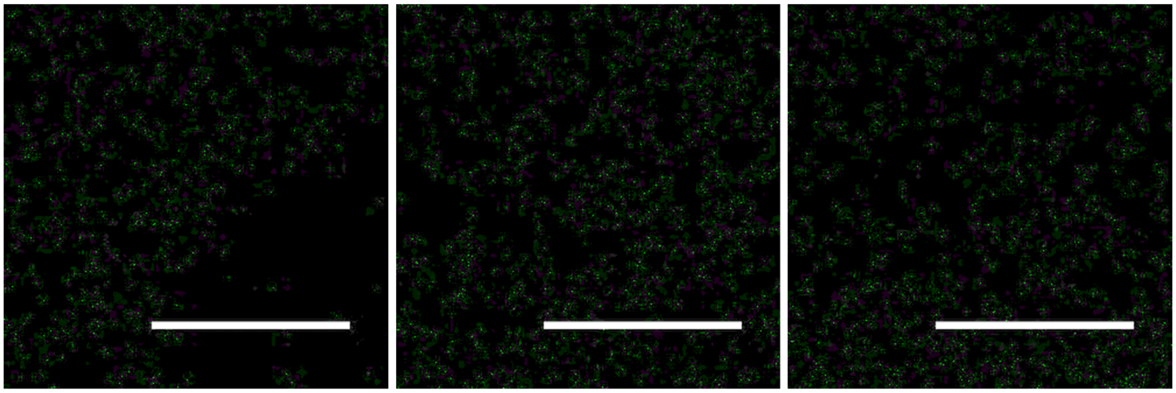

Day 1

30 °C – Yeast

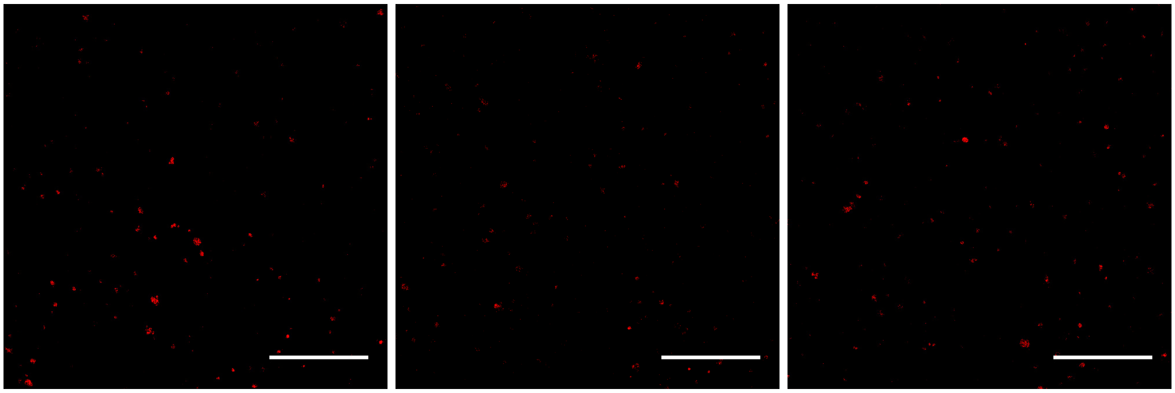

30 °C – *E. coli*

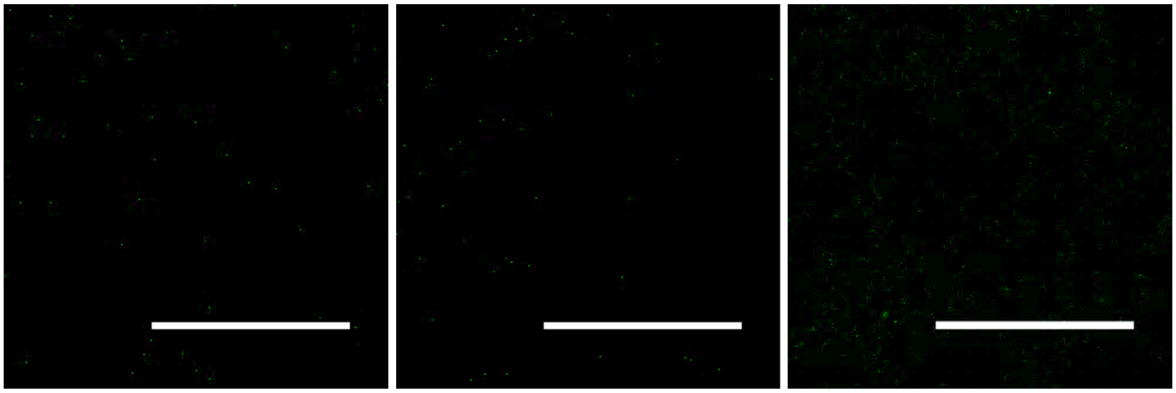

Day 3

30 °C – Yeast

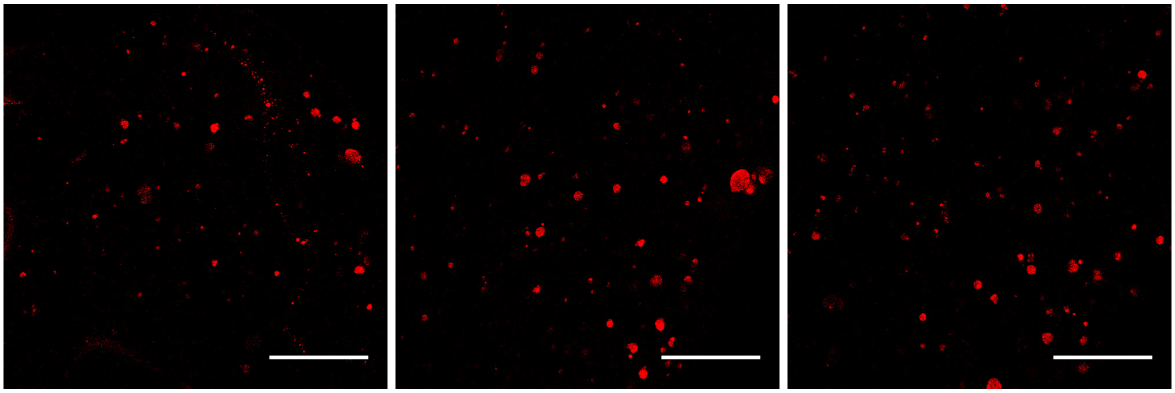

30 °C – *E. coli*

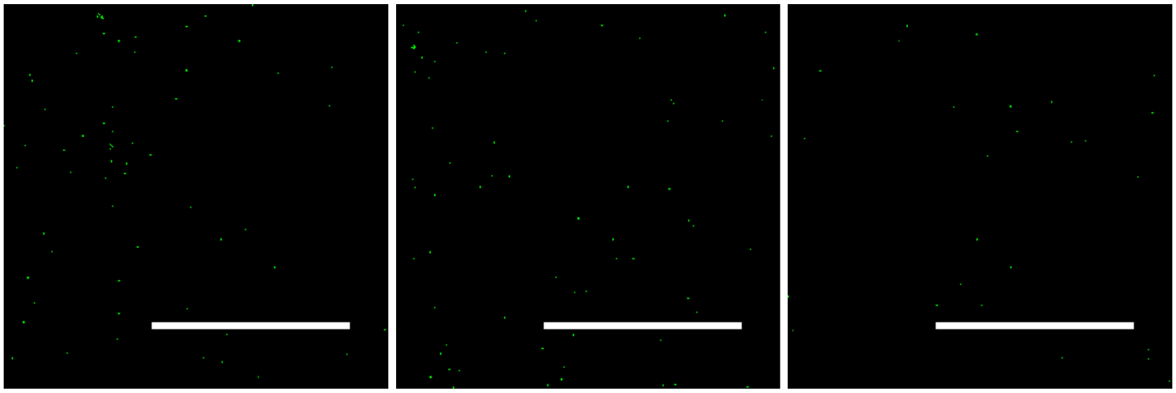

Day 7

30 °C – Yeast

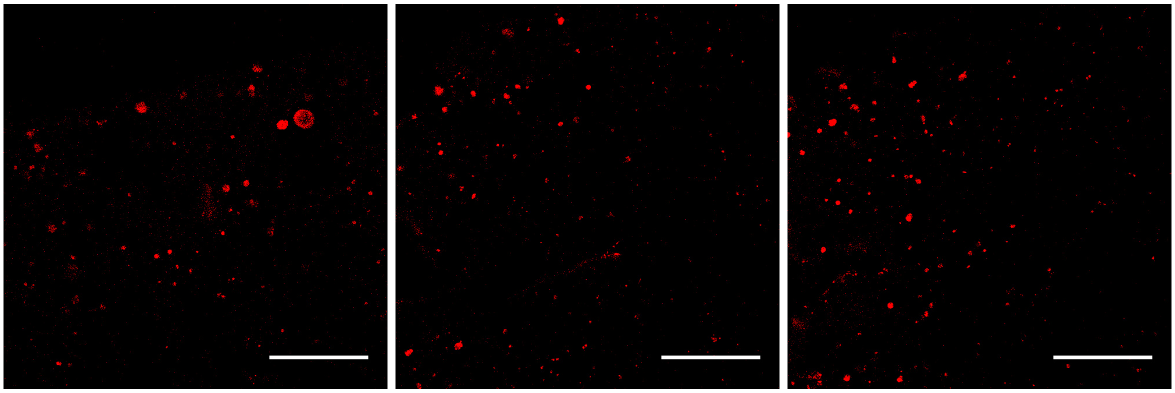

30 °C – *E. coli*

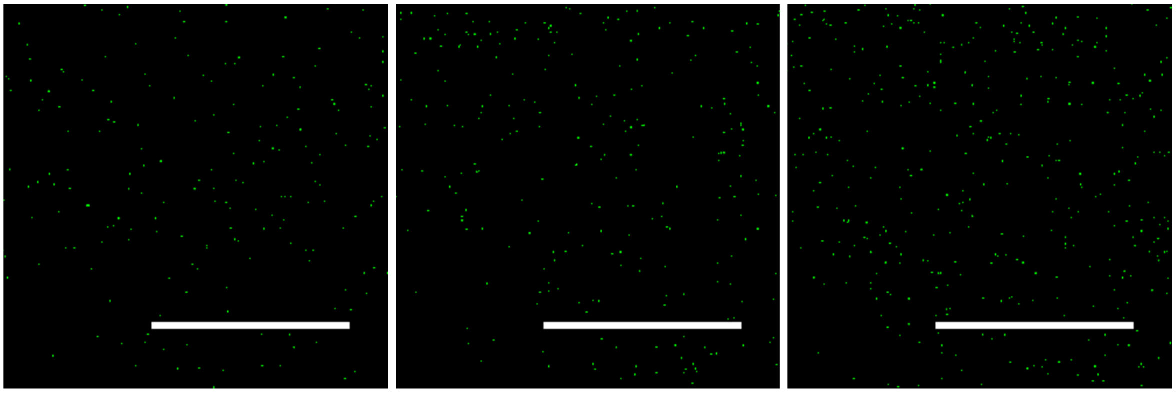

Day 1

33.5 °C – Yeast

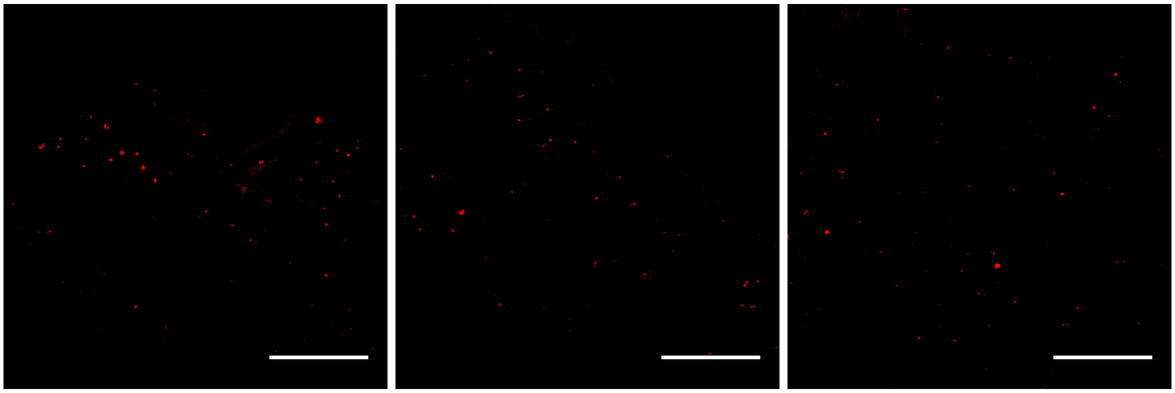

33.5 °C – *E. coli*

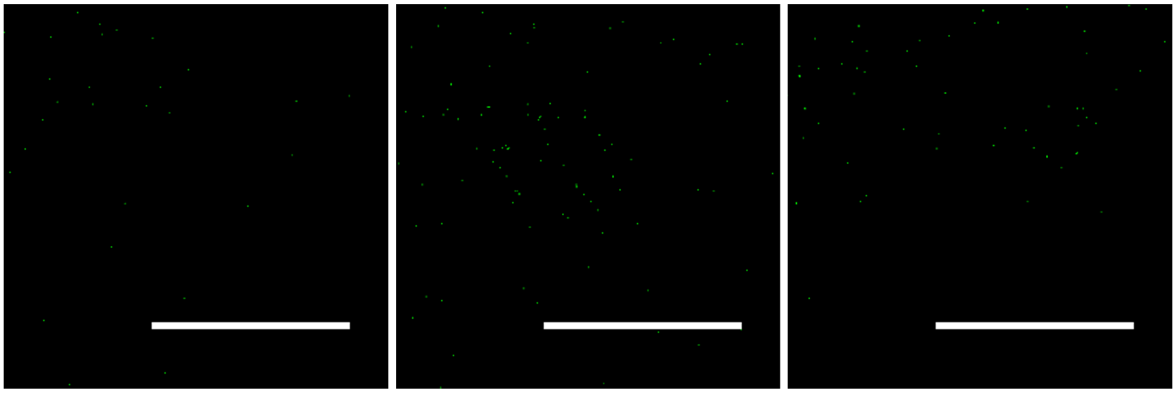

Day 3

33.5 °C – Yeast

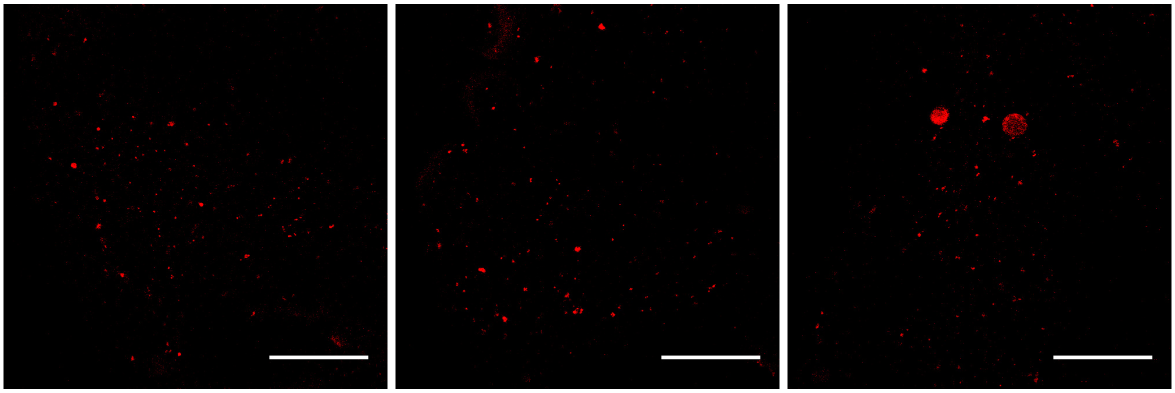

33.5 °C – *E. coli*

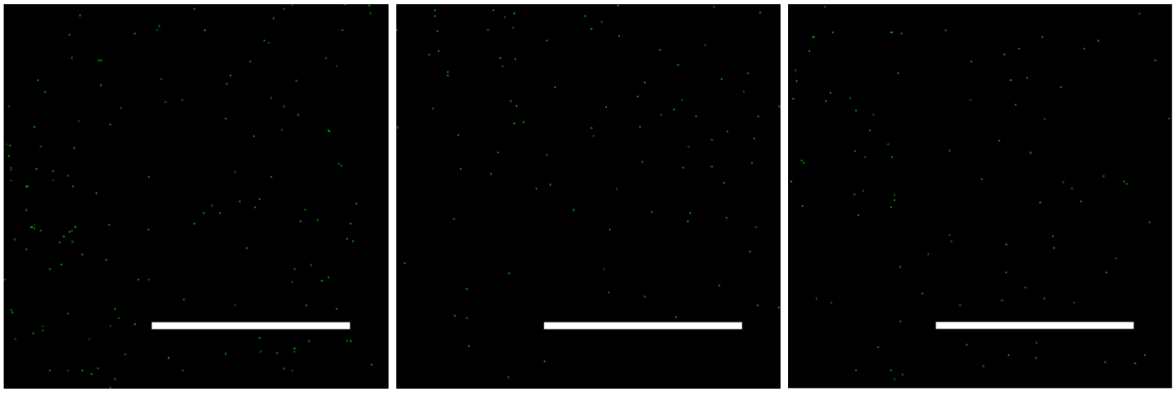

Day 7

33.5 °C – Yeast

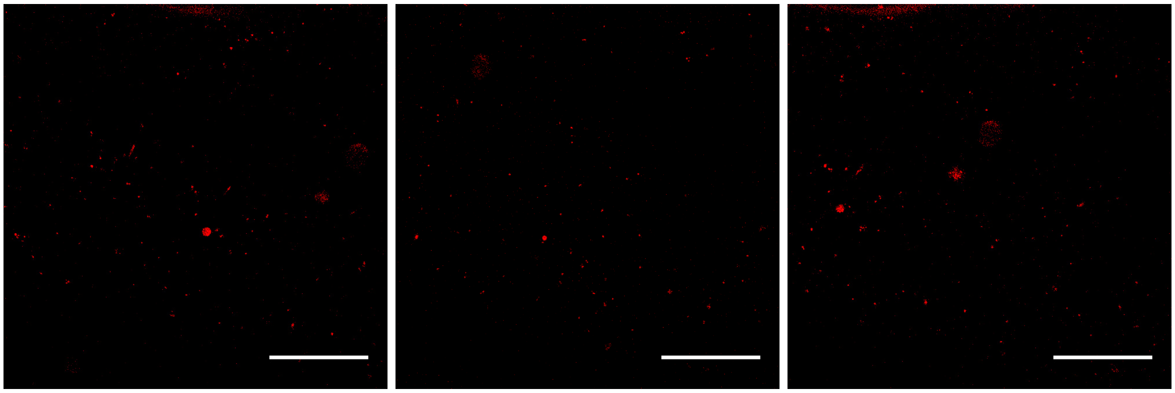

33.5 °C – *E. coli*

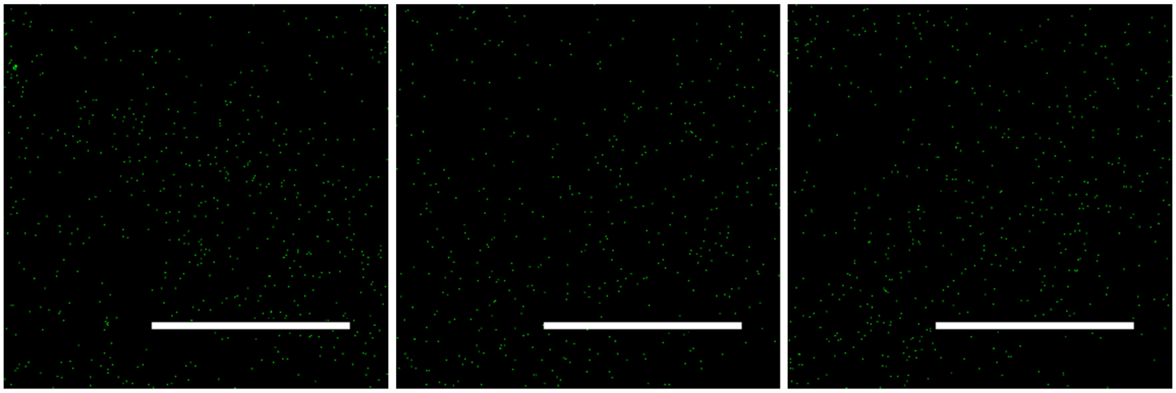

Day 1

37 °C – Yeast

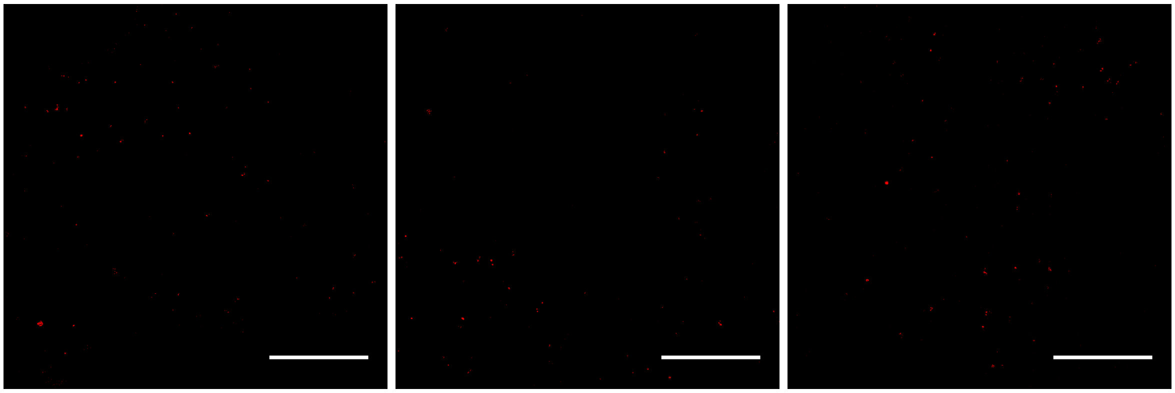

37 °C – *E. coli*

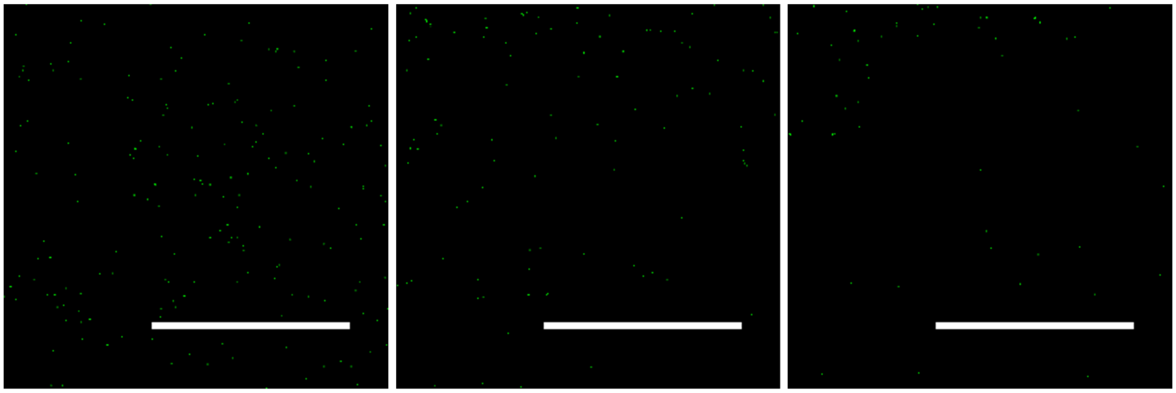

Day 3

37 °C – Yeast

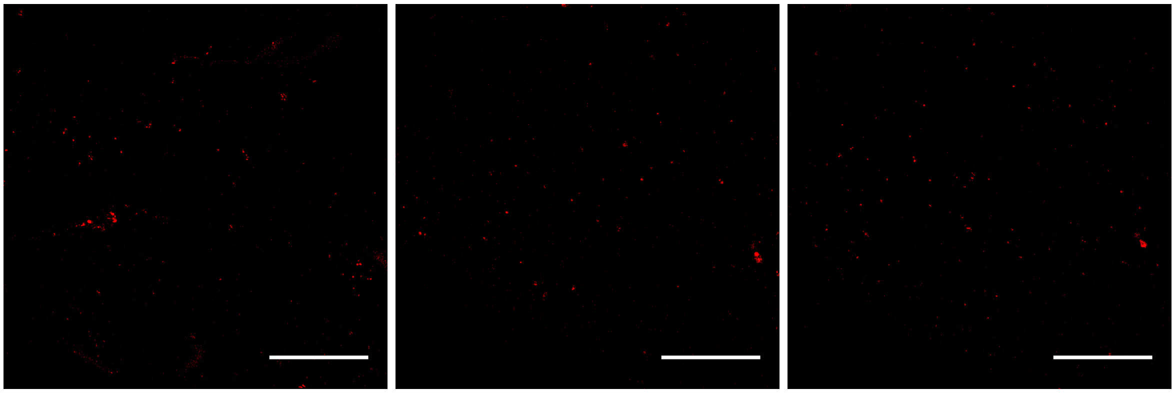

37 °C – *E. coli*

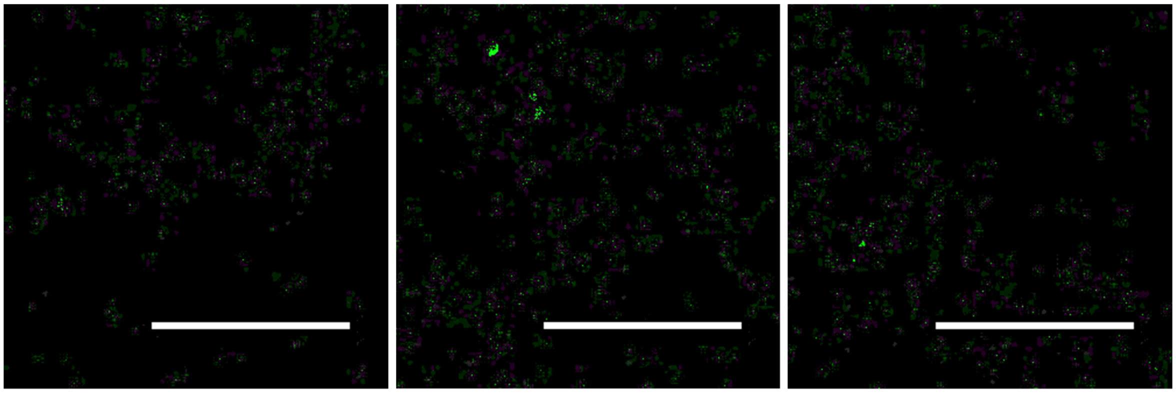

Day 7

37 °C – Yeast

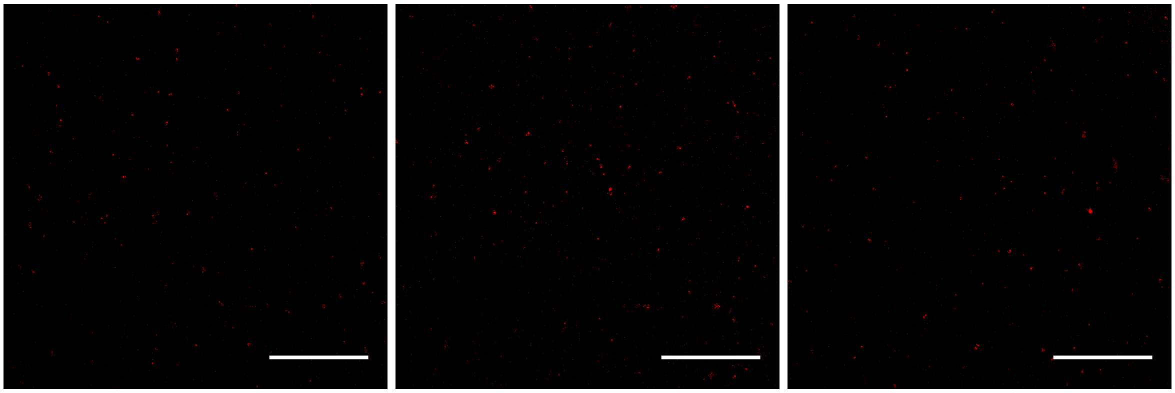

37 °C – *E. coli*

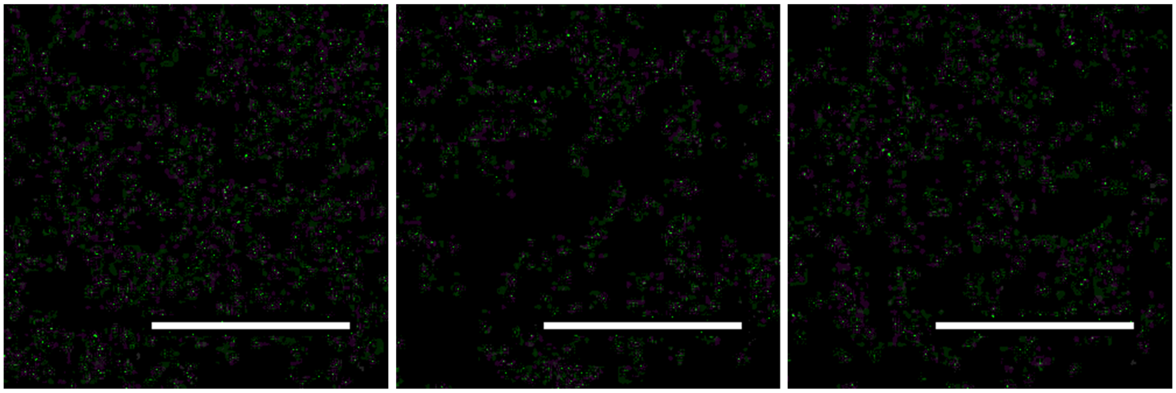

Day 1

40 °C – Yeast

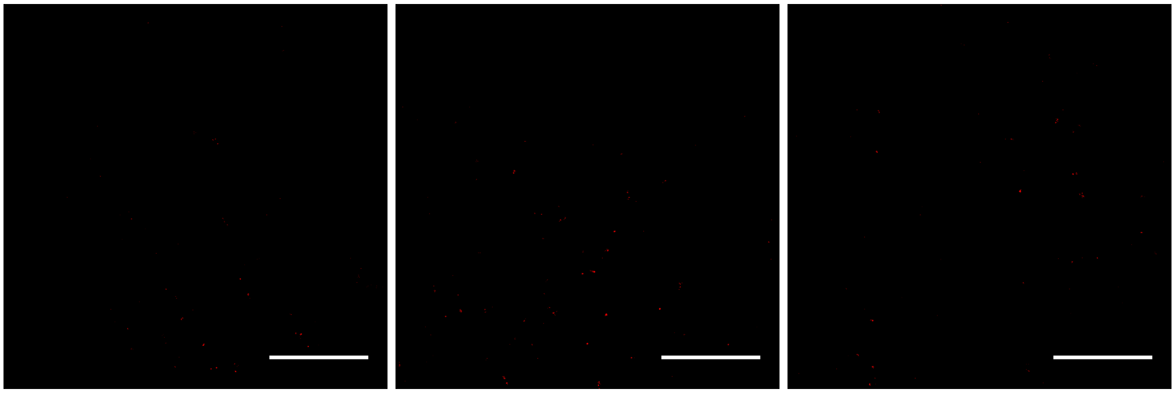

40 °C – *E. coli*

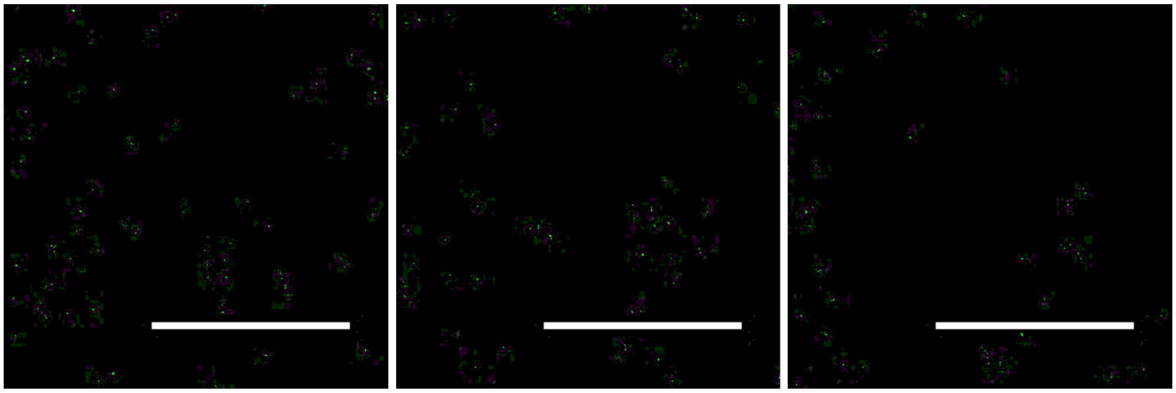

Day 3

40 °C – Yeast

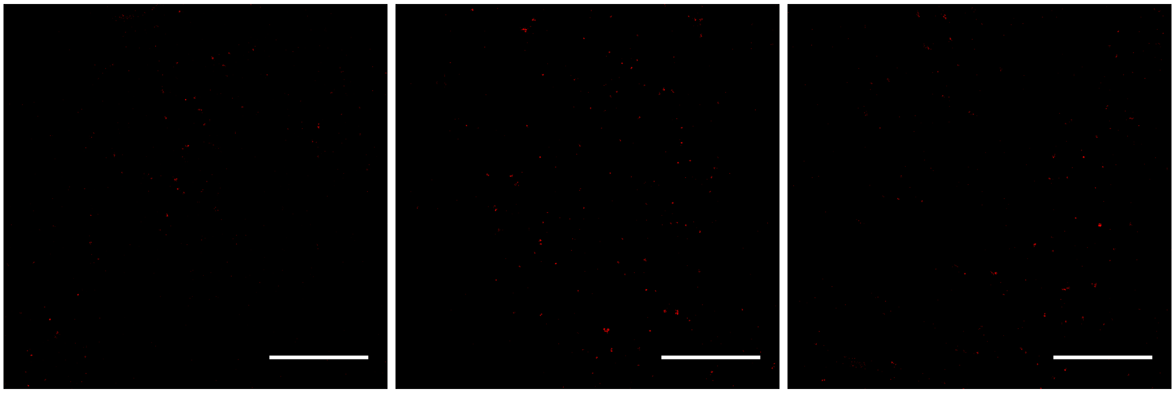

40 °C – *E. coli*

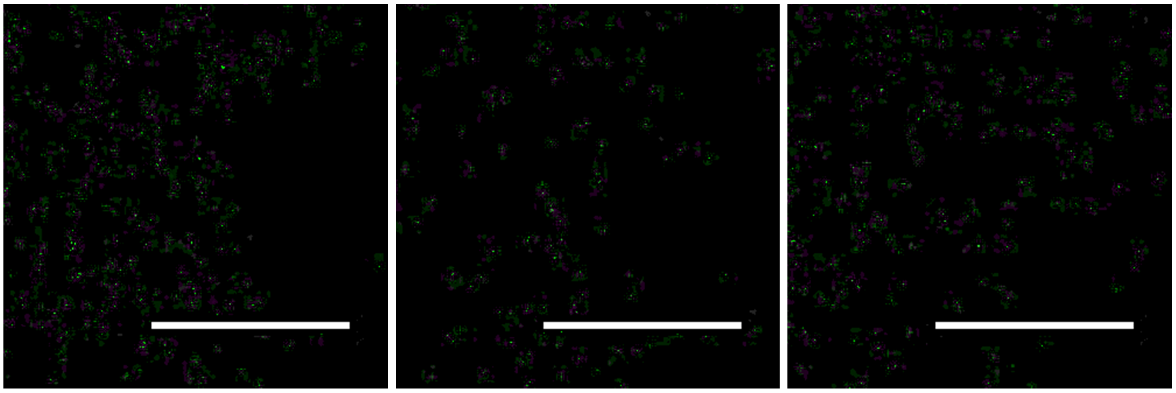

Day 7

40 °C – Yeast

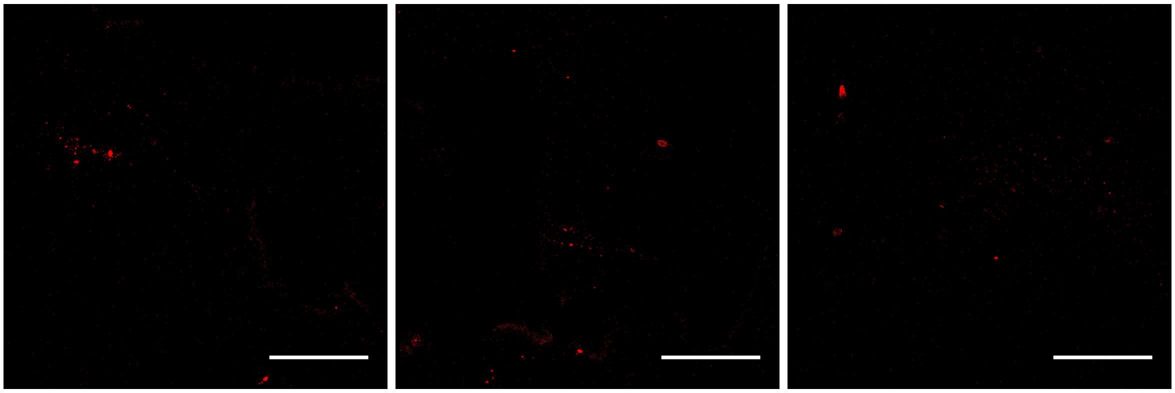

40 °C – *E. coli*

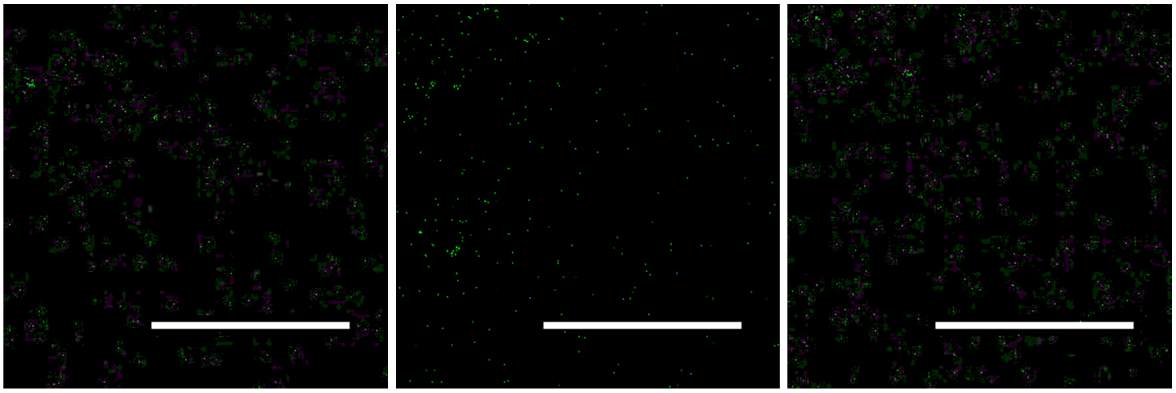

D

Day 1

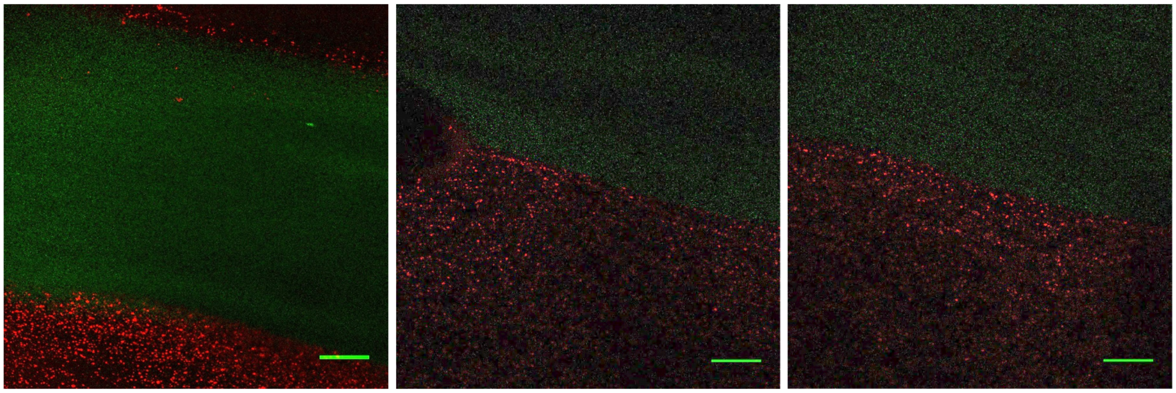

Day 3

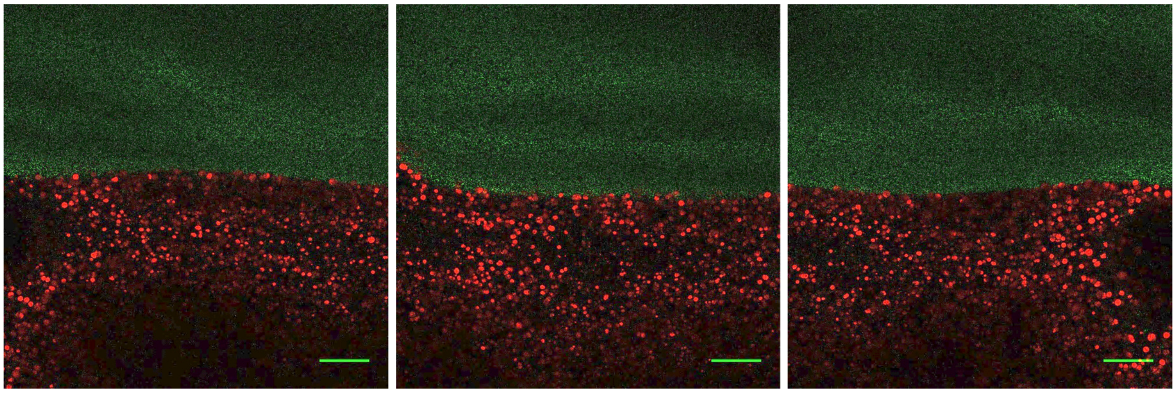

Day 7

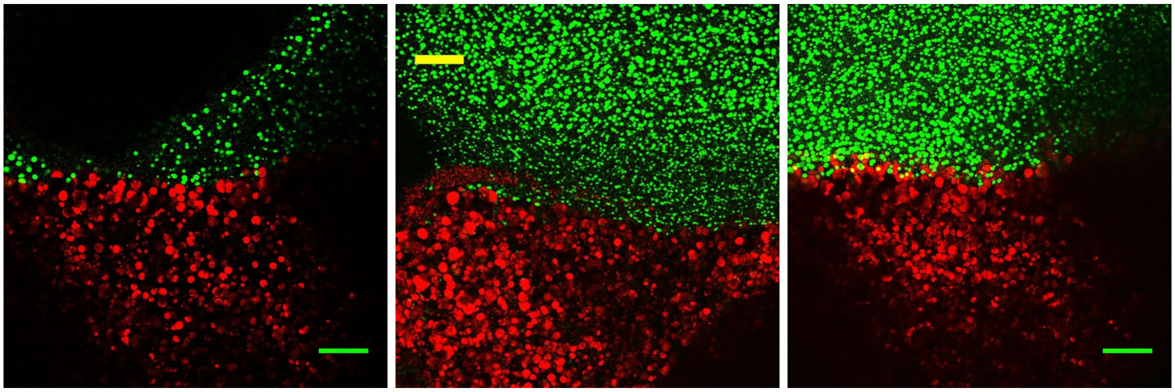

Supplementary Fig. 6. Cell segregation and confluence in extrusion printed co-culture gels. A, Confluence analysis results for the optimal *E. coli* growth condition (37 °C) in consortia hydrogels (left) and optimal yeast growth condition (25 °C) in consortia hydrogels (right). The *E. coli* and yeast samples achieved a confluence of 88.6% and 93.5% on day 7, respectively, in the hydrogel-based consortia relative to the optimal mono-culture gels. **B,** Standard images used in the confluence analysis of cell growth in consortia hydrogels, representing the ideal growth conditions for yeast and *E. coli* monocultures (30 °C in SC media, and 37 °C in LB media, respectively). **C,** Images used for calculation of percent confluence of the microbial consortia hydrogels, at listed temperatures and days of incubation. **D,** Images depicting the cell segregation of bacteria and yeast in their respective hydrogel samples, with little to no movement or mixing of colonies between the hydrogels. The images were captured using confocal microscopy, showing a z-stack of 100 microns of depth at the interface between gel samples printed with RFP yeast and GFP bacteria. All images have 200-micron scale bars. Source data are provided as a Source Data file.

A

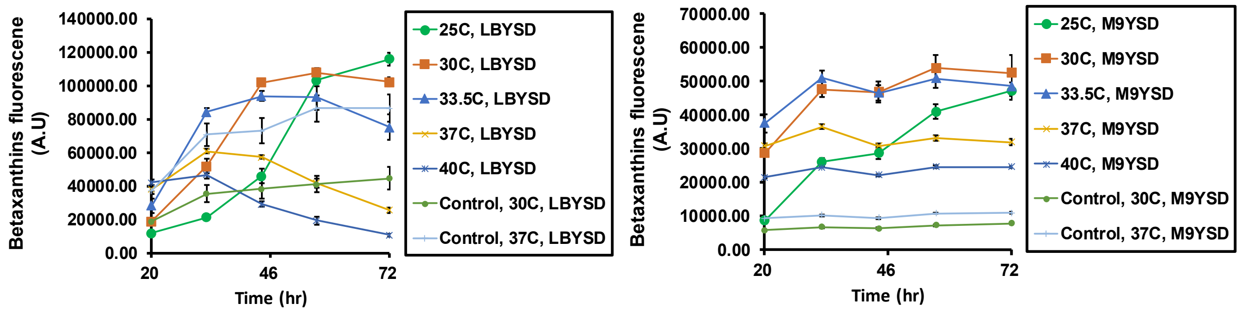
Betaxanthins fluorescene (A.U)
140000.00
120000.00
100000.00
80000.00
60000.00
40000.00
20000.00
0.00
20
46
72
Time (hr)
25C, LBYSD
30C, LBYSD
33.5C, LBYSD
37C, LBYSD
40C, LBYSD
Control, 30C, LBYSD
Control, 37C, LBYSD
Betaxanthins fluorescene (A.U)
70000.00
60000.00
50000.00
40000.00
30000.00
20000.00
10000.00
0.00
20
46
72
Time (hr)
25C, M9YSD
30C, M9YSD
33.5C, M9YSD
37C, M9YSD
40C, M9YSD
Control, 30C, M9YSD
Control, 37C, M9YSD

B

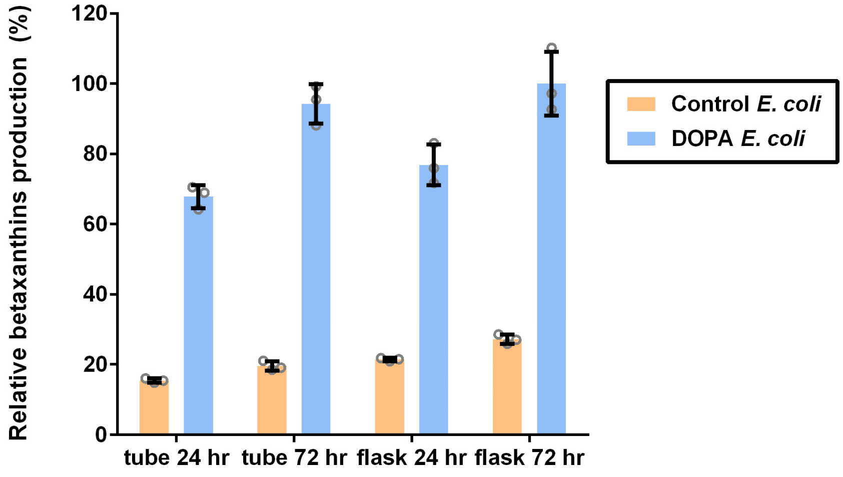
Relative betaxanthins production (%)
120
100
80
60
40
20
0
tube 24 hr
tube 72 hr
flask 24 hr
flask 72 hr
Control E. coli
DOPA E. coli

C

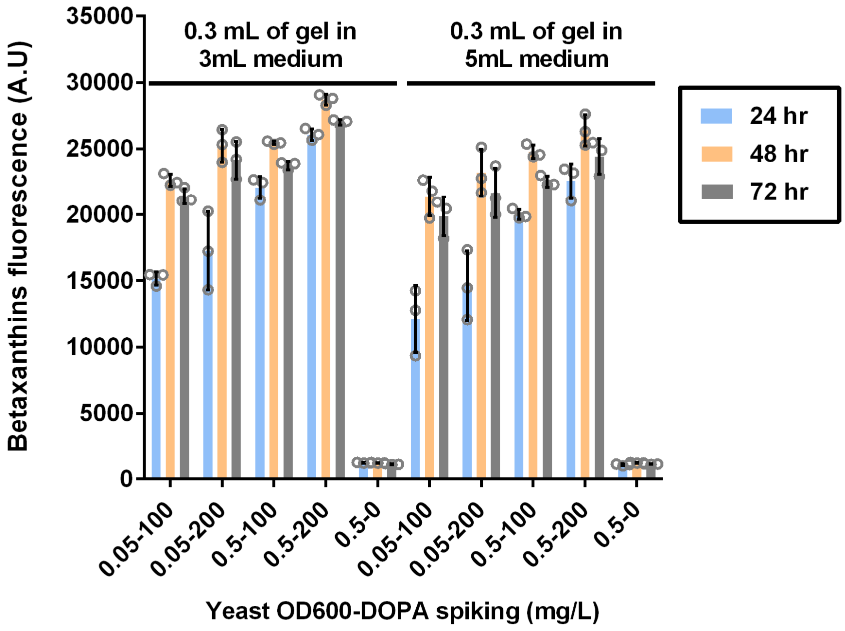
Betaxanthins fluorescence (A.U)
35000
30000
25000
20000
15000
10000
5000
0
0.3 mL of gel in 3mL medium
0.3 mL of gel in 5mL medium
24 hr
48 hr
72 hr
0.05-100
0.05-200
0.5-100
0.5-200
0.5-0
0.05-100
0.05-200
0.5-100
0.5-200
0.5-0
Yeast OD600-DOPA spiking (mg/L)

D

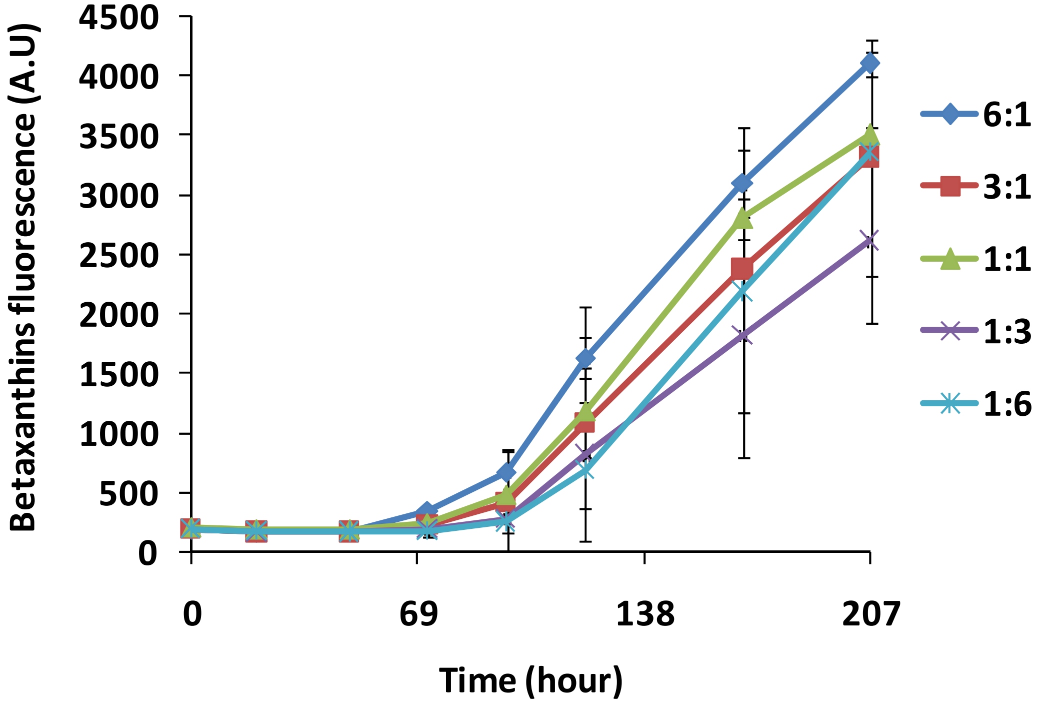
Betaxanthins fluorescence (A.U)
4500
4000
3500
3000
2500
2000
1500
1000
500
0
0
69
138
207
Time (hour)
6:1
3:1
1:1
1:3
1:6

E

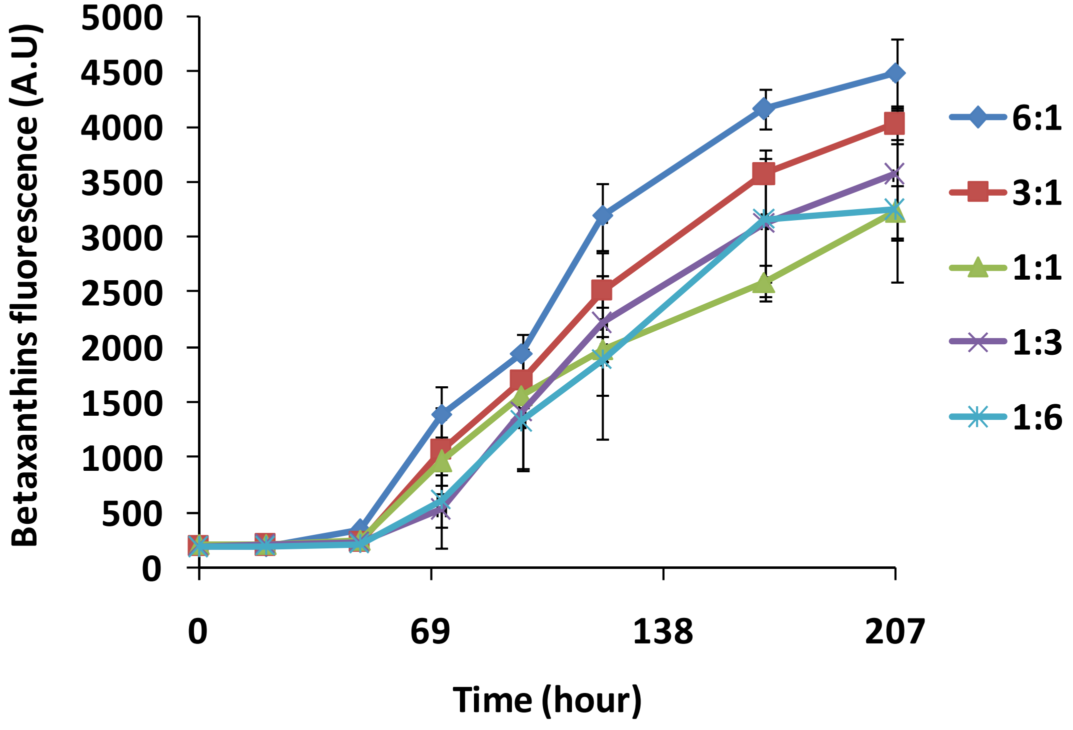
Betaxanthins fluorescence (A.U)
5000
4500
4000
3500
3000
2500
2000
1500
1000
500
0
0
69
138
207
Time (hour)
6:1
3:1
1:1
1:3
1:6

F

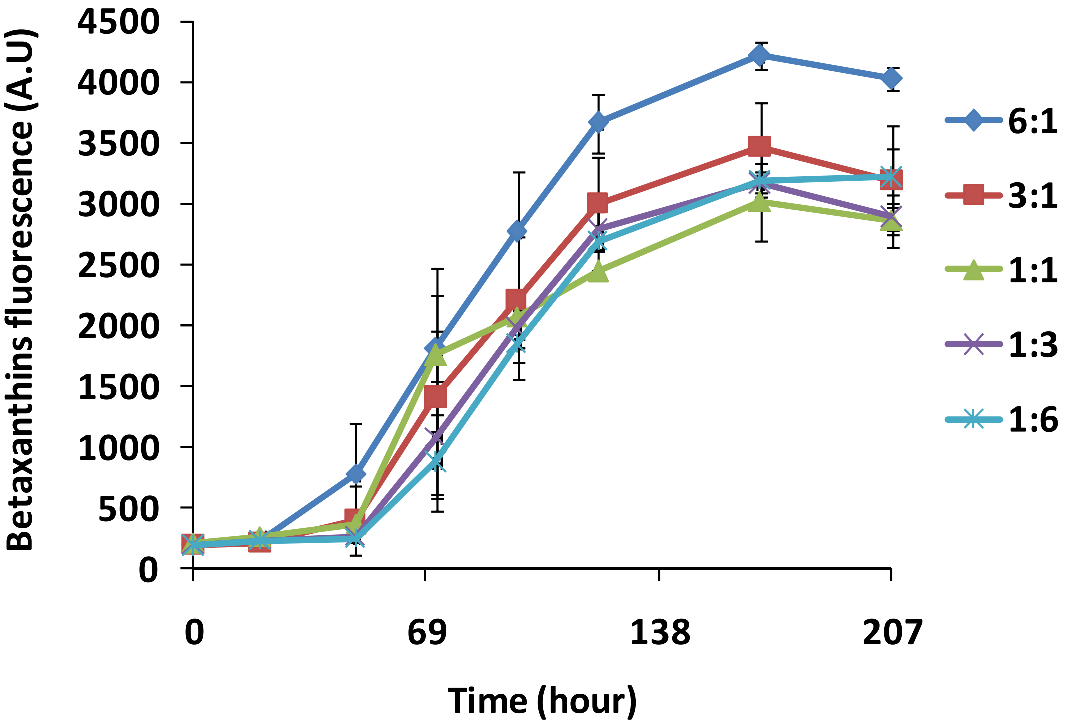
Betaxanthins fluorescence (A.U)
4500
4000
3500
3000
2500
2000
1500
1000
500
0
0
69
138
207
Time (hour)
6:1
3:1
1:1
1:3
1:6

G

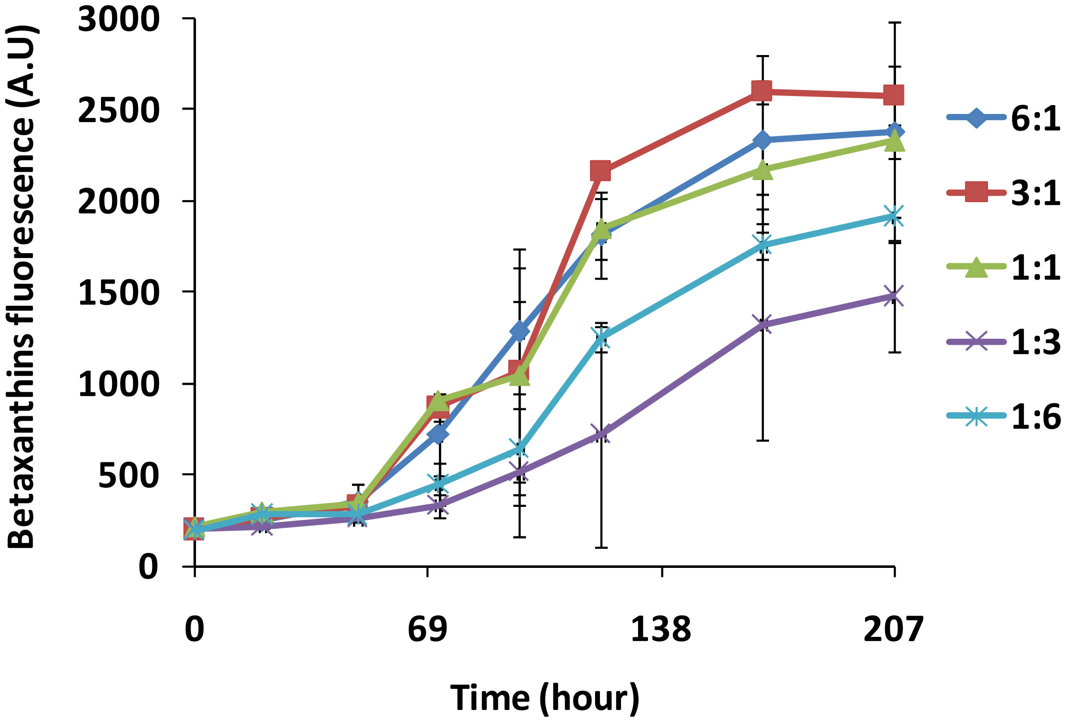
Betaxanthins fluorescence (A.U)
3000
2500
2000
1500
1000
500
0
0
69
138
207
Time (hour)
6:1
3:1
1:1
1:3
1:6

H

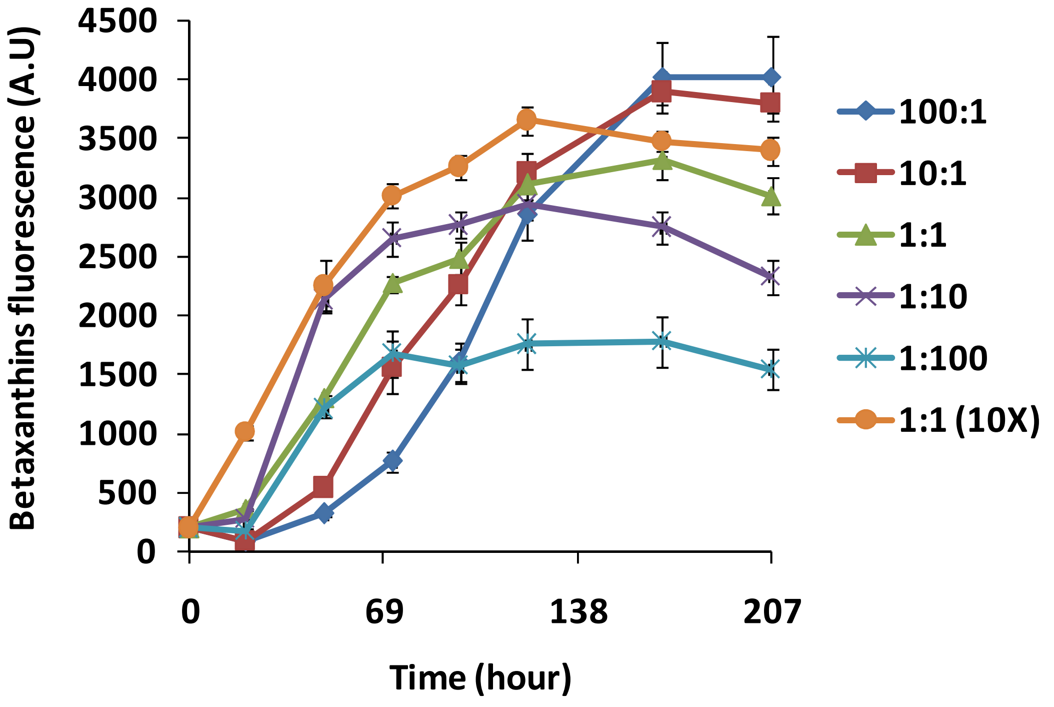

4500
4000
3500
3000
2500
2000
1500
1000
500
0
Betaxanthins fluorescence (A.U)
0
69
138
207
Time (hour)
100:1
10:1
1:1
1:10
1:100
1:1 (10X)

I

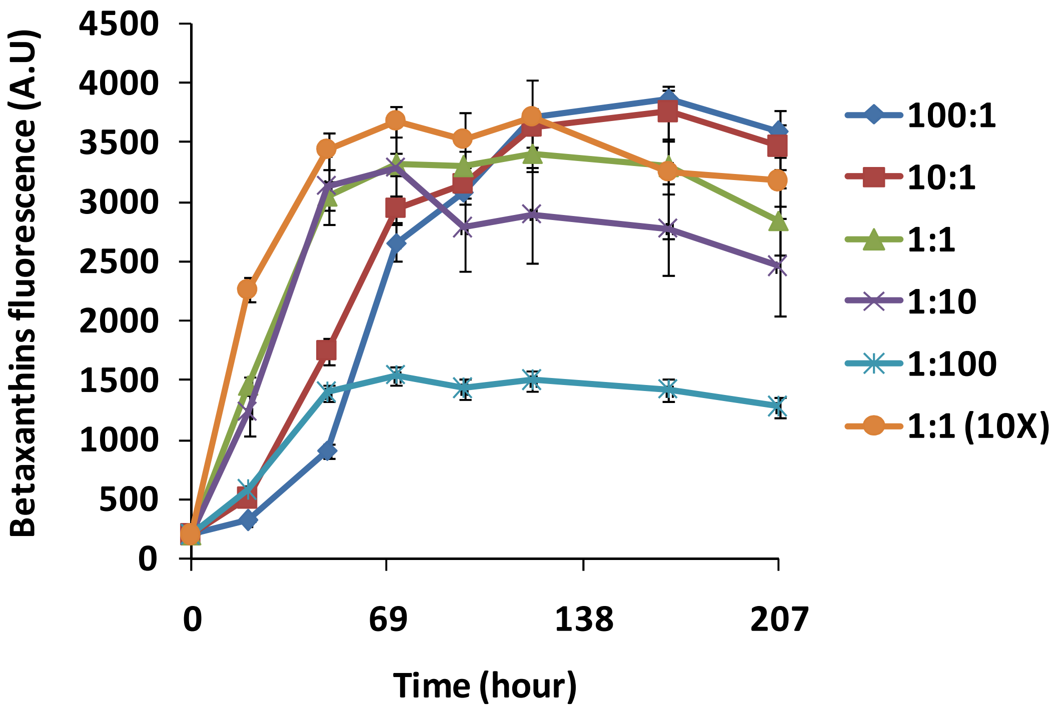

4500
4000
3500
3000
2500
2000
1500
1000
500
0
Betaxanthins fluorescence (A.U)
0
69
138
207
Time (hour)
100:1
10:1
1:1
1:10
1:100
1:1 (10X)

J

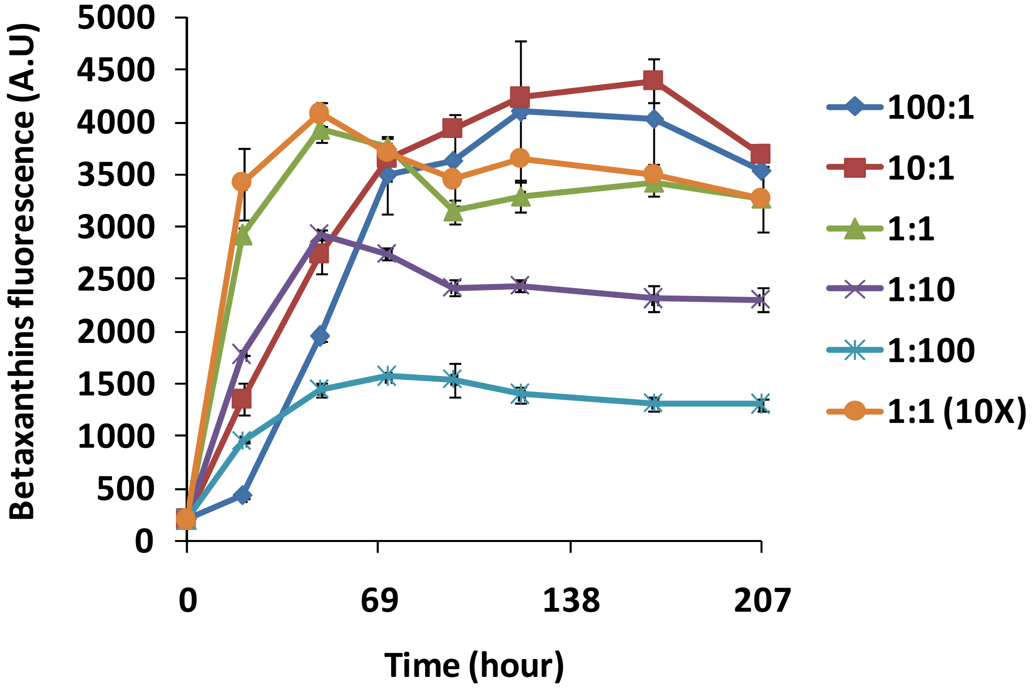

Betaxanthins fluorescence (A.U)
5000
4500
4000
3500
3000
2500
2000
1500
1000
500
0
0
69
138
207
Time (hour)
100:1
10:1
1:1
1:10
1:100
1:1 (10X)

K

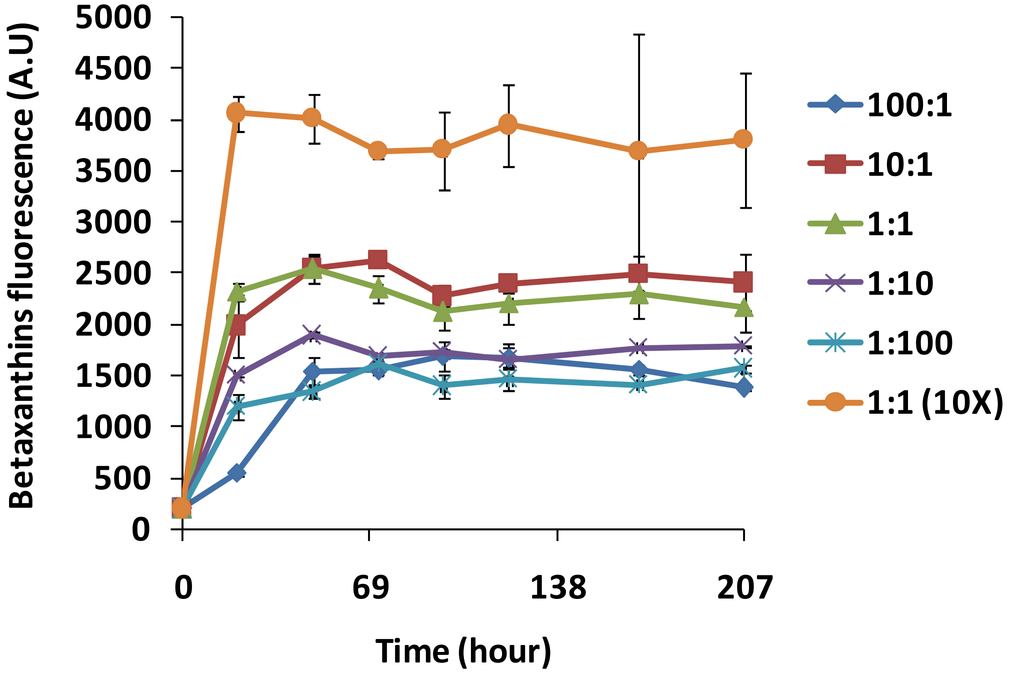

Betaxanthins fluorescence (A.U)
5000
4500
4000
3500
3000
2500
2000
1500
1000
500
0
0
69
138
207
Time (hour)
100:1
10:1
1:1
1:10
1:100
1:1 (10X)

L

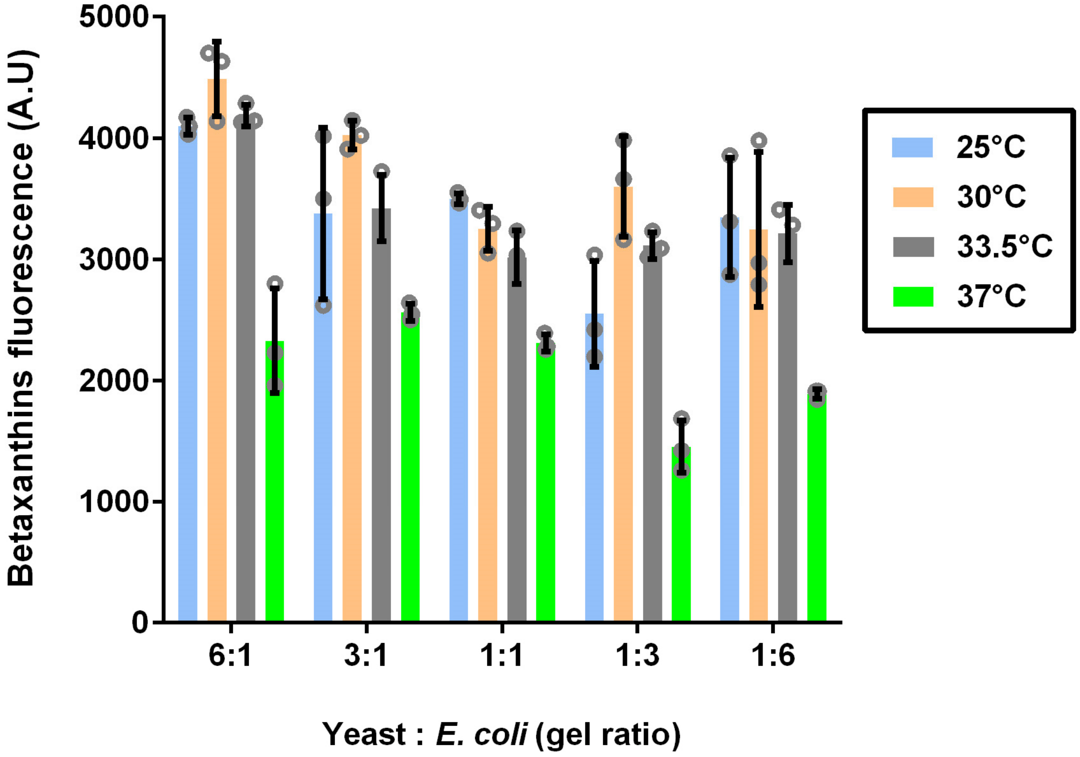

Betaxanthins fluorescence (A.U)
5000
4000
3000
2000
1000
0
6:1
3:1
1:1
1:3
1:6
Yeast : E. coli (gel ratio)
25°C
30°C
33.5°C
37°C

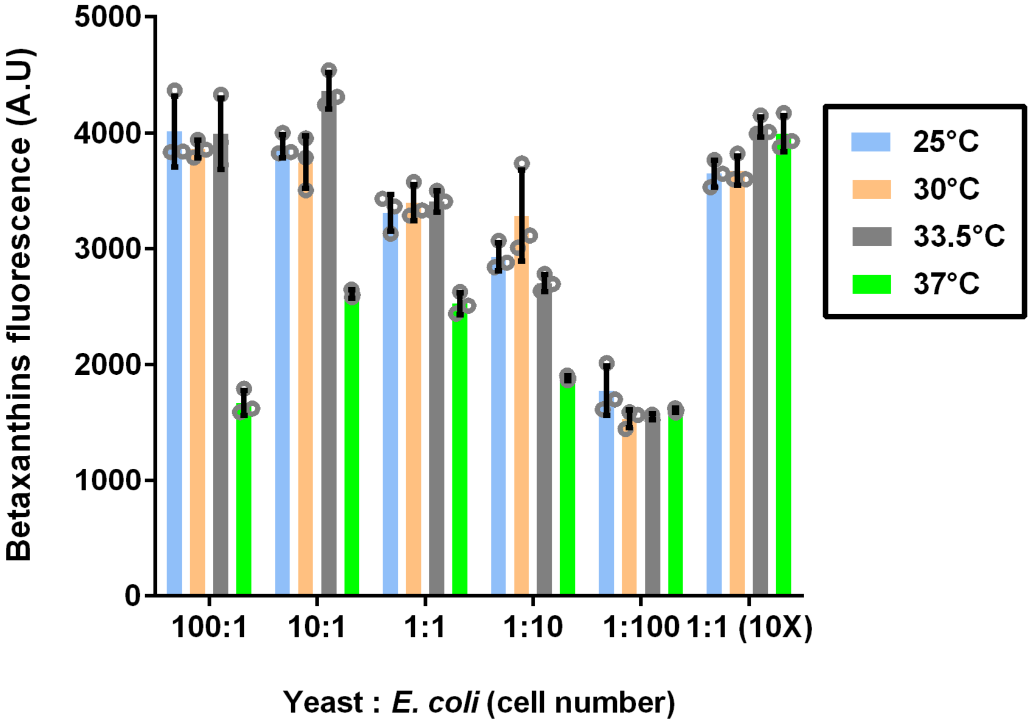

Betaxanthins fluorescence (A.U)
5000
4000
3000
2000
1000
0
100:1
10:1
1:1
1:10
1:100
1:1 (10X)
Yeast : E. coli (cell number)
25°C
30°C
33.5°C
37°C

Supplementary Fig. 7. Betaxanthins production. A, Medium optimization for betaxanthins production with different temperatures. Yeast sBY08 and *E. coli* eBL0430D strains were used for this study. Production was evaluated using LBYSD (left) or M9YSD medium (right) supplemented with 20 g/L glucose and appropriate amount of antibiotics. **B,** The comparison of betaxanthins production for bulk culture between tube and flask scales. Betaxanthins fluorescence was measured at 24 and 72-hour time points. While production at flask scale works better than that at tube scale, probably because flask provides more oxygen transfer (DOD reaction requires oxygen), tube scale was chosen for the following experiments for operational convenience. **C,** Betaxanthins production via DOD yeast-laden hydrogel. The comparison of betaxanthins production for yeast-laden gel between 3 mL and 5mL gel culture volume. 3 mL culture volume was selected for the following experiments due to higher betaxanthins production possibly resulted from better oxygenation compared to that using 5 mL culture condition. **D-G,** Betaxanthins production via *E. coli*-yeast consortia gels in round 0. The productions are evaluated through altering gel ratios and fermentation temperatures. Time course of betaxanthins production for hydrogel system at 25 (**D**), 30 (**E**), 33.5 (**F**) and 37 (**G**) °C. **H-K,** Betaxanthins production via *E. coli*-yeast bulk culture in round 0. The productions are evaluated through altering cell number and fermentation temperatures. Time course of betaxanthins production for bulk culture at 25 (**H**), 30 (**I**), 33.5 (**J**) and 37 (**K**) °C. **L,** The comparison of maximum production of betaxanthins in the round 0 between hydrogel system (top) and liquid culture (bottom). Source data are provided as a Source Data file.

A

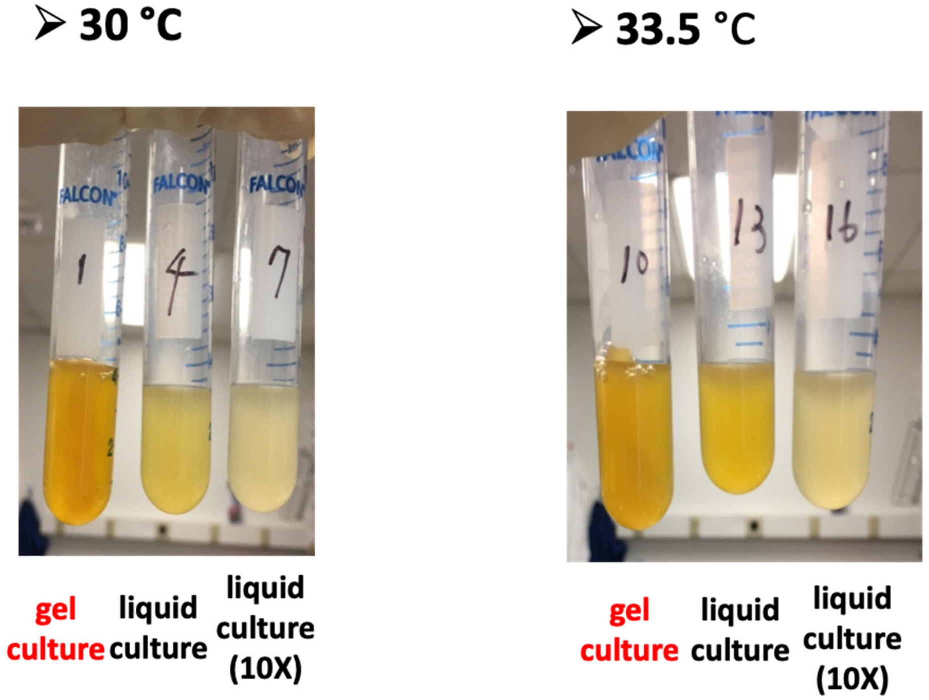

➢ 30 °C
➢ 33.5 °C
1
4
7
10
13
16
gel culture
liquid culture
liquid culture (10X)
gel culture
liquid culture
liquid culture (10X)

B

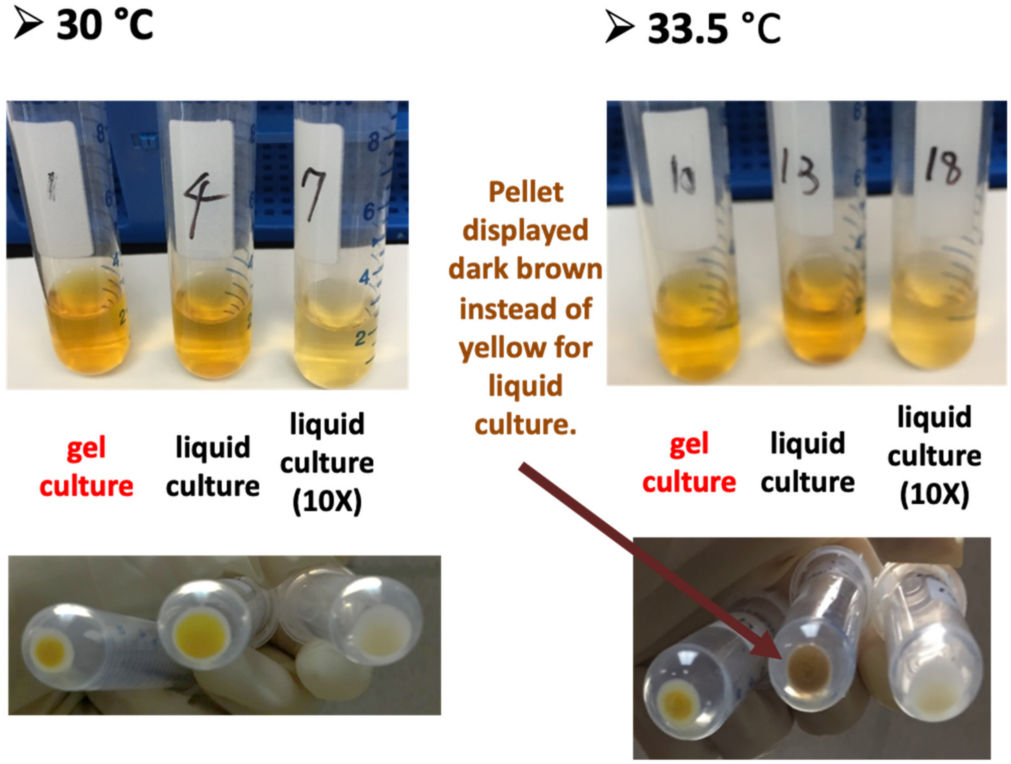

➢ 30 °C
➢ 33.5 °C
4
7
10
13
18
gel culture
liquid culture
liquid culture (10X)
Pellet displayed dark brown instead of yellow for liquid culture.
gel culture
liquid culture
liquid culture (10X)

C

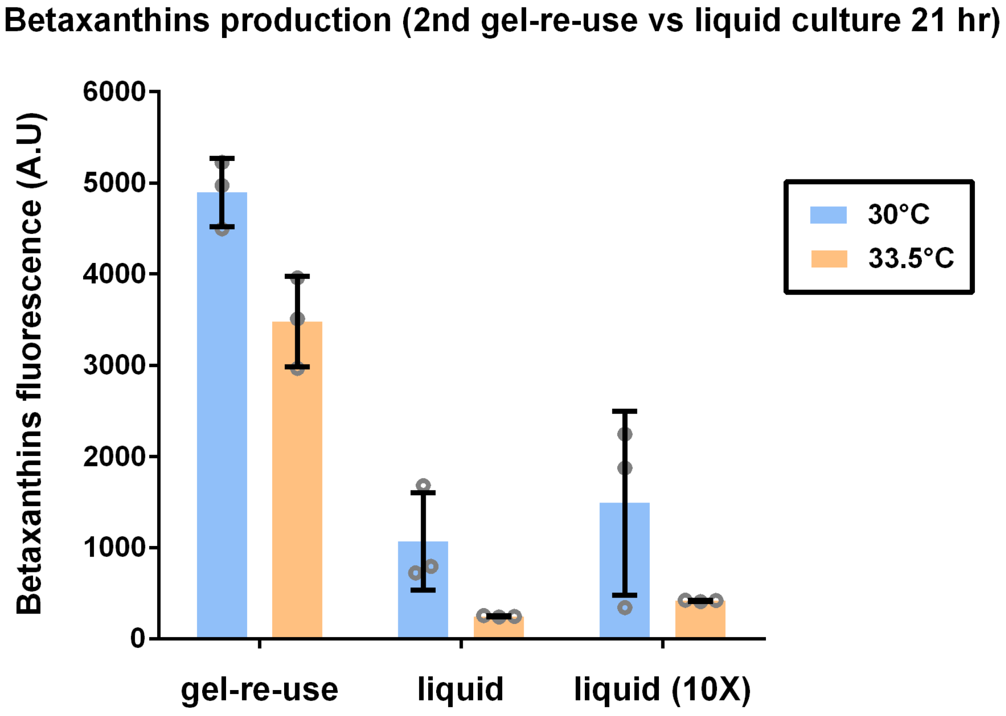
Betaxanthins production (2nd gel-re-use vs liquid culture 21 hr)
Betaxanthins fluorescence (A.U)
6000
5000
4000
3000
2000
1000
0
gel-re-use
liquid
liquid (10X)
30°C
33.5°C

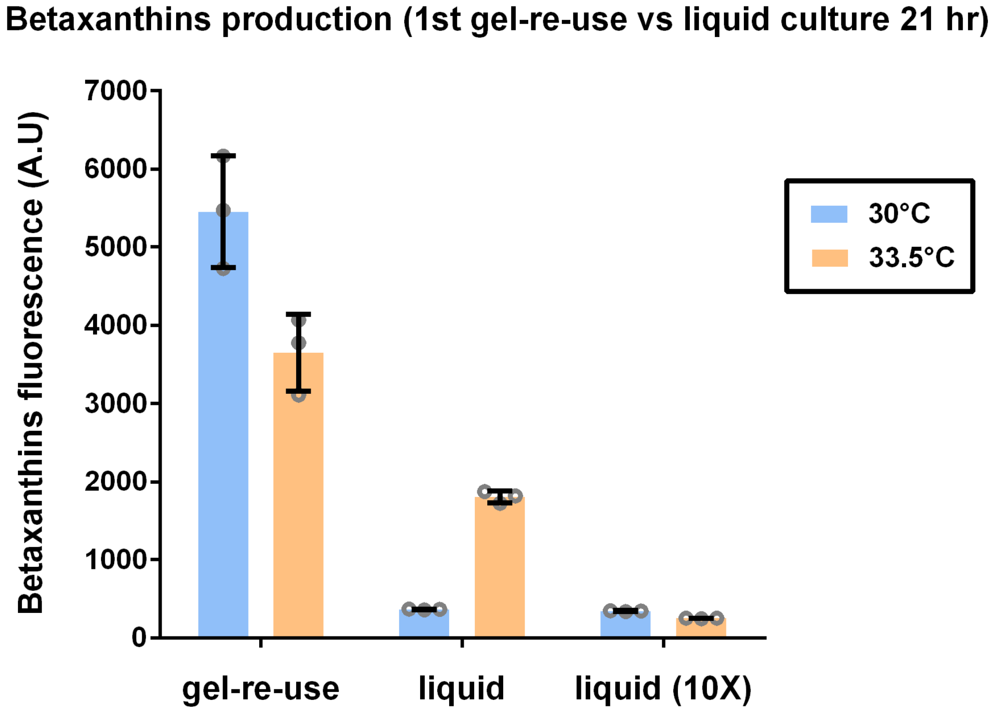
Betaxanthins production (1st gel-re-use vs liquid culture 21 hr)
Betaxanthins fluorescence (A.U)
7000
6000
5000
4000
3000
2000
1000
0
gel-re-use
liquid
liquid (10X)
30°C
33.5°C

D

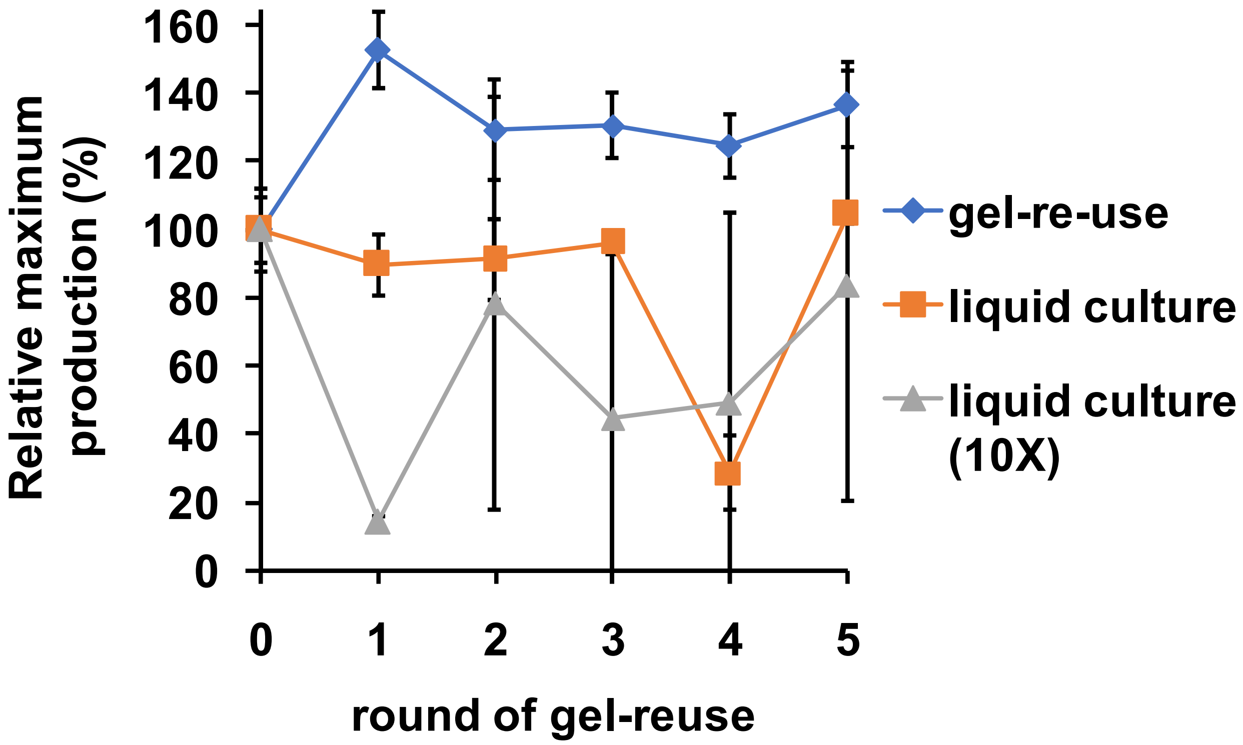

160
140
120
100
80
60
40
20
0
Relative maximum production (%)
0
1
2
3
4
5
round of gel-reuse
gel-re-use
liquid culture
liquid culture (10X)

E

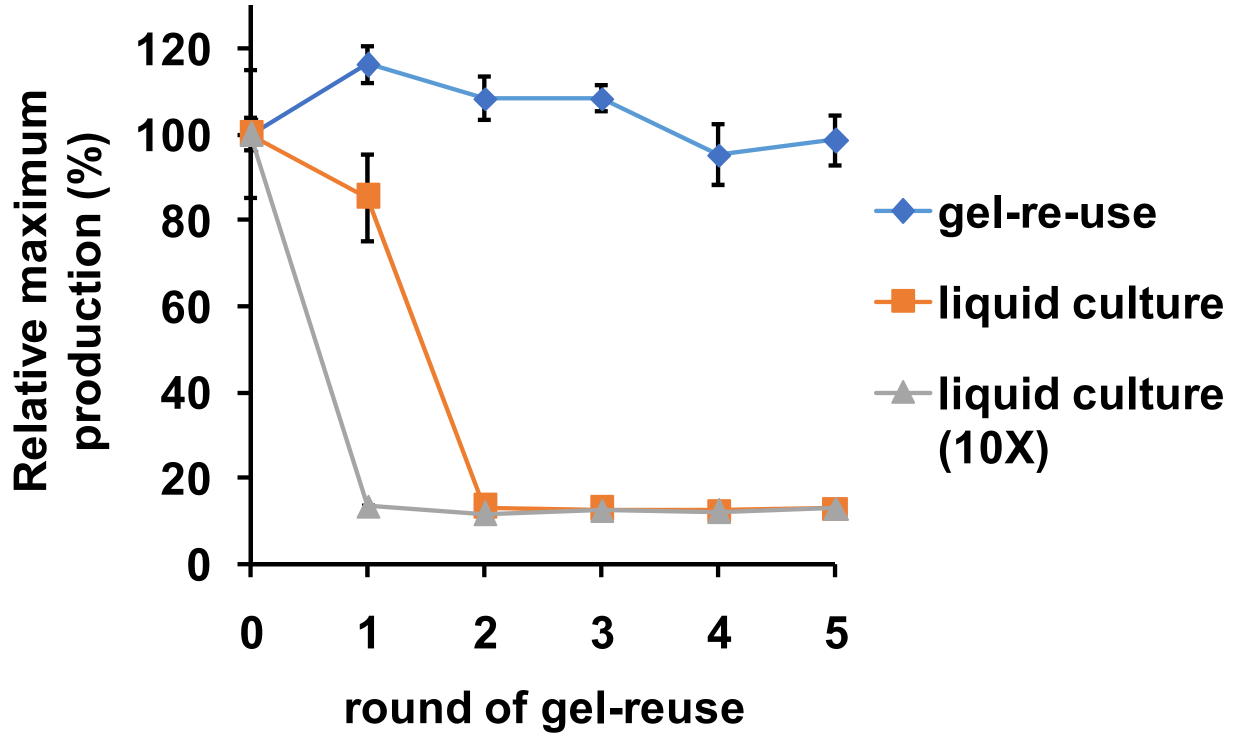

120
100
80
60
40
20
0
Relative maximum production (%)
0
1
2
3
4
5
round of gel-reuse
gel-re-use
liquid culture
liquid culture (10X)

Supplementary Fig. 8. Gel-re-run for betaxanthins production via *E. coli*-yeast consortia. Cell-laden gels were removed from round 0. The performances between each sample were compared at 21 (**A**) and 96 hr (**B**) fermentation for the 1st round of gel-re-use. **C,** The consortia activity between the 1st and 2nd round of gel-re-use were compared. Liquid culture in the 2nd round incubated at 30 °C for 21 hr (bottom) produced more betaxanthins than that in the 1st round (top). These results show that liquid culture can't easily control the consortia dynamics for the cell recycle batch fermentation purpose. The comparison of maximum betaxanthins production at 30 (**D**) and 33.5 °C (**E**) between gel and liquid culture for 5 consecutive cell-reuse cycles. Source data are provided as a Source Data file.

A

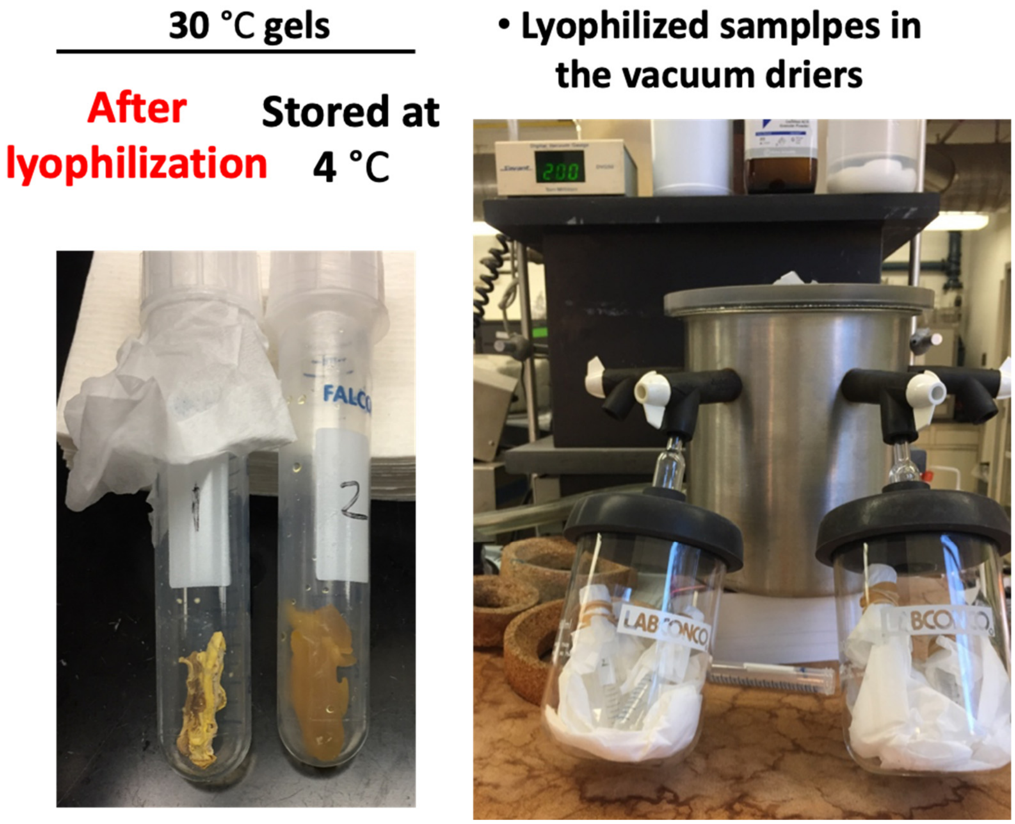
30 °C gels
After lyophilization
Stored at 4 °C
• Lyophilized samplpes in the vacuum driers

B

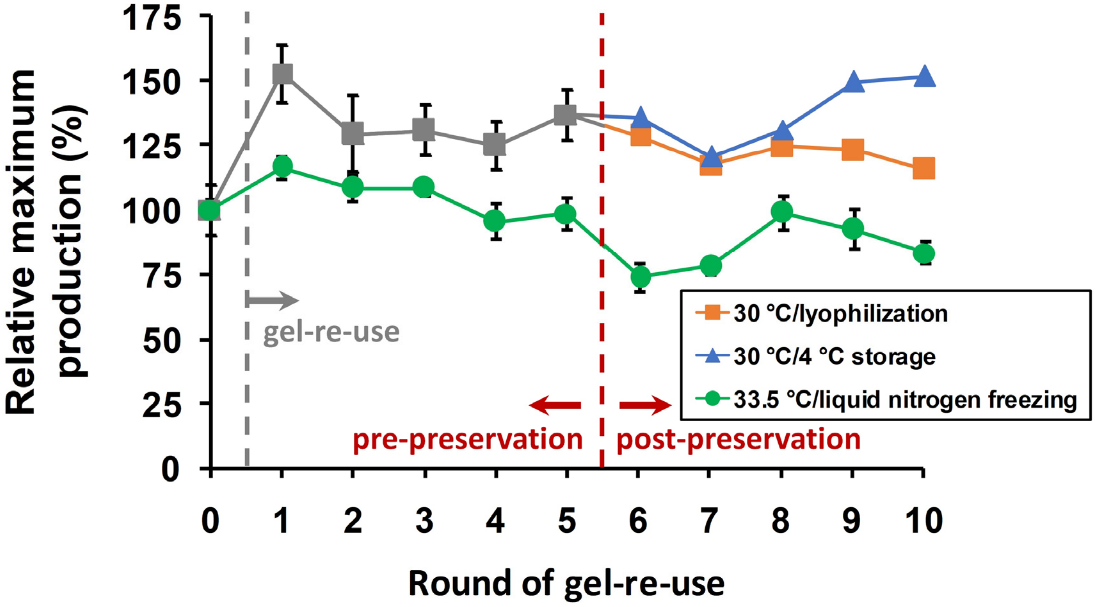
Relative maximum production (%)
175
150
125
100
75
50
25
0
0 1 2 3 4 5 6 7 8 9 10
Round of gel-re-use
gel-re-use
pre-preservation
post-preservation
30 °C/lyophilization
30 °C/4 °C storage
33.5 °C/liquid nitrogen freezing

C

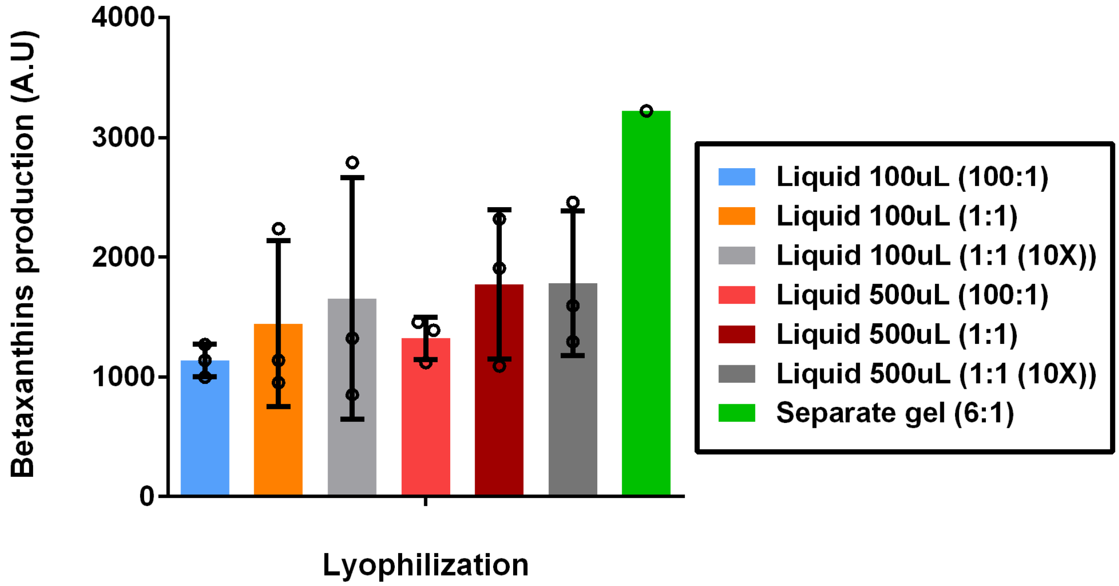

Betaxanthins production (A.U)
4000
3000
2000
1000
0
Lyophilization
Liquid 100uL (100:1)
Liquid 100uL (1:1)
Liquid 100uL (1:1 (10X))
Liquid 500uL (100:1)
Liquid 500uL (1:1)
Liquid 500uL (1:1 (10X))
Separate gel (6:1)

D

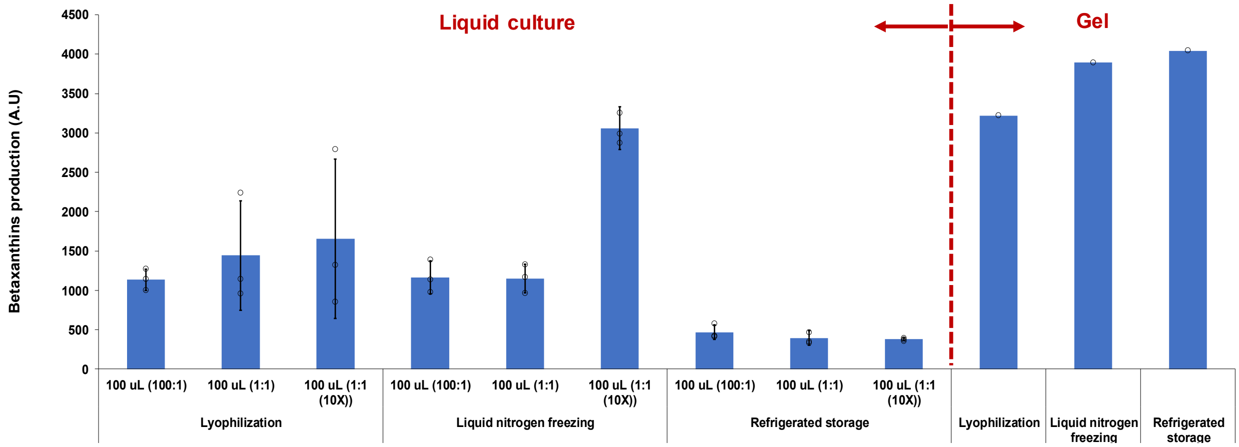

Betaxanthins production (A.U)
Liquid culture
Gel
4500
4000
3500
3000
2500
2000
1500
1000
500
0
100 uL (100:1)
100 uL (1:1)
100 uL (1:1 (10X))
Lyophilization
Liquid nitrogen freezing
Refrigerated storage
Lyophilization
Liquid nitrogen freezing
Refrigerated storage

E

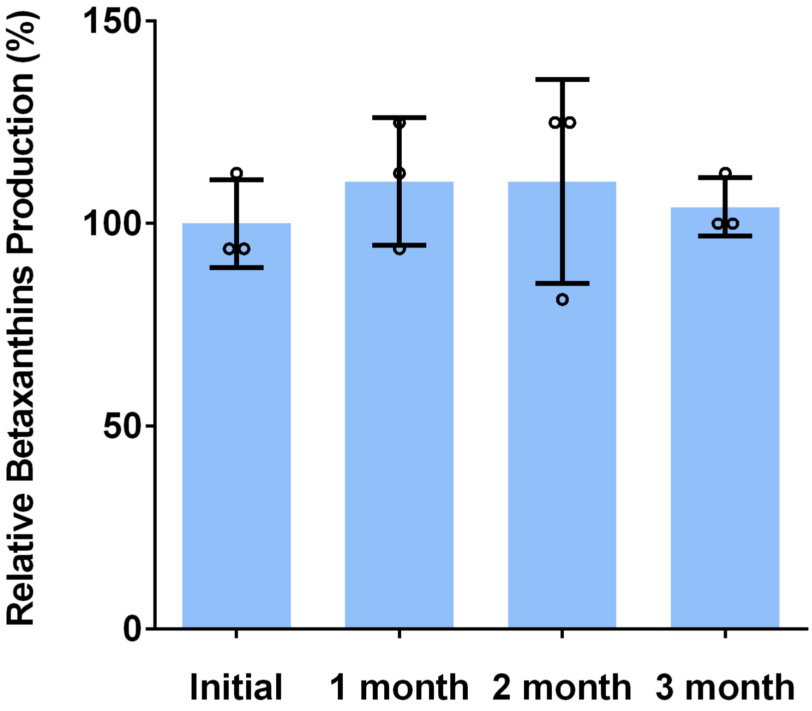

Relative Betaxanthins Production (%)
150
100
50
0
Initial
1 month
2 month
3 month

F

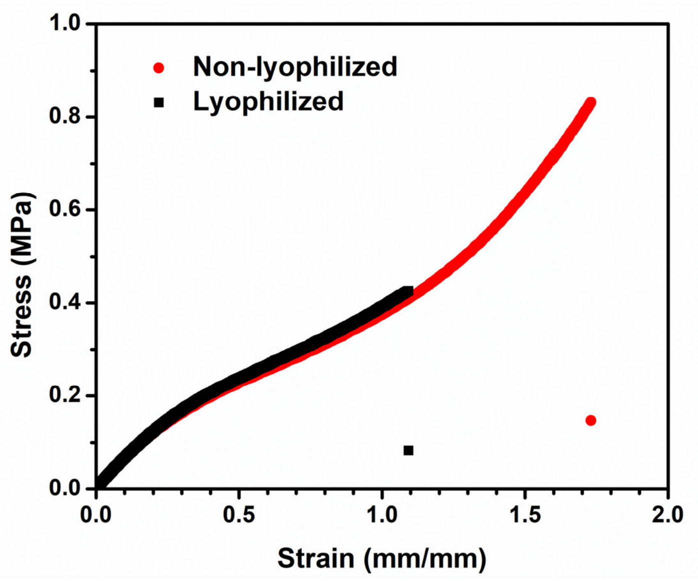

	E (MPa)	σ (MPa)	ε
Non-lyophilized	0.67 ± 0.03	0.83 ± 0.11	1.73 ± 0.21
Lyophilized	0.67 ± 0.02	0.43 ± 0.07	1.09 ± 0.17

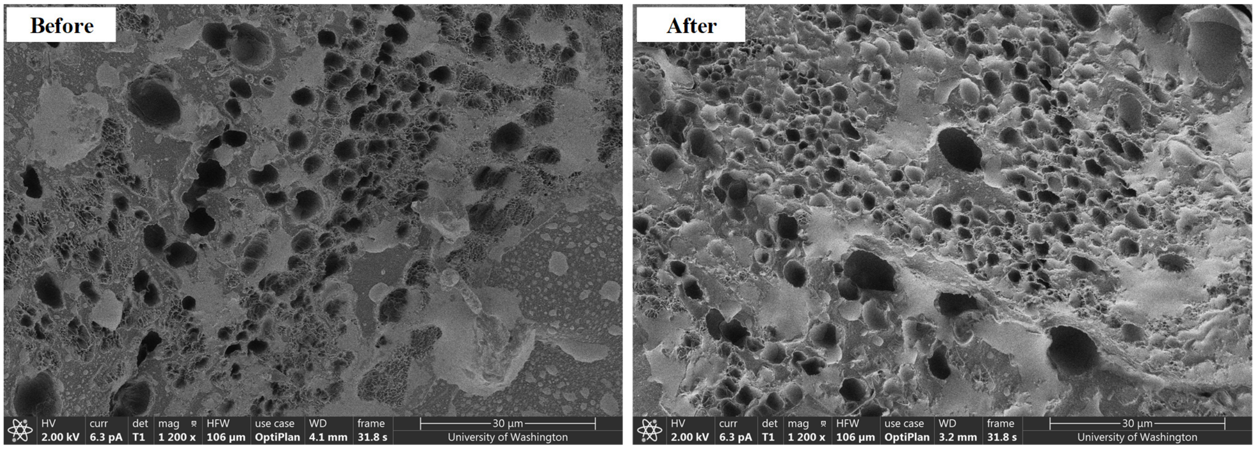

G

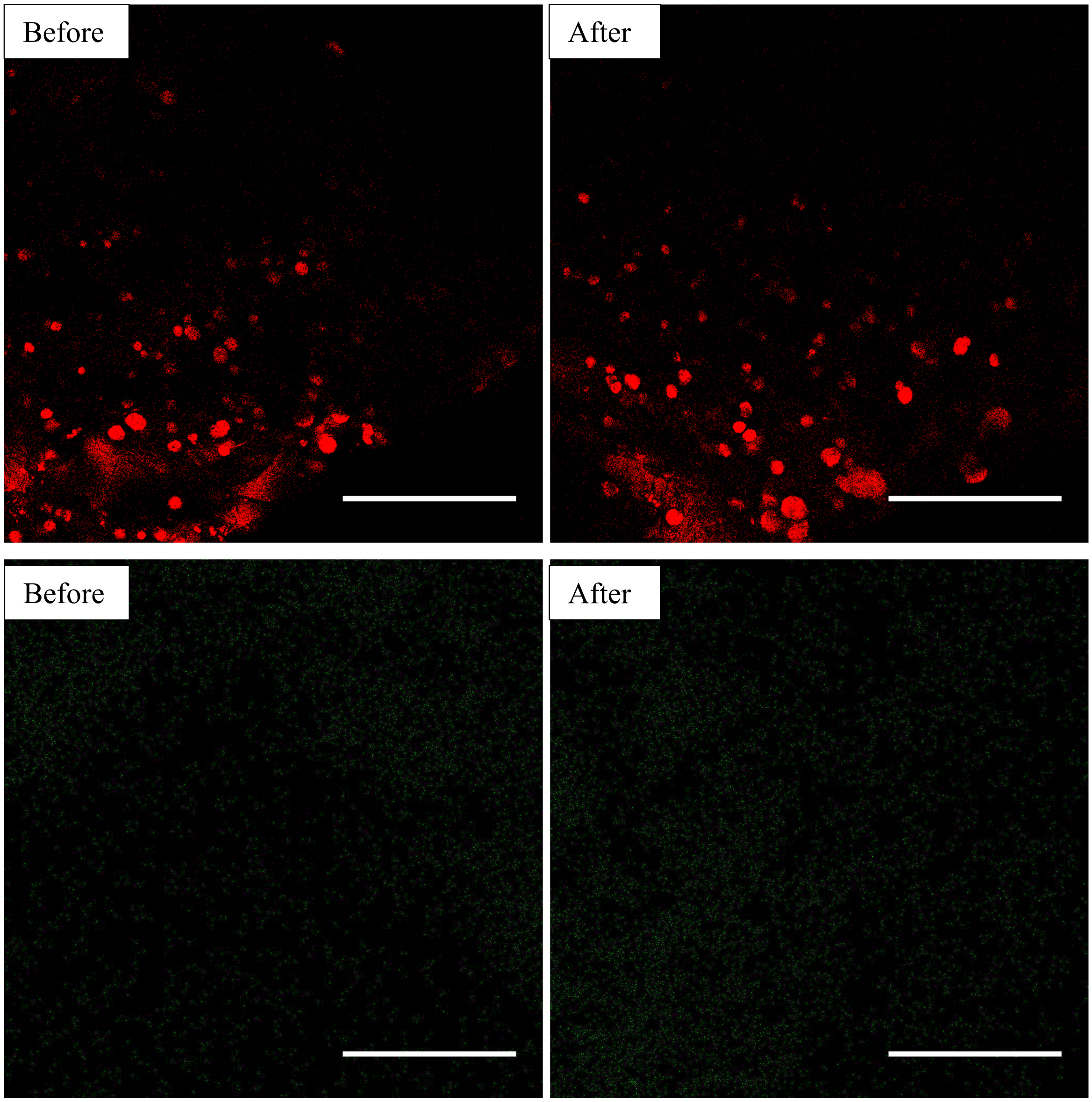

H

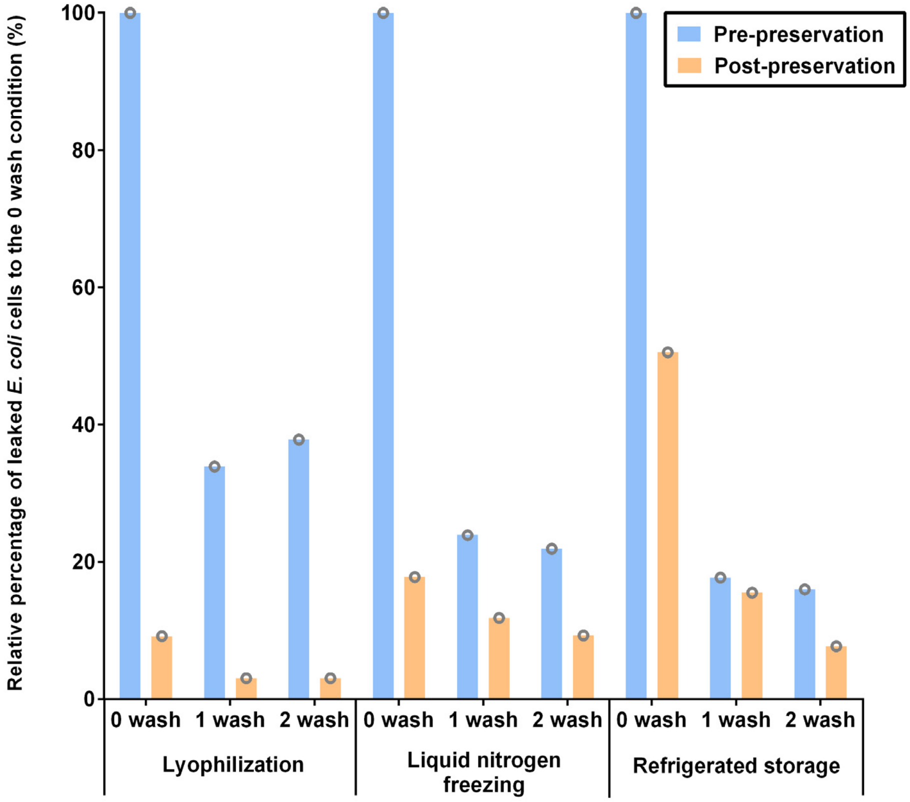
Relative percentage of leaked E. coli cells to the 0 wash condition (%)
100
80
60
40
20
0
Pre-preservation
Post-preservation
0 wash
1 wash
2 wash
Lyophilization
Liquid nitrogen freezing
Refrigerated storage

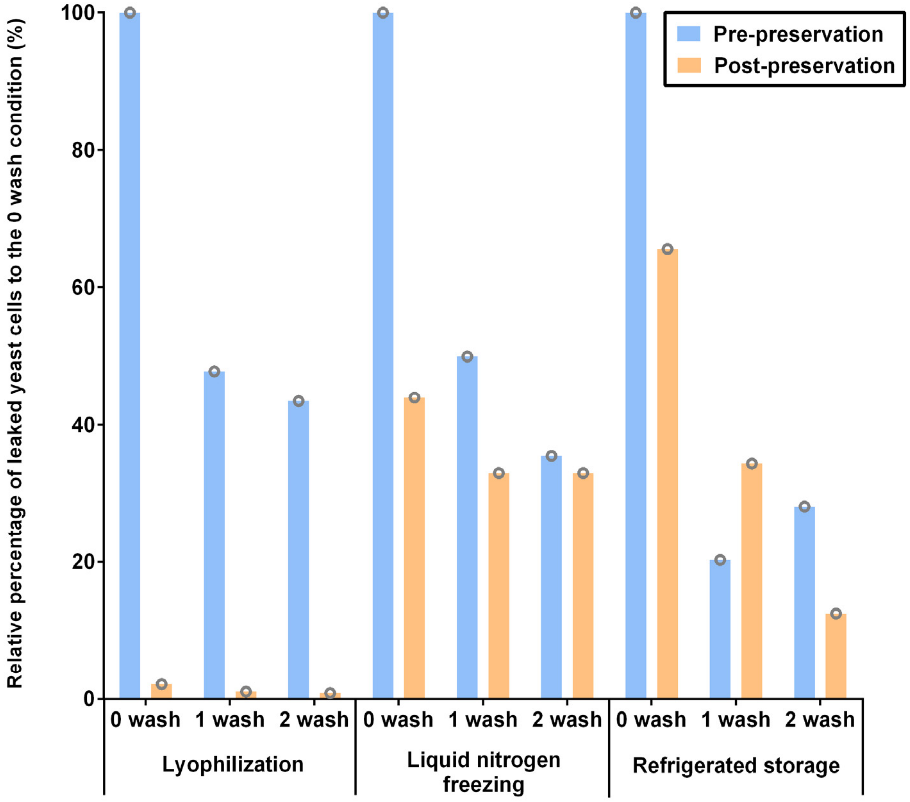
Relative percentage of leaked yeast cells to the 0 wash condition (%)
100
80
60
40
20
0
Pre-preservation
Post-preservation
0 wash
1 wash
2 wash
Lyophilization
Liquid nitrogen freezing
Refrigerated storage

Supplementary Fig. 9. Investigation of consortia activity for betaxanthins production after preservation process. Lyophilization method is mostly used in this study (**A**). Three different preservation methods including lyophilization, refrigerated storage and liquid nitrogen freezing were applied to preservation of consortia-laden polymers. Additional five consecutive uses after preservation process were performed. 30 °C gels after round 5 were split (n=1) for examining the impact of lyophilization and refrigerated storage on betaxanthins production (**B**). **C-D**, The comparison of preservation capacity between liquid culture (n=3) and hydrogel (n=1) for betaxanthins production. Liquid 100 uL or 500 uL condition represent 100 or 500 μL of pre-preserved bulk culture sample was taken and proceeded to different preservation treatments. Fermentation was performed at 30 °C for all conditions. **E,** Production of betaxanthins pre-lyophilization, and post-lyophilization, with an extended period of storage (1 month, n=3). **F,** Comparison of tensile testing and SEM analysis of 30 wt% F127-BUM hydrogels before and after lyophilization. The values for Young's modulus, and stress and strain at the point of material failure under tensile stress (the top figure and table, n=3). The bottom figure is the SEM images of the betaxanthins production gels before (left image) and after (right image) two consecutive rounds of on-demand fermentation. The similarities in morphology and pore size of the hydrogel in both conditions implies that multiple rounds of fermentation does not affect the integrity of the hydrogel material. All images have 30-micron scale bars. **G,** Confocal microscopy of red fluorescing yeast and green fluorescing bacteria pre- and post-lyophilization, showing cell distribution within the hydrogel material unaffected by preservation. All images have 200-micron scale bars. **H**, The impact of washing process for consortia hydrogels on the percentage of leaked *E. coli* (top) and yeast (bottom) cells between pre- and post-preservation was compared (n=1 for each condition). Source data are provided as a Source Data file.

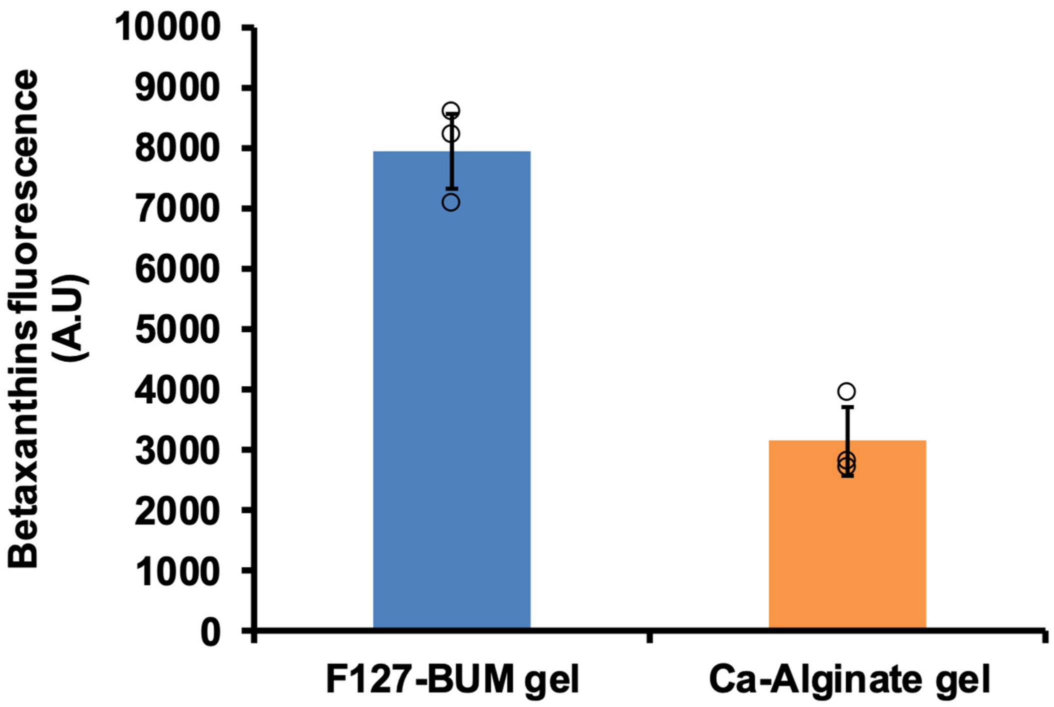

Supplementary Fig. 10. The comparison of maximum betaxanthins production between F127-BUM and calcium alginate hydrogels. Results from the comparison between our F127-BUM hydrogels, and commonly employed calcium alginate hydrogels show that the F127-BUM system is much more effective in the production and release of betaxanthins (2.53-fold increase in efficiency) over a period of 72 hours. Each data point and error bar represent means and standard deviations from biological triplicates, respectively, unless stated otherwise in the Methods section. Source data are provided as a Source Data file.

A

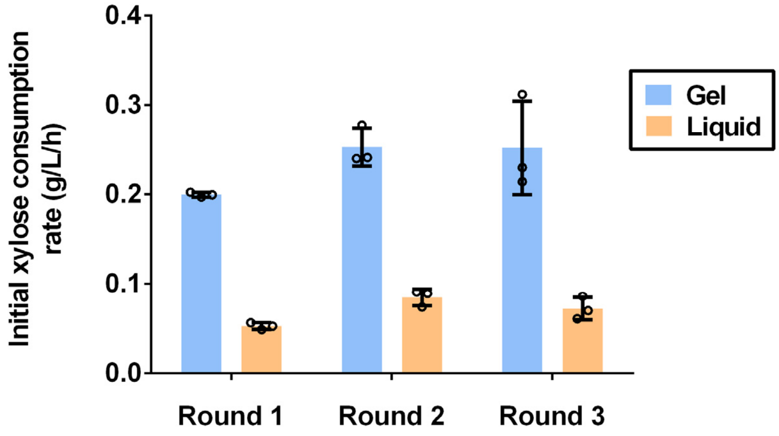

B

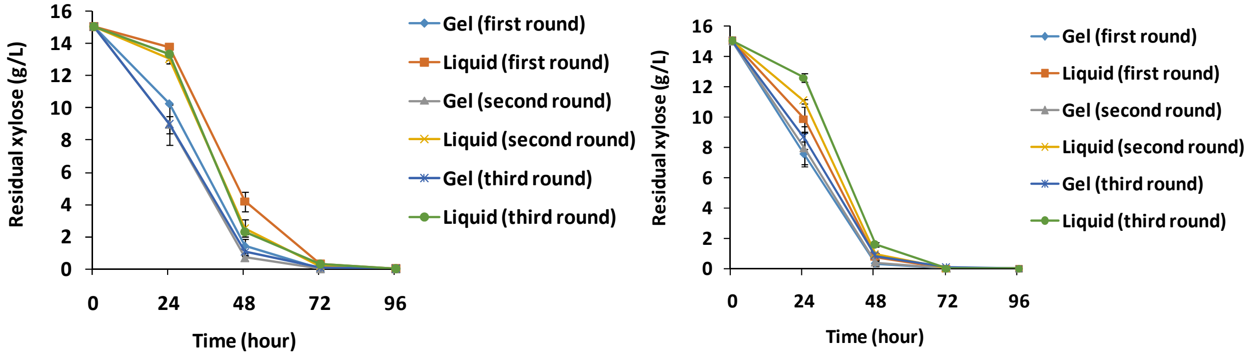

C

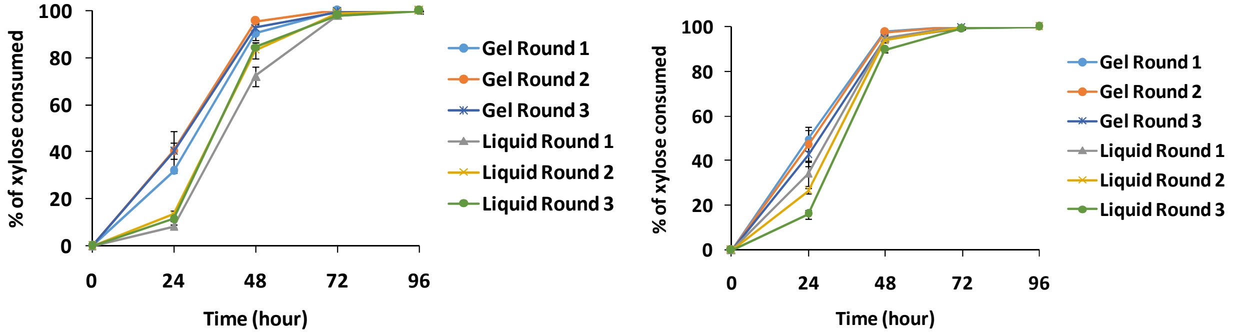

Supplementary Fig. 11. Gel-re-run for xylose/glucose utilization via a parallel yeast-yeast consortium. A, Investigation of consortia activity on glucose/xylose utilization in hydrogel system and liquid culture with re-use. Round 0 was grown in YPD containing 20 g/L glucose to outgrow consortia population. Then all samples were cultivated in YPDX media for three consecutive uses. Xylose consumption rate for each round of re-use was compared. **B,** Time profile of residual xylose

concentration for each round of re-use (round 0 was grown in YPD (left) or YPDX (right)). **C,** Time profile of xylose consumption for each round of re-use (round 0 was grown in YPD (left) or YPDX (right)). Each data point and error bar represent means and standard deviations from biological triplicates, respectively. Source data are provided as a Source Data file.

Supplementary Table 1. List of strains and plasmids used in this study.

Strain/plasmid	Description	Source
E. coli strain		
NEB10β	*Δ(ara-leu) 7697 araD139 fhuA ΔlacX74 galK16 galE15 e14- ϕ80dlacZΔM15 recA1 relA1 endA1 nupG rpsL* (*Str^R*) *rph spoT1 Δ(mrr-hsdRMS-mcrBC)*	New England Biolabs
MG1655	K-12 F– λ– *ilvG– rfb-50 rph-1*	ATCC
BL21(DE3)	*E. coli* str. B F^- *ompT gal dcm lon* $hsdS_B(r_B^- m_B^-)$ *λ(DE3 [lacI lacUV5-T7p07 ind1 sam7 nin5*]) [$malB^+$]$_{K\text{-}12}$(λ^S)	New England Biolabs
CD02	[MG1655] *nfsA*::*BBa J23119-sfGFP*; Kan^R	Dr. Jesse G. Zalatan's lab
MC4100_pHK11	*E. coli* MC4100 carries pHK11containing colicin V gene cluster *cvaAB-cvaC-cvi*; Amp^R	16
DH5α_pET28b	*E. coli* DH5α (ATCC) was transformed with pET28b empty vector; Kan^R	This study
eBL01	*E. coli* BL21(DE3) Δ*tyrR*	This study
eBL04	[eBL01] pET28-pYIBN-*aroG*$^{(fbr)}$-B30rbs-*tyrA*$^{(fbr)}$-tRRNC; Kan^R	This study
eBL0400DT	[eBL04] pCDFDuet-1; Kan^R; Spc^R	This study
eBL0430D	[eBL04] pCDF-pLPP-B30rbs-*hpaB-hpaC*-T7t; Kan^R; Spc^R	This study
eBL0432D	[eBL04] pCDF-pLPP-B32rbs-*hpaB-hpaC*-T7t; Kan^R; Spc^R	This study
S. cerevisiae strain		
BY4741	*MATα SUC2 gal2 mal2 mel flo1 flo8-1 hap1 ho bio1 bio6 his3*Δ1 *leu2*Δ0 *met15*Δ0 *ura3*Δ0	ATCC

CEN.PK2-a	*MAT*a/α; *ura3-52/ura3-52; trp1-289/trp1-289; leu2-3_112/leu2-3_112; his3 Δ1/his3 Δ1; MAL2-8C/MAL2-8C; SUC2/SUC2*	28
S288C	*MATα SUC2 gal2 mal2 mel flo1 flo8-1 hap1 ho bio1 bio6*	ATCC
SO992	*MATa ura3 leu2 trp1 his3, can1R, ADE+*	29
yJS001	[SO992] *mfa2::*pTEF1-*mCherry*	29
JMW001	[BY4741] *trp1::*TDH3p-*mKate2*	3
BY4741_BDO	[BY4741] p416-pFBA1-*NoxE*-tIDP1-pTPI1-*alsD*-tSPG5-pPGK1-*alsS*-tPRM9	3
CEN.PK2-a_BDO	[CEN.PK2-a] p416-pFBA1-NoxE-tIDP1-pTPI1-alsD-tSPG5-pPGK1-alsS-tPRM9	3
sBY08	[BY4741] *leu2::*pGPD-*MjDOD*-tPRM9 (LEU2 integration with URA3 marker)	This study
YSX3	*MATα trp1-112 leu2::LEU2-PsXYL1 ura3::URA3-PsXYL2 Ty3::NEO-PsXYL3*	27
Plasmids		
pET28b-Duet-1	DNA fragment containing two T7 promoters on pRSFDuet-1 was cloned into pET28b empty vector	This study
pCDF-Duet-1	For construction L-DOPA producing plasmids	This study
pCDF-pT7-*tyrA$^{(fbr)}$*-pT7-*aroG$^{(fbr)}$*	FRT flanked kanamycin resistance gene was removed from pCDF-Kan^{FRT}-tyrA$^{(fbr)}$-aroG$^{(fbr)}$	This study
pET28-pT7-*aroG$^{(fbr)}$*-tT7	For construction of pET28-pYIBN-*aroG*$^{(fbr)}$	This study
pET28-pYIBN-*aroG*$^{(fbr)}$	For construction of pET28-pYIBN-*aroG*$^{(fbr)}$-B30rbs-*tyrA*$^{(fbr)}$-tRRNC	This study

pET28-pYIBN-*aroG*$^{(fbr)}$-B30rbs-*tyrA*$^{(fbr)}$-tRRNC	For tyrosine production	This study
pCDF-pLPP-B30rbs-*hpaB*-*hpaC*-T7t	For L-DOPA production testing	This study
pCDF-pLPP-B32rbs-*hpaB*-*hpaC*-T7t	For L-DOPA production testing	This study
pCMC0759	For amplification of *MjDOD* gene	22
P416-pGPD-tPRM9	For construction of p416-pGPD-*MjDOD*-tPRM9	30
p416-pGPD-*MjDOD*-tPRM9	For generating a URA3 integrative cassette employed for betaxanthins production	This study

Supplementary Table 2. List of primers used in this study.

Primer ID	Primer description	Sequence (5'->3')
P1	tyrR_delFwd	TTACGCCGAAGTGCCCGTTTTTCCGTCTTTGTGTCAATGATTGTTGACAGATTCCGGGGATCCGTCGACC
P2	tyrR_delRvs	CATCAGGCATATTCGCGCTTACTCTTCGTTCTTCTTCTGACTCAGACCATGTGTAGGCTGGAGCTGCTTCG
P3	tyrR_delFwd1	CAAAACGCCCAGCGAAAAATAATGCAATATCGGGTGCTGACCGGATATCTTTACGCCGAAGTGCCCG
P4	tyrR_delRvs1	AGCTCTGGCTGTACTGAAAGCATAATTTAATATGCCTGATGGTGTTGCACCATCAGGCATATTCGCGCTTACTC
P5	SeqtyrRf	GATTTCCGTCGTCAGCTTATC
P6	SeqtyrRr	CAGCTGGTGGATGAAATCAC
P7	TyrAroCDFf	TGGTGTCCGACAGCCTGCATTAGGAAAT
P8	TyrAroCDFr	CGCTTATAGTAACGTTTGATTAACG
P9	CDFTyrAroF	CTGGCGTTAATCAAACGTTACTATAAGCGTTTC
P10	CDFTyrAroR	ATTTCCTAATGCAGGCTGTCGGACACCATCGAATGGCGCAAAA
P11	ACYCDuetUP1_F	GGATCTCGACGCTCTCCCT
P12	T7 terminatorR	GCTAGTTATTGCTCAGCGGTG
P13	p28Duet1F	CTGCTGCCACCGCTGAGCA

P14	p28Duet1R	AGTCGCATAAGGGAGAGCGTC
P25	AroGfbrp21F	TGTTTAACTTTAAGAAGGAGATATACATATGAATTATCAGAACGACGATTTACGCATC
P26	AroGfbrp21R	CGCAAGCTTGTCGACGGAGCTCGAATTCTTACCCGCGACGCGCTTTTA
P27	p28AroGF	GAATTCGAGCTCCGTCGA
P28	p28AroGR	ATGTATATCTCCTTCTTAAAGTTAAACAAAATTATTTC
P33	yibNf	AAATTGCATTCCAGTTAACGCG
P34	yibNAroGr	TGCGTAAATCGTCGTTCTGATAATTCATGGGGGGTAACAACTCCC
P35	yibNAroG1f	GTTACCCCCCATGAATTATCAGAACGACGATTTACGCATC
P36	yibNAroG1r	GGAAGAGAGTCAATTCAGGGTGGTGAATTTACCCGCGACGCGCTTTTA
P37	p28yibNAroGf	CGCGGGTAAATTCACCACCCTGAATTGACTC
P38	p28yibNAroGr	AGCCAGCGCGTTAACTGGAATGCAATTTTGAGCGCAACGCAATTAATGTAAG
P39	p28ATf	ATCCTTAGCGAAAGCTAAGGATTTTTTTACCATCTTAGTATATTAGTTAAGTATAAG
P40	p28ATB30r	CTAGTATTTCTCCTCTTTAATCTCTAGAGGAAGAGAGTCAATTCAG
P41	yibNtyrAr2	TTTCCTAATGCAGGAGTCGCATATTACTGGCGATTGTCATTCGC
P42	B30tyrAF	TCTAGAGATTAAAGAGGAGAAATACTAGATGGTTGCTGAATTGACCGC
P43	rrncterm	CCAGTAATATGCGACTCCTGCATTAGGAAAATCCTTAGCGAAAGCTAAGGATTTTTTTA
P67	lppB30	CCCATCAAAAAAATATTCTCAACATAAAAAACTTTGTGTAATACTTGTAACGCTTCTAGAGATTAAAGAGGAGAAATACTAG

P68	lppB32	CCCATCAAAAAAATATTCTCAACATAAAAAACTTTGTGTAATACTTGTAACGCTTCTAGAGTCACACAGGAAAGTACTAG
P69	lppHpaBCB30f	TCTAGAGATTAAAGAGGAGAAATACTAGATGAAACCAGAAGATTTCCGC
P70	lppHpaBCB30r	CAGGCGCGCCGAGCTCGAATTCGGATCCTTAAATCGCAGCTTCCATTTCC
P71	lppHpaBCB32f	CTTCTAGAGTCACACAGGAAAGTACTAGATGAAACCAGAAGATTTCCGC
P72	pCDFlppTcXALf	GGATCCGAATTCGAGCTCG
P73	pCDFlppTcXALr	AGTTTTTTATGTTGAGAATATTTTTTTGATGGGTGAGCGCAACGCAATTAATGTAAG
P74	SpeIMjDODf	GGACTAGTATGAAGGGAACCTACTACATCAAC
P75	EcoRIMjDODr	CGGAATTCTTAGGATCCGTCGGTCTTTTG
P78	NJM675	TCAAAAAGATCCATGTATAATCTTCATTATTACAGCCCTCTTGACCTCTAATCATGAATGTTCTCGGGTGTCGGGGCTGGCTTAACTATG
P79	NJM676	TATGTAGATTGCGTATATAGTTTCGTCTACCCTATGAACATATTCCATTTTGTAATTTCGTGTCGGTCAGTGAGCGAGGAAGCGGAAGAG

The underlined sequence indicates that the nucleotides used to be annealed to the template for PCR amplification.

References

1. Ji, X.-J., Huang, H. & Ouyang, P.-K. Microbial 2,3-butanediol production: A state-of-the-art review. *Biotechnol. Adv.* **29**, 351-364 (2011).
2. Köpke, M. et al. 2,3-Butanediol Production by Acetogenic Bacteria, an Alternative Route to Chemical Synthesis, Using Industrial Waste Gas. *Appl. Environ. Microbiol.* **77**, 5467 (2011).
3. Deaner, M., Holzman, A. & Alper, H.S. Modular Ligation Extension of Guide RNA Operons (LEGO) for Multiplexed dCas9 Regulation of Metabolic Pathways in *Saccharomyces cerevisiae*. *Biotechnology Journal* **13**, 1700582 (2018).
4. González, E. et al. Role of *Saccharomyces cerevisiae* Oxidoreductases Bdh1p and Ara1p in the Metabolism of Acetoin and 2,3-Butanediol. *Appl. Environ. Microbiol.* **76**, 670-679 (2010).
5. Broadley, K.J. The vascular effects of trace amines and amphetamines. *Pharmacol. Ther.* **125**, 363-375 (2010).
6. Miguelez, C., Benazzouz, A., Ugedo, L. & De Deurwaerdère, P. Impairment of Serotonergic Transmission by the Antiparkinsonian Drug L-DOPA: Mechanisms and Clinical Implications. *Front. Cell. Neurosci.* **11** (2017).
7. Surwase, S.N. & Jadhav, J.P. Bioconversion of l-tyrosine to l-DOPA by a novel bacterium *Bacillus* sp. JPJ. *Amino Acids* **41**, 495-506 (2011).
8. Muñoz, A.J. et al. Metabolic engineering of *Escherichia coli* for improving l-3,4-dihydroxyphenylalanine (l-DOPA) synthesis from glucose. *J. Ind. Microbiol. Biotechnol.* **38**, 1845 (2011).
9. Yuan, S.-F. et al. Production of optically pure l(+)-lactic acid from waste plywood chips

using an isolated thermotolerant *Enterococcus faecalis* SI at a pilot scale. *J. Ind. Microbiol. Biotechnol.* **45**, 961-970 (2018).

10. Yuan, S.-F., Guo, G.-L. & Hwang, W.-S. Ethanol production from dilute-acid steam exploded lignocellulosic feedstocks using an isolated multistress-tolerant *Pichia kudriavzevii* strain. *Microbial Biotechnology* **10**, 1581-1590 (2017).
11. Yuan, S.-F. & Alper, H.S. Metabolic engineering of microbial cell factories for production of nutraceuticals. *Microbial Cell Factories* **18**, 46 (2019).
12. Nakagawa, A. et al. A bacterial platform for fermentative production of plant alkaloids. *Nature Communications* **2**, 326 (2011).
13. Kim, S.C., Min, B.E., Hwang, H.G., Seo, S.W. & Jung, G.Y. Pathway optimization by re-design of untranslated regions for L-tyrosine production in *Escherichia coli*. *Sci. Rep.* **5**, 13853-13853 (2015).
14. Huang, Q., Lin, Y. & Yan, Y. Caffeic acid production enhancement by engineering a phenylalanine over-producing *Escherichia coli* strain. *Biotechnol. Bioeng.* **110**, 3188-3196 (2013).
15. Santos, C.N.S. & Stephanopoulos, G. Melanin-Based High-Throughput Screen for L-Tyrosine Production in *Escherichia coli*. *Appl. Environ. Microbiol.* **74**, 1190 (2008).
16. Gilson, L., Mahanty, H.K. & Kolter, R. Four plasmid genes are required for colicin V synthesis, export, and immunity. *J. Bacteriol.* **169**, 2466 (1987).
17. Cascales, E. et al. Colicin Biology. *Microbiol. Mol. Biol. Rev.* **71**, 158 (2007).
18. Gérard, F., Pradel, N. & Wu, L.-F. Bactericidal Activity of Colicin V Is Mediated by an Inner Membrane Protein, SdaC, of *Escherichia coli*. *J. Bacteriol.* **187**, 1945 (2005).
19. Eş, I., Vieira, J.D.G. & Amaral, A.C. Principles, techniques, and applications of biocatalyst

immobilization for industrial application. *Appl. Microbiol. Biotechnol.* **99**, 2065-2082 (2015).

20. Alonso, S. in Novel Food Fermentation Technologies. (eds. K.S. Ojha & B.K. Tiwari) 7-33 (Springer International Publishing, Cham; 2016).

21. Martins, N., Roriz, C.L., Morales, P., Barros, L. & Ferreira, I.C.F.R. Coloring attributes of betalains: a key emphasis on stability and future applications. *Food Funct.* **8**, 1357-1372 (2017).

22. Grewal, P.S., Modavi, C., Russ, Z.N., Harris, N.C. & Dueber, J.E. Bioproduction of a betalain color palette in *Saccharomyces cerevisiae*. *Metab. Eng.* **45**, 180-188 (2018).

23. DeLoache, W.C. et al. An enzyme-coupled biosensor enables (S)-reticuline production in yeast from glucose. *Nat. Chem. Biol.* **11**, 465 (2015).

24. Gírio, F.M. et al. Hemicelluloses for fuel ethanol: A review. *Bioresour. Technol.* **101**, 4775-4800 (2010).

25. Kwak, S. & Jin, Y.-S. Production of fuels and chemicals from xylose by engineered *Saccharomyces cerevisiae*: a review and perspective. *Microbial Cell Factories* **16**, 82 (2017).

26. Moysés, D.N., Reis, V.C.B., de Almeida, J.R.M., de Moraes, L.M.P. & Torres, F.A.G. Xylose Fermentation by *Saccharomyces cerevisiae*: Challenges and Prospects. *Int. J. Mol. Sci.* **17**, 207-207 (2016).

27. Jin, Y.-S., Ni, H., Laplaza, J.M. & Jeffries, T.W. Optimal Growth and Ethanol Production from Xylose by Recombinant *Saccharomyces cerevisiae* Require Moderate D-Xylulokinase Activity. *Appl. Environ. Microbiol.* **69**, 495 (2003).

28. van Dijken, J.P. et al. An interlaboratory comparison of physiological and genetic

properties of four *Saccharomyces cerevisiae* strains. *Enzyme and Microbial Technology* **26**, 706-714 (2000).

29. Saha, A. et al. Additive Manufacturing of Catalytically Active Living Materials. *ACS Applied Materials & Interfaces* **10**, 13373-13380 (2018).

30. Mumberg, D., Müller, R. & Funk, M. Regulatable promoters of *Saccharomyces cerevisiae*: comparison of transcriptional activity and their use for heterologous expression. *Nucleic acids research* **22**, 5767-5768 (1994).
