# Supplementary material for: Compartmentalized microbes and co-cultures in hydrogels for on-demand bioproduction and preservation
Source: Nat Commun. 2020 Feb 4;11:563. doi: 10.1038/s41467-020-14371-4 (PMC7000784; doi:10.1038/s41467-020-14371-4)
Supplement: Supplementary file 2 — Reporting Summary [file 41467_2020_14371_MOESM2_ESM.pdf]

nature research

Corresponding author(s): Nelson and Alper

Last updated by author(s): Nov 18, 2019

Reporting Summary

Nature Research wishes to improve the reproducibility of the work that we publish. This form provides structure for consistency and transparency in reporting. For further information on Nature Research policies, see Authors & Referees and the Editorial Policy Checklist .

Statistics

For all statistical analyses, confirm that the following items are present in the figure legend, table legend, main text, or Methods section.

n/a	Confirmed	
☐	☒	The exact sample size (*n*) for each experimental group/condition, given as a discrete number and unit of measurement
☐	☒	A statement on whether measurements were taken from distinct samples or whether the same sample was measured repeatedly
☐	☒	The statistical test(s) used AND whether they are one- or two-sided<br>*Only common tests should be described solely by name; describe more complex techniques in the Methods section.*
☒	☐	A description of all covariates tested
☐	☒	A description of any assumptions or corrections, such as tests of normality and adjustment for multiple comparisons
☐	☒	A full description of the statistical parameters including central tendency (e.g. means) or other basic estimates (e.g. regression coefficient) AND variation (e.g. standard deviation) or associated estimates of uncertainty (e.g. confidence intervals)
☒	☐	For null hypothesis testing, the test statistic (e.g. *F*, *t*, *r*) with confidence intervals, effect sizes, degrees of freedom and *P* value noted<br>*Give P values as exact values whenever suitable.*
☒	☐	For Bayesian analysis, information on the choice of priors and Markov chain Monte Carlo settings
☒	☐	For hierarchical and complex designs, identification of the appropriate level for tests and full reporting of outcomes
☒	☐	Estimates of effect sizes (e.g. Cohen's *d*, Pearson's *r*), indicating how they were calculated

Our web collection on statistics for biologists contains articles on many of the points above.

Software and code

Policy information about availability of computer code

Data collection	All the data were collected with commercial available software and can be seen in Methods section.
Data analysis	All the data were analyzed with commercial available software and have been mentioned in Methods section.

For manuscripts utilizing custom algorithms or software that are central to the research but not yet described in published literature, software must be made available to editors/reviewers. We strongly encourage code deposition in a community repository (e.g. GitHub). See the Nature Research guidelines for submitting code & software for further information.

Data

Policy information about availability of data

All manuscripts must include a data availability statement. This statement should provide the following information, where applicable:

- Accession codes, unique identifiers, or web links for publicly available datasets
- A list of figures that have associated raw data
- A description of any restrictions on data availability

Data sets will be available upon request.

Field-specific reporting

Please select the one below that is the best fit for your research. If you are not sure, read the appropriate sections before making your selection.

☒ Life sciences ☐ Behavioural & social sciences ☐ Ecological, evolutionary & environmental sciences

For a reference copy of the document with all sections, see nature.com/documents/nr-reporting-summary-flat.pdf

Life sciences study design

All studies must disclose on these points even when the disclosure is negative.

Sample size	No sample size calculation was performed. Each data sets include at least 3 biological replicates, unless stated otherwise in Figure legends and experimental methods.
Data exclusions	No data were excluded.
Replication	Experimental data are presented as the mean ± S.D.
Randomization	No randomization was performed in our study since appropriate control samples were included for comparison.
Blinding	Blinding was not performed in this study. Raw data will be provided upon request.

Reporting for specific materials, systems and methods

We require information from authors about some types of materials, experimental systems and methods used in many studies. Here, indicate whether each material, system or method listed is relevant to your study. If you are not sure if a list item applies to your research, read the appropriate section before selecting a response.

Materials & experimental systems

n/a	Involved in the study
☒	☐ Antibodies
☒	☐ Eukaryotic cell lines
☒	☐ Palaeontology
☒	☐ Animals and other organisms
☒	☐ Human research participants
☒	☐ Clinical data

Methods

n/a	Involved in the study
☒	☐ ChIP-seq
☐	☒ Flow cytometry
☒	☐ MRI-based neuroimaging

Flow Cytometry

Plots

Confirm that:

☐ The axis labels state the marker and fluorochrome used (e.g. CD4-FITC).

☒ The axis scales are clearly visible. Include numbers along axes only for bottom left plot of group (a 'group' is an analysis of identical markers).

☒ All plots are contour plots with outliers or pseudocolor plots.

☒ A numerical value for number of cells or percentage (with statistics) is provided.

Methodology

Sample preparation	E. coli CD02 and S. cerevisiae JMW001 were used for co-culture experiments analyzed using flow cytometry. Flask cultures containing GFP-expressing E. coli and RFP-expressing S. cerevisiae (initially seeded at 1.5E6 cells/mL each) were grown for 20 hours at various temperatures in identical shaking incubators with agitation set to 225 RPM. Culture samples were then diluted in DPBS and held on ice to minimize further growth during analysis.
Instrument	Flow cytometry was performed using a BD LSRII Fortessa instrument.
Software	Flow cytometry was performed using a BD FACSDiva software with GFP and mCherry (RFP) acquisition settings. Data were analyzed using FlowJo_V10.
Cell population abundance	N/A
Gating strategy	Gates were initially drawn based on FSC and SSC signals, and the percentages of each cell type in the resulting scatter-based gates were confirmed based on fluorescence (i.e. GFP for E. coli, RFP for S. cerevisiae).

☒ Tick this box to confirm that a figure exemplifying the gating strategy is provided in the Supplementary Information.
